# Ebselen restores mitochondrial function through metabolic rewiring in Barth syndrome and related mitochondrial diseases

**DOI:** 10.1038/s44321-026-00518-1

**Published:** 2026-10-05

**Authors:** Claire Almyre, Anne-Louise Cayer, Nolwenn Bounaix, Anaïs Hoarau, Marie-Alix Derieppe, Julie Martineau, Pierre Costet, Camille Charles, Mélody Dufossée, Cécile Duplaa, Jim Dompierre, Bénédicte Salin, Marc-Alexandre D’Elia, Corinne Blancard, Aurélie Massoni-Laporte, Stéphane Claverol, Caroline Tokarski, Yaser Hashem, Alexandre Favereaux, Yeranuhi Hovhannisyan, Juliette Jouhet, Cyrille Guimaraes, Jean-François Dumas, Olivier R Baris, Laetitia Dard, Jérémy Richard, Vanessa Bergeron, Naïg Gueguen, Valérie Desquiret-Dumas, Jordan Rivron, Douglas Strathdee, Carole H Sellem, Nathalie Bonnefoy, Laurent Monassier, Estelle Ayme-Dietrich, Onnik Agbulut, Stéphane Azoulay, Jean-Paul di Rago, Vincent Procaccio, François Godard, Déborah Tribouillard-Tanvier

**Affiliations:** 1 Univ. Bordeaux, CNRS, IBGC, UMR 5095, F-33000 Bordeaux, France; 2 Univ Angers, CHU Angers, Inserm, CNRS, MITOVASC, MITOLAB, SFR ICAT, F-49000 Angers, France; 3 U. de Bordeaux, Animalerie Mutualisée, Talence, France; 4 CRYME Centre BROCA Nouvelle Aquitaine (CBNA), Université de Bordeaux service commun des animaleries - Bordeaux, Bordeaux, France; 5 Inserm U1034, Biologie des maladies cardiovasculaires, Pessac, France; 6 Université Côte d’Azur, CNRS UMR 7272 ICN, Nice, France; 7 Univ. Bordeaux, CNRS, Bordeaux Proteome, UAR 2046, F-33076 Bordeaux, France; 8 ARNA Laboratory, European Institute of Chemistry and Biology, U1212 INSERM, UMR5320 CNRS, Université de Bordeaux, Pessac, F-33600 France; 9 University of Bordeaux, CNRS UMR 5297, Interdisciplinary Institute for Neuroscience, Bordeaux, France; 10 Sorbonne Université, Institut de Biologie Paris-Seine, UMR CNRS 8263, Inserm U1345, Development, Adaptation and Ageing, Paris, France; 11 Laboratoire de Physiologie Cellulaire et Végétale, CNRS, CEA, INRAE, Univ. Grenoble Alpes, 17 Avenue des Martyrs, 38000 Grenoble, France; 12 Laboratoire « Niche Nutrition Cancer & métabolisme Oxydatif », Inserm UMR1069, Université de Tours, 10 Boulevard Tonnellé, 37032 Tours, France; 13 Transgenic Technology Laboratory, CRUK Scotland Institute, Glasgow, UK; 14 Université Paris Saclay, CEA, CNRS, I2BC, Gif-sur-Yvette, France; 15 Laboratoire de Pharmacologie et Toxicologie Neurocardiovasculaire, UR7296, Université de Strasbourg, 67000 Strasbourg, France

**Keywords:** Genetics, Gene Therapy & Genetic Disease, Organelles, Pharmacology & Drug Discovery

## Abstract

Despite major advances in the genetic diagnosis of mitochondrial disorders, effective disease-modifying therapies remain scarce. Here, we identify ebselen (EBS) as a promising therapeutic candidate through a phenotypic drug screen in a yeast model of Barth syndrome, a cardiomyopathy caused by defective cardiolipin maturation. EBS improved oxidative phosphorylation-dependent growth across diverse fungal models of mitochondrial diseases, including defects in complex I, complex IV, mitochondrial DNA maintenance, mitochondrial translation, and ATP synthase. Therapeutic efficacy was further validated in patient-derived fibroblasts, iPSC-derived cardiomyocytes, and a cardiolipin-deficient mouse model. Mechanistically, EBS acts independently of its established antioxidant activity by engaging a conserved metabolic program that suppresses cytosolic translation while stimulating pyruvate dehydrogenase-dependent tricarboxylic acid cycle activity, thereby improving proteostasis and mitochondrial bioenergetic function. These effects are likely coordinated through lysosome-associated mTOR signaling, consistent with the localization of EBS to lysosomes and its colocalization with mTOR. Together, our findings identify a conserved mechanism for enhancing mitochondrial function and establish ebselen as a strong candidate for therapeutic repurposing in Barth syndrome and a broad spectrum of inherited mitochondrial disorders.

## Synopsis

Ebselen (EBS) improves mitochondrial function across genetically distinct mitochondrial disease models through conserved mTOR and metabolic pathways, supporting its potential as a genotype-independent therapeutic strategy.

- Mitochondrial function was improved by EBS across seven fungal and three human mitochondrial disease models, independently of the underlying genetic defect.
- Exercise capacity was increased approximately threefold in BTHS mice treated with EBS (10 mg/kg, twice daily), accompanied by improved cardiac mitochondrial respiration and ATP synthesis.
- EBS was shown to act independently of cardiolipin restoration, antioxidant activity, IMPase inhibition, OXPHOS assembly, and mitochondrial biogenesis.
- EBS was found to accumulate in lysosome-associated mTOR compartments, while conserved EBS-responsive pathways were identified across species and mTOR activation was functionally validated as required for mitochondrial rescue.
- Cytosolic translation was moderately reduced by approximately 25%, while PDC regulation was shifted toward activation through reduced PDK4 and increased PDP1 abundance.

## Abstract (web summary)

Ebselen (EBS) improves mitochondrial function across genetically distinct mitochondrial disease models through conserved mTOR and metabolic pathways, supporting its potential as a genotype-independent therapeutic strategy.

## Graphical abstract

**Figure:**
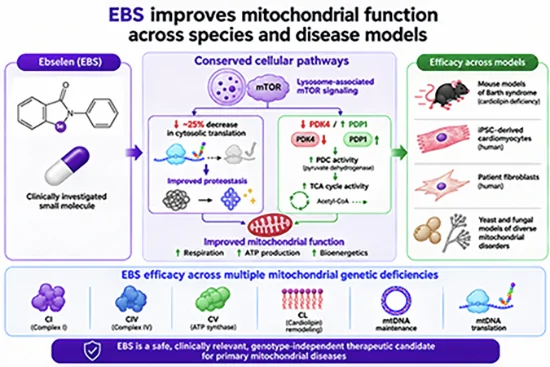

The paper explained **Problem**

Primary mitochondrial diseases are rare and genetically diverse disorders with few effective treatment options. Barth syndrome, caused by defects in the *TAZ* gene, leads to mitochondrial dysfunction and cardiomyopathy, and despite recent therapeutic advances, major unmet medical needs remain.

**Results**

We identified ebselen (EBS), a clinically investigated compound, as a potent modulator of mitochondrial function. EBS improved mitochondrial function across diverse genetic defects and experimental models, from fungi to patient-derived cells, iPSC-derived cardiomyocytes, and mice. These effects involve conserved pathways linking lysosome-associated mTOR signaling, cytosolic translation, and mitochondrial metabolism. In Barth syndrome mice, EBS markedly improved exercise capacity, cardiac mitochondrial respiration, ATP synthesis, and cardiac ultrastructure.

**Impact**

EBS emerges as a promising genotype-independent therapeutic strategy for primary mitochondrial diseases, providing strong preclinical support for its clinical repurposing.

## Introduction

Mitochondrial disorders (MD) represent a highly heterogeneous group of inherited metabolic diseases that affect approximately 1 in 5000 individuals. They can manifest at any age and in any organ, reflecting the central role of mitochondria in energy metabolism. Despite considerable progress in their diagnosis over the past decade, effective and broadly applicable treatments are still lacking. Current therapeutic options are mostly symptomatic, and no drug has yet proven capable of restoring mitochondrial function across different disease contexts. Identifying a compound capable of improving mitochondrial energy metabolism independently of the underlying genetic defect has long been a major goal in the field.

Barth syndrome (BTHS) is one of these rare MD. This X-linked recessive disease, affecting approximately one male per million, is characterized by cardiomyopathy, skeletal myopathy, neutropenia, growth delay, and 3-methylglutaconic aciduria, leading to a high infant mortality rate due to cardiac and immune defects (Barth et al, 1983; Barth et al, 1996; Taylor et al, 2022). BTHS results from mutations in the *TAZ* gene encoding Tafazzin, a key enzyme in cardiolipin (CL) remodeling within the inner mitochondrial membrane. Loss of Tafazzin activity reduces mature CL levels and leads to monolysocardiolipin (MLCL) accumulation, impairing oxidative phosphorylation (OXPHOS) and ATP production : this is particularly detrimental in the heart, where mitochondria provide 95% of the energy demand (Ikon and Ryan, 2017; Pfeiffer et al, 2003). Curative treatments are still lacking since elamipretide, the only FDA-approved drug for BTHS, shows so far only modest positive effects in patients (Thompson et al, 2024).

Yeast (de Taffin de Tilques et al, 2018; de Taffin de Tilques et al, 2017) and human cellular models of Barth syndrome (Almyre et al, 2025; Wang et al, 2014) recapitulate BTHS biochemical defects, including mitochondrial energy decrease and elevated MLCL/CL ratios, while a BTHS mouse model (Sohn et al, 2022; Wang et al, 2020; Wang et al, 2023) also displays fatigue, endurance and cardiac defects, and growth delay. We looked for small molecules effective on these BTHS models using a screening method that we successfully developed (Almyre et al, 2025; de Taffin de Tilques et al, 2018). Amongst chemical libraries of compounds at least in phase II clinical trials, we identified Ebselen (EBS), an organoselenium compound with known antioxidant and anti-inflammatory properties (Belvisi et al, 2000; Capper et al, 2018; Muller et al, 1984; Ren et al, 2018), as a particularly promising candidate. EBS has demonstrated beneficial effects in models of neurodegenerative (Cabungcal et al, 2014; Moussaoui et al, 2000; Xie et al, 2017), metabolic (Polianskyte-Prause et al, 2022), infectious (Gustafsson et al, 2016; Lu et al, 2013; Thangamani et al, 2017), viral (Jin et al, 2020; Simanjuntak et al, 2018; Thenin-Houssier et al, 2016), and cancer-related diseases (Feng et al, 2020; Kim et al, 2019; Santofimia-Castano et al, 2018), and has been investigated in phase III clinical trials for bipolar disorder (Fenn et al, 2020; Sharpley et al, 2020), and Ménière’s disease (Kil et al, 2022). Notably, the phase III trial for Ménière’s disease was recently completed with positive outcomes, further supporting the clinical potential of EBS and showing safety, good bioavailability, and blood–brain barrier penetration (Santi et al, 2021; Takasago et al, 1997).

Here, we demonstrate that EBS improves mitochondrial function in yeast, human cells, and mouse models of BTHS. Importantly, its beneficial effects are independent of its antioxidant activity in Ménière’s disease and of inositol monophosphatase inhibition proposed in bipolar disorder. Instead, our data support a mechanism involving modulation of cytosolic translation and stimulation of the tricarboxylic acid (TCA) cycle. Moreover, EBS proved to be active in yeast and human cellular models of other MD, including those with defects in complex IV, mtDNA maintenance, mitochondrial translation, complex I and ATP synthase, corresponding to Leigh syndrome, cytochrome c oxidase deficiency, hepatocerebral syndrome, mitochondrial encephalomyopathy with lactic acidosis and stroke-like episodes (MELAS), neuropathy, ataxia, and retinitis pigmentosa (NARP) and maternally inherited Leigh syndrome (MILS). Collectively, these findings identify EBS as a broadly effective modulator of mitochondrial function and a promising drug candidate for numerous MD.

## Results

### EBS improves mitochondrial function in yeast and human models of Barth syndrome

**Figure 1:**
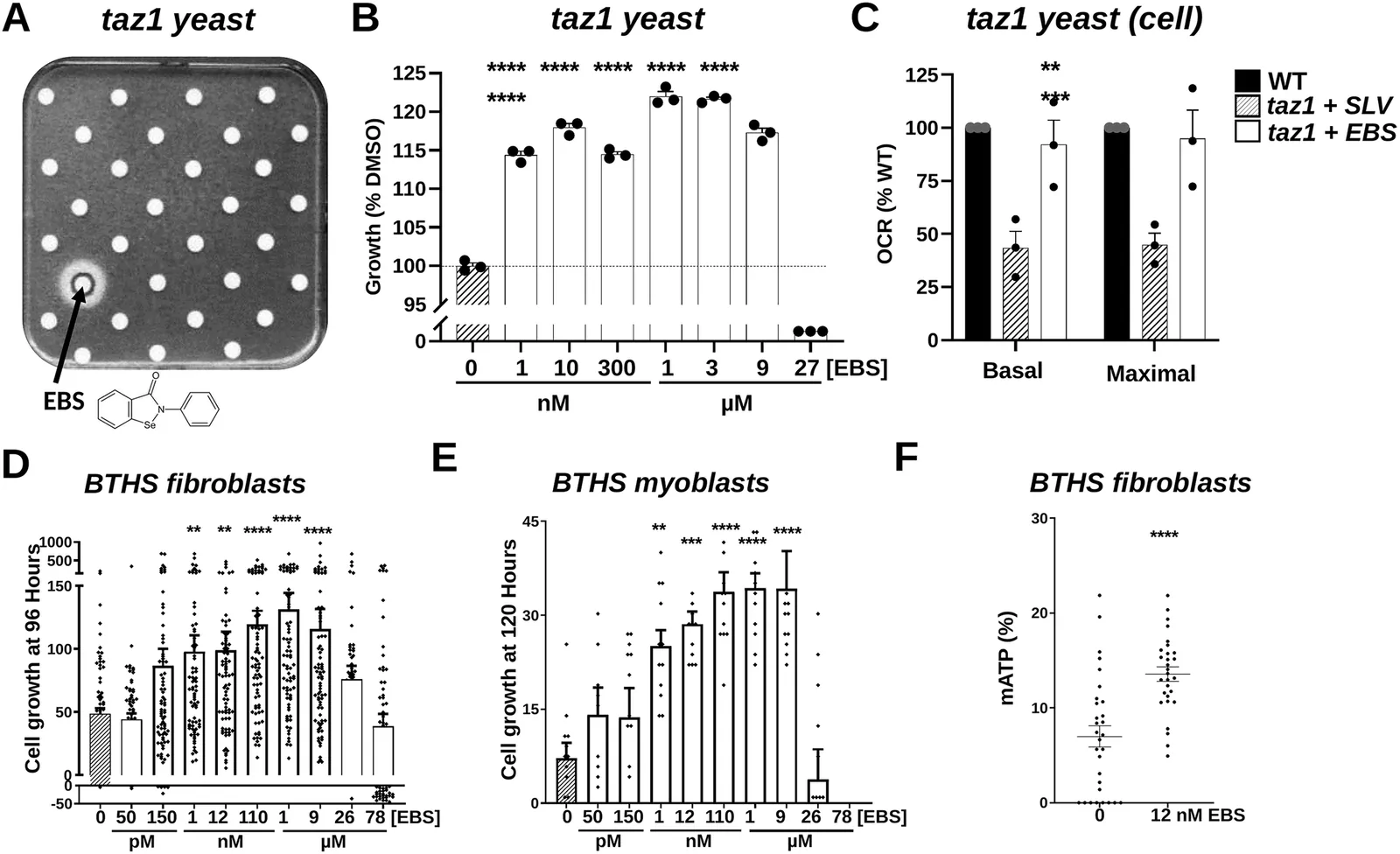
EBS improves mitochondrial function in yeast and human models of Barth syndrome. (**A**) Identification of EBS through chemical drug screening. 0.125 OD of the yeast *taz1* mutant freshly grown in glucose-based media were homogeneously spread with sterile glass beads onto a solid respiratory medium in a square Petri dish (12 cm × 12 cm). Cells were then exposed to 20 nmoles of compounds from FDA-approved chemical libraries (Prestwick and TebuBio), dissolved in 2 µL DMSO and spotted on paper disks. DMSO alone served as a negative control (top left filter on each plate). Plates were incubated at 36 °C for 4–5 days and then photographed. The chemical structure of EBS is shown. (**B**). EBS is active over a wide concentration range. The *taz1* mutant, pregrown in rich glucose medium at 28 °C, was inoculated into liquid respiratory medium supplemented with various concentrations of EBS or DMSO (vehicle control). Cultures were incubated at the restrictive temperature of 36 °C with constant shaking, and cell density (OD₆₀₀ nm) was measured at regular intervals over 4 days. Data were normalized to the DMSO-treated control (noted as 0). (**C**) EBS improves oxygen consumption in whole cells. Oxygen consumption rates (OCR) were measured in whole *taz1* cells, grown for 7–8 generations at 36 °C in respiratory medium in the absence (DMSO, hatched bars) or presence of 1 µM EBS (white bars). Wild-type (WT) cells are shown in black bars. Basal or uncoupled maximal OCR were measured in whole cells without substrate addition (Basal) or CCCP (Maximal), respectively. Non-mitochondrial respiration, measured after complex III inhibition by antimycin A, was subtracted from all OCR values. Data in (**B**, **C**) represent three independent experiments. (**D**,** E**) Proliferation of BTHS patient cells in the presence or absence of EBS on (**D**) fibroblasts after 96 h in LG medium and on (**E**) myoblasts after 120 h in HG medium. Cells were seeded in a 384-well plate (1200/well) and treated with the indicated concentrations of EBS or DMSO as a vehicle control (noted as 0). Growth values are expressed relative to the number of cells at T0 and represent the mean of independent experiments. Data were collected from eight fibroblasts, one myoblast, three experiments each, and four technical replicates per condition. For (**B**–**E**), statistical significance was assessed by one-way ANOVA followed by Dunnett’s multiple-comparisons test. Statistical comparisons are shown for EBS-treated versus untreated mutant cells. **p < 0.01, ***p < 0.001, ****p < 0.0001. (**F**). ATP production in BTHS patient fibroblasts treated or not with 12 nM EBS for 96 h. Cells (5000/well in 96-well plates) were incubated in the presence of antimycin A (100 μM), rotenone (0.5 μM), or oligomycin A (3 μM), and ATP levels were quantified using the CellTiter-Glo Promega kit. The percentage of mitochondrial ATP is shown. Data were collected from eight fibroblasts, three experiments each, and four technical replicates per condition. Statistical significance was assessed by the Mann–Whitney test for comparisons between EBS-treated and DMSO-treated cells. **p < 0.01, ***p < 0.001, ****p < 0.0001. Values are given as means ± SEM. Exact *P* values for all statistical comparisons are provided in Appendix Table S1. Source data are available online for this figure.

To identify compounds capable of restoring mitochondrial function in Barth syndrome (BTHS), we screened a library of FDA-approved molecules using our previously established *Saccharomyces cerevisiae* model lacking the cardiolipin-remodeling enzyme Taz1 (Lasserre et al, 2015). Ebselen (EBS) emerged as one of the most active compounds, producing a marked halo of respiratory growth restoration in the filter diffusion assay (Fig. 1A). Several structural analogs of EBS were tested in parallel but showed little or no activity, indicating that the beneficial effect is not a general property of this chemical family (Appendix Fig. S1A). In liquid cultures, EBS significantly improved the respiratory growth of the *taz1* mutant over a broad concentration range (1 nM to 9 µM), whereas higher concentrations became toxic, revealing a wide therapeutic window (Fig. 1B). Consistent with the improvement in respiratory growth, EBS also significantly increased mitochondrial oxygen consumption in the yeast model (Fig. 1C). We next investigated whether these effects were conserved in human cells derived from BTHS patients. Similar to the yeast model, EBS promoted the proliferation and mitochondrial ATP content of both patient fibroblasts and myoblasts over a comparable concentration range (Fig. 1D–F). Again, beneficial effects were observed across a broad range of concentrations before toxicity became apparent at the highest doses tested. Together, these data show that EBS improves mitochondrial function in both yeast and human models of BTHS and retains a favorable therapeutic window across species.

### EBS does not restore cardiolipin abundance but alters cardiolipin molecular composition

**Figure EV1:**
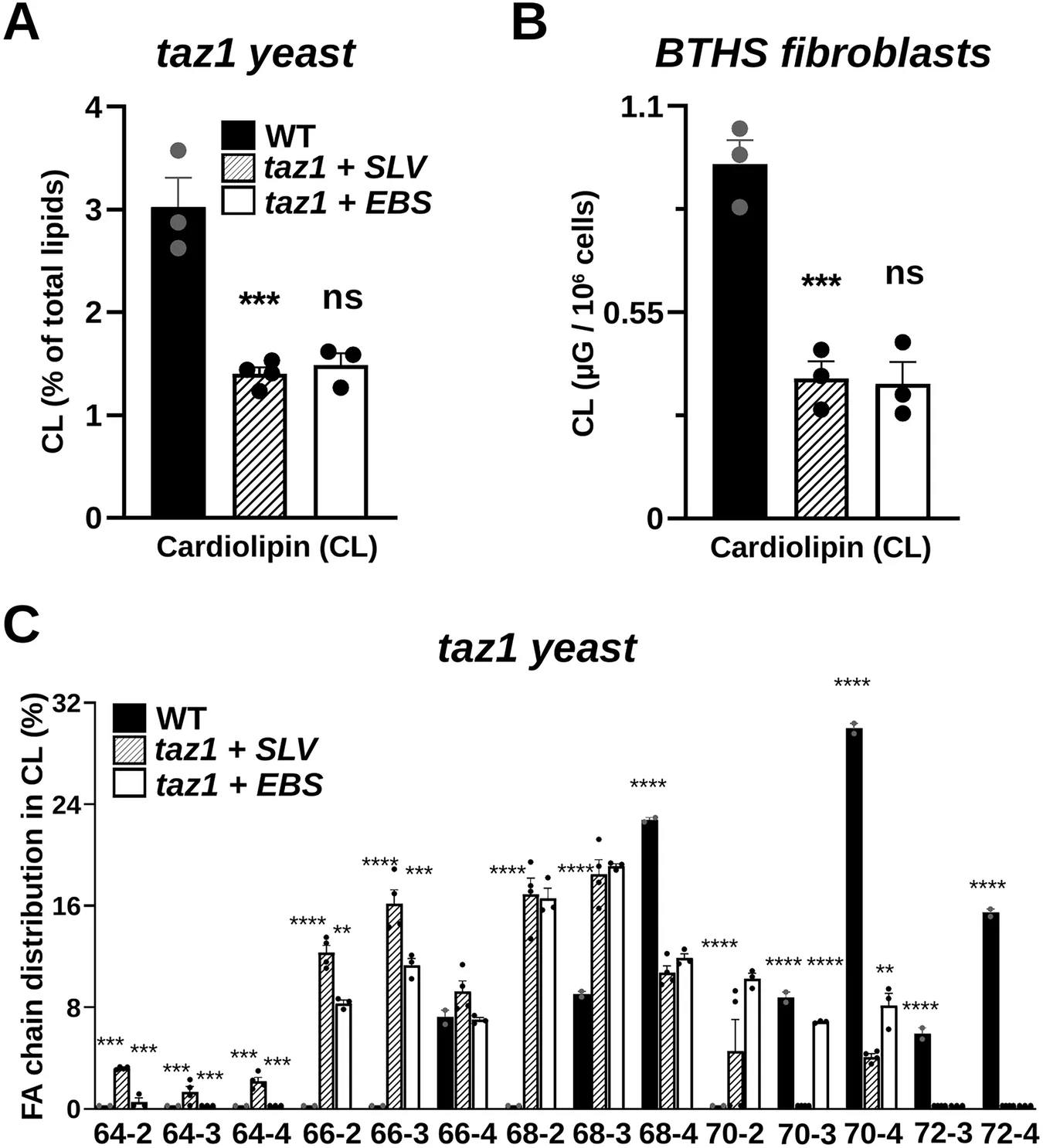
EBS does not restore cardiolipin abundance but alters cardiolipin molecular composition. (**A**) Representative glycerolipid compositions of mitochondrial membranes from BTHS yeast model, treated with solvent (DMSO) or EBS. The distribution of CL is given in percent of total lipid mass. (**B**) Representative distribution of CL in control and BTHS fibroblasts treated with solvent (DMSO) or EBS. Control fibroblasts treated with solvent are shown in the black bar, solvent-treated BTHS fibroblasts as hatched, and EBS-treated BTHS fibroblasts as white bars. (**C**) Fatty acid chain distribution of cardiolipin in the BTHS yeast model, solvent or EBS-treated. The first number indicates the number of carbons, and the second number refers to the number of double bonds (unsaturations). Values are expressed in percentages of the total fatty acid content. WT cells are shown in the black bar, mutant cells treated with solvent are shown in the hatched bar and mutant cells treated with EBS are shown in the white bar. For (**A**–**C**), the reported values are means calculated from 3 independent experiments ±SEM. Statistical significance of differences with DMSO-treated mutant cells was estimated using one-way ANOVA followed by Dunnett’s multiple-comparisons test. **p < 0.01, ***p < 0.001, ****p < 0.0001.

Since defective cardiolipin (CL) remodeling is the primary biochemical hallmark of Barth syndrome, we next investigated whether the beneficial effects of EBS resulted from restoration of CL homeostasis. As expected, the *taz1* yeast mutant displayed a marked reduction in mature CL, consistent with defective tafazzin activity. Likewise, patient-derived BTHS fibroblasts contained significantly lower amounts of CL than control cells. However, EBS treatment did not restore CL abundance in either model, indicating that the improvement in mitochondrial function does not result from correction of the primary lipid defect (Expanded View (EV) Fig. EV1A,B). We therefore examined whether EBS instead affected the molecular composition of the remaining CL pool. Lipidomic analysis of the yeast model revealed a shift toward CL species containing longer and more unsaturated acyl chains following EBS treatment, partially counteracting the abnormal molecular profile associated with the loss of Taz1 (Fig. EV1C). These findings indicate that EBS improves mitochondrial function without restoring total CL levels, suggesting that its mechanism of action is independent of the primary defect in CL remodeling. These observations further suggest that the beneficial effects of EBS are unlikely to be restricted to tafazzin deficiency, raising the possibility that the compound targets a more general mechanism regulating mitochondrial function.

### EBS improves mitochondrial function across multiple models of mitochondrial disease

**Figure 2:**
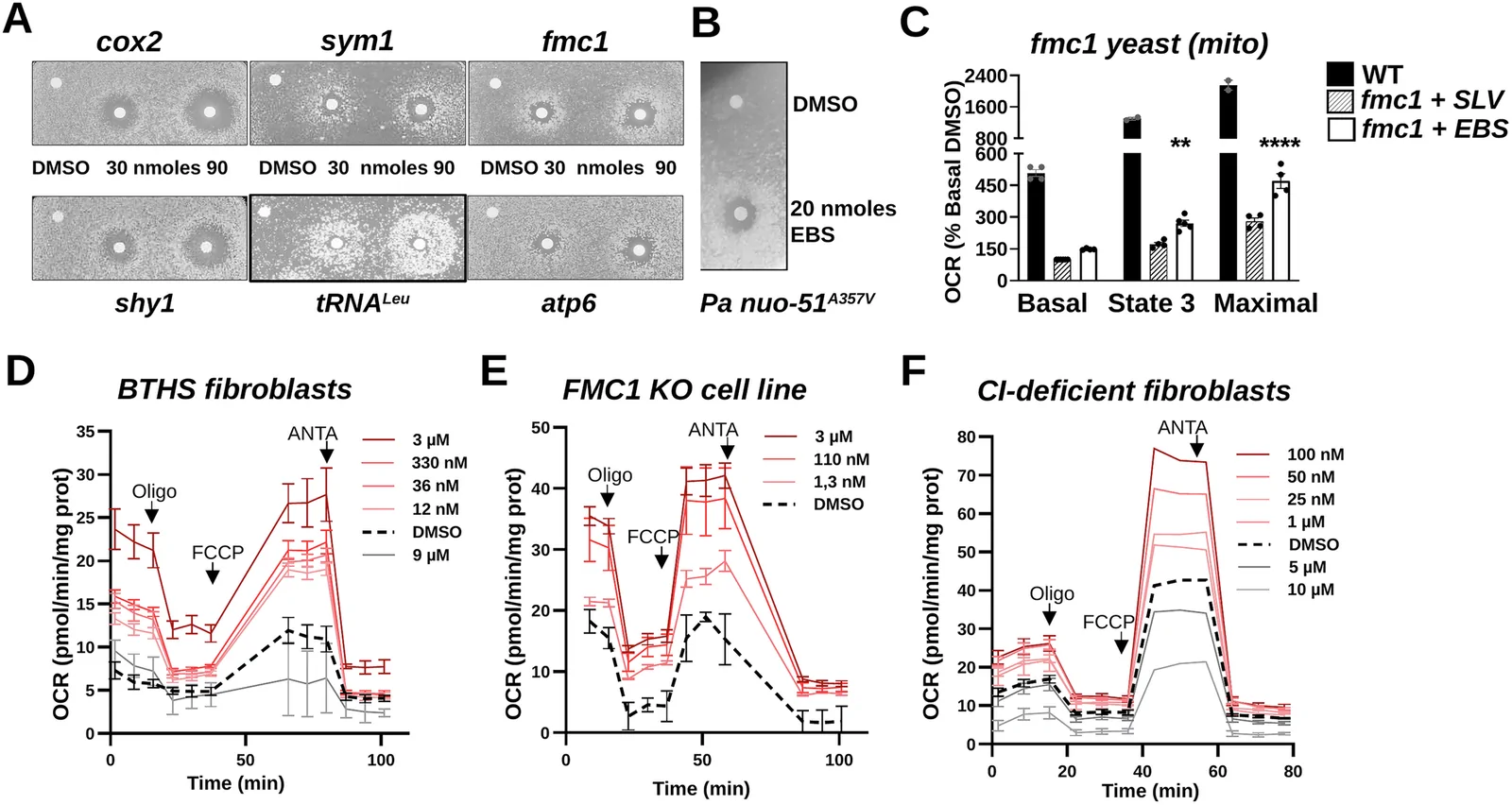
EBS improves mitochondrial function across multiple models of mitochondrial disease. (**A**, **B**) EBS is active in various fungal models of MD in a dose-dependent manner. Six Sc (**D**) and one Pa (**E**) models tested in this study (Table 1) were spread as dense layers on solid respiratory medium and then exposed to paper disks loaded with the indicated quantities of EBS for 4–7 days at temperatures appropriate to each strain (Table 2). Images were taken at the end of the incubation period. (**C**) EBS improves oxygen consumption in isolated mitochondria. Oxygen consumption rates (OCR) were measured in isolated mitochondria from *fmc1* mutants, grown for 7–8 generations at 36 °C in respiratory medium in the absence (DMSO, hatched bars) or presence of 1 µM EBS (white bars). Wild-type (WT) cells are shown in black bars. State 3 respiration was measured in the presence of NADH and ADP, and uncoupled respiration was recorded following ATP synthase inhibition with oligomycin and full uncoupling with CCCP. Non-mitochondrial respiration, measured after complex III inhibition by antimycin A, was subtracted from all OCR values. Data represent three or four independent experiments; statistical comparisons are shown for EBS-treated versus untreated mutant cells. Values are given as means ± SEM. Statistical significance was assessed by one-way ANOVA followed by Dunnett’s multiple-comparisons test. **p < 0.01, ****p < 0.0001. Exact *P* values for all statistical comparisons are provided in Appendix Table S1. (**D**–**F**) EBS improves mitochondrial respiration in a dose-dependent manner in several human models of MD: (**D**) BTHS fibroblasts, (**E**) FMC1 knockout HEK cells, and (**F**) CI patient fibroblasts. Cells were treated or not for 96 h with increasing concentrations of EBS, and oxygen consumption rates (OCR) were measured using the Seahorse XF Mito Stress Test. OCR was recorded at baseline (Basal), after ATP synthase inhibition with oligomycin (Oligo), after uncoupling with FCCP (Uncoupled), and after inhibition of complex III with antimycin A (ANTA) to assess non-mitochondrial respiration. Increasing EBS concentrations are shown in order of increasing efficacy. Data represent means ± SEM of three or four independent experiments. Source data are available online for this figure.

Since the beneficial effects of EBS did not rely on restoration of CL abundance, we asked whether its activity could extend beyond Barth syndrome. To address this question, we evaluated EBS in a panel of fungal and human models carrying defects affecting distinct mitochondrial pathways. In addition to the *taz1* mutant, EBS improved respiratory growth in six *Saccharomyces cerevisiae* models with defects in cytochrome c oxidase biogenesis and assembly (*cox2* (Bonnefoy et al, 2001) and *shy1* (Almyre et al, 2025)), mitochondrial DNA maintenance (*sym1*, (Almyre et al, 2025)), mitochondrial translation (*tRNA*^*Leu*^, (De Luca et al, 2009)), and ATP synthase assembly and biogenesis (*fmc1*, (Lefebvre-Legendre et al, 2001) and *atp6*, (Rak et al, 2007)) (Fig. 2A). Since *Saccharomyces cerevisiae* lacks a complex I (CI) homologous to that of humans, EBS was also tested in a model of the filamentous fungus *Podospora anserina* carrying a mutation (*Pa nuo-51A357V*), which models human CI deficiency (Schuelke et al, 1999). Strikingly, EBS was found to be effective in this model as well (Fig. 2B). Although the magnitude of growth rescue and sensitivity to EBS varied between models, all fungal models responded positively over a broad range of concentrations before growth inhibition became apparent at the highest doses tested. *taz1, sym1, fmc1*, and *atp6* mutants showed broadly comparable responses, whereas *cox2* and *shy1* were more sensitive to high EBS concentrations despite clear growth rescue at lower doses. Conversely, *tRNA*^*Leu*^ and *Pa nuo-51A357V* models displayed lower apparent toxicity and relatively stronger growth improvement. Because growth kinetics and assay conditions differ between models, direct quantitative comparisons should be interpreted with caution. Consistent with these growth phenotypes, EBS also significantly increased mitochondrial oxygen consumption in the *fmc1* mutant (Fig. 2C).

We next investigated whether this broad activity was conserved in human cells. As observed in the fungal models, EBS significantly increased mitochondrial oxygen consumption in BTHS fibroblasts (Fig. 2D), CRISPR/Cas9-generated *FMC1* knockout cells (Fig. 2E), and patient-derived fibroblasts carrying an isolated CI deficiency (Fig. 2F). In all three cellular models, mitochondrial respiration was significantly improved over a broad concentration range, from nanomolar to low micromolar concentrations, whereas toxicity became apparent from 5 µM. Thus, the beneficial effects of EBS are not restricted to tafazzin deficiency but extend to genetically distinct MD affecting multiple respiratory pathways.

### EBS has beneficial effects in complex I-deficient iPSC-derived cardiomyocytes

**Figure 3:**
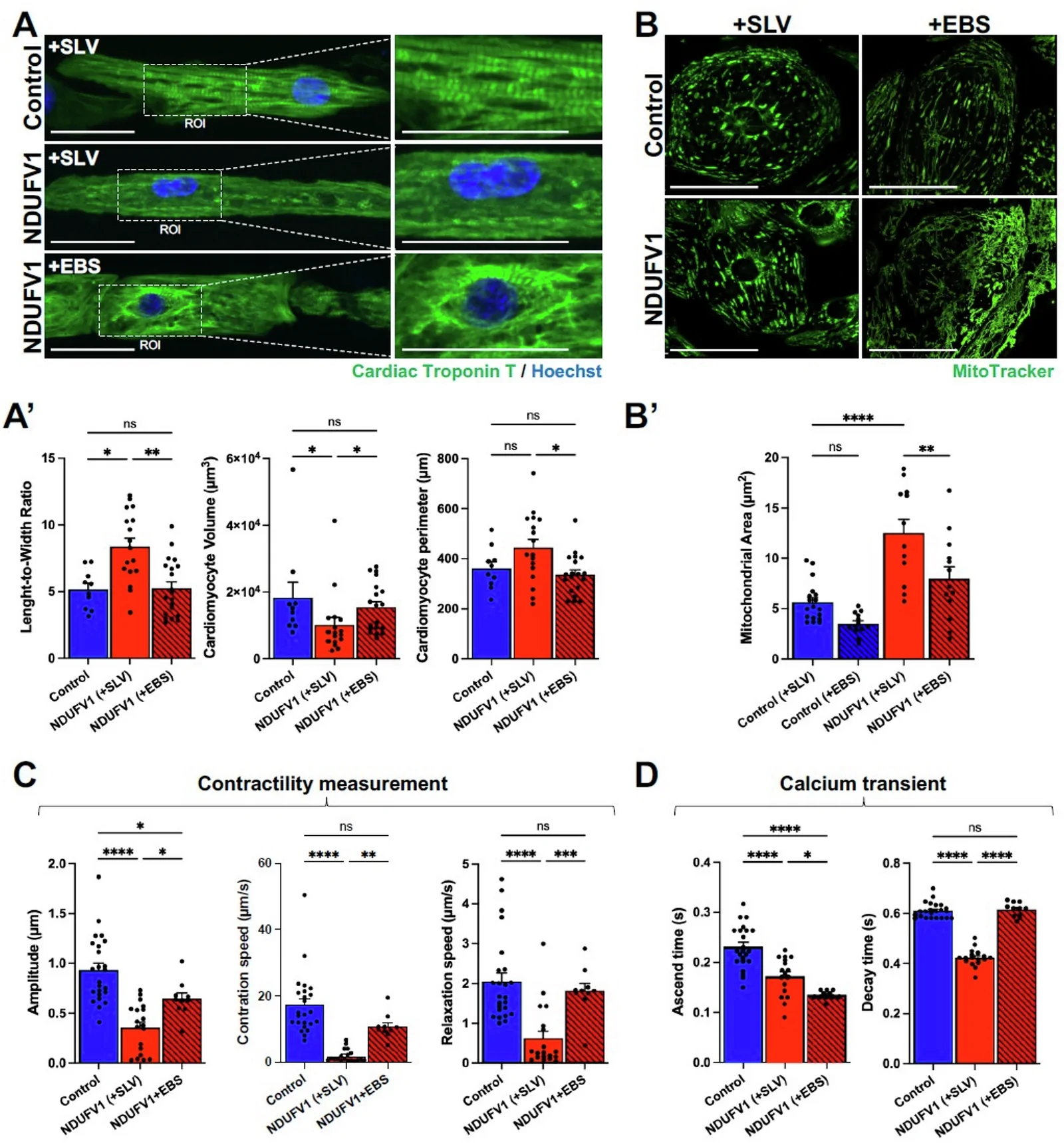
EBS has beneficial effects in cardiomyocytes differentiated from Complex I-deficient iPSCs. (**A**) Immunostaining for cardiac Troponin T (green) was performed on cardiomyocytes cultured on Matrigel-coated micropatterned substrates (20-µm-wide lines) for 14 days post-thawing. Nuclei were counterstained with Hoechst (blue). Scale bar: 50 µm. (**A’**) Cardiomyocyte length-to-width ratio, cardiomyocyte volume and perimeter (calculated based on cardiac troponin T staining) were measured using ImageJ software. Values are given as means ± SEM. Control + SLV was analyzed in a single batch (*N* = 1, *n* = 10 cells), and on two independent differentiation batches for NDUFV1 clone 17 + SLV (*N* = 2, *n* = 18 cells) and NDUFV1 clone 17 + 20 nM EBS (*N* = 2, *n* = 20 cells). (**B**) Mitochondrial network of control and NDUFV1 mutant cardiomyocytes treated or not with EBS. Scale bar: 50 µm. (**B’**) Mitochondrial area in NDUFV1 mutated cardiomyocytes was measured. Bar diagrams show the distribution in %. Values are given as means ± SEM. Control + SLV was analyzed in a single batch (*N* = 1, *n* = 18 cells), and on two independent differentiation batches for NDUFV1 clone 17 + SLV (*N* = 2, *n* = 12 cells) and NDUFV1 clone 17 + 20 nM EBS (*N* = 2, *n* = 13 cells). (**C**) Image-based evaluation of cardiac spheroid contractility using custom-developed MATLAB scripts. Contraction amplitude, contraction speed and relaxation speed were measured on Control and NDUFV1 mutant cardiomyocytes treated or not with EBS. Values are given as means ± SEM. Control + SLV was analyzed in 1 batch (*N* = 1, *n* = 24 spheroids), NDUFV1 clone 17 + SLV in 1 batch (*N* = 1, *n* = 19 spheroids), and NDUFV1 clone 17 + 20 nM EBS in 1 batch (*N* = 1, *n* = 10 spheroids). (**D**) Image-based evaluation of calcium transient in spheroids using custom-developed MATLAB scripts. Ascent and decay times of calcium transients were measured on Control and NDUFV1 mutant cardiomyocytes treated or not with EBS. Values are given as means ± SEM. Control + SLV was analyzed in 1 batch (*N* = 1, *n* = 23 spheroids), NDUFV1 clone 17 + SLV in 1 batch (*N* = 1, *n* = 16 spheroids), and NDUFV1 clone 17 + 20 nM EBS in 1 batch (*N* = 1, *n* = 11 spheroids). SLV corresponds to the DMSO vehicle control. All experiments shown were performed using patient-derived iPSC clone 17. Statistical significance was assessed by the Mann–Whitney test for comparisons between EBS-treated and DMSO-treated cells. *p < 0.05, **p < 0.01, ***p < 0.001, ****p < 0.0001. Exact *P* values for all statistical comparisons are provided in Appendix Table S1. Morphological findings were independently confirmed in a second patient-derived iPSC clone (clone 18) (Appendix Fig. S2). Source data are available online for this figure.

Given that MD associated with cardiomyopathy, including Barth syndrome and respiratory chain defects, affects cardiac structure and function, we next sought to evaluate the effects of EBS in a physiologically relevant human cardiac model. While we were unable to obtain stable cardiomyocytes derived from Barth syndrome iPSCs due to their limited cardiomyogenic potential, we therefore focused on cardiomyocytes derived from iPSCs of a patient carrying biallelic NDUFV1 variants, as a representative model of respiratory chain deficiency. To assess the morphological consequences of these variants, iPSCs from a healthy donor (control) and from the patient were differentiated into cardiomyocytes and cultured on micropatterned surfaces (20 µm-wide lines) to promote sarcomeric alignment. As shown in Fig. 3A, control cardiomyocytes displayed a well-organized sarcomeric structure, whereas NDUFV1-mutant cardiomyocytes exhibited marked sarcomeric disorganization. Remarkably, EBS treatment led to a partial restoration of sarcomere organization in mutant cardiomyocytes. To further quantify these morphological alterations and the effects of EBS treatment, we measured cardiomyocyte length-to-width ratio, volume, and perimeter (Fig. 3A’). The length-to-width ratio was significantly increased in mutant cardiomyocytes, consistent with a dilated cardiomyopathy–like phenotype. Upon EBS treatment, the length-to-width ratio decreased in mutant cardiomyocytes. In line with this observation, EBS also restored both cell volume and perimeter toward control values (Fig. 3A’). Importantly, to strengthen these findings, we repeated the analysis using an independent iPSC clone from the same genetic background, which yielded consistent results (Appendix Fig. S2). We next examined the effects of EBS on the mitochondrial network in mutant cardiomyocytes. Mitochondrial staining revealed that, following EBS treatment, mitochondria appeared less fused and the overall network organization returned to a state closer to that observed in control cardiomyocytes (Fig. 3B,B’). Beyond these structural effects, functional analyses revealed a significant improvement in contractile performance and calcium handling upon EBS treatment. In NDUFV1-mutant cardiomyocytes, both contraction amplitude and the speeds of contraction and relaxation were markedly reduced compared to control cells, whereas EBS treatment restored these parameters toward control levels (Fig. 3C). Similarly, calcium transient analysis revealed a reduced decay time in NDUFV1-mutant cardiomyocytes, consistent with altered calcium handling, which was fully restored to control levels following EBS treatment (Fig. 3D). Collectively, these data indicate that EBS improves both structural and mitochondrial features in cardiomyocytes with mitochondrial respiratory chain dysfunction.

### EBS has beneficial effects in a BTHS mouse model with defects in cardiolipin remodeling

**Figure EV2:**
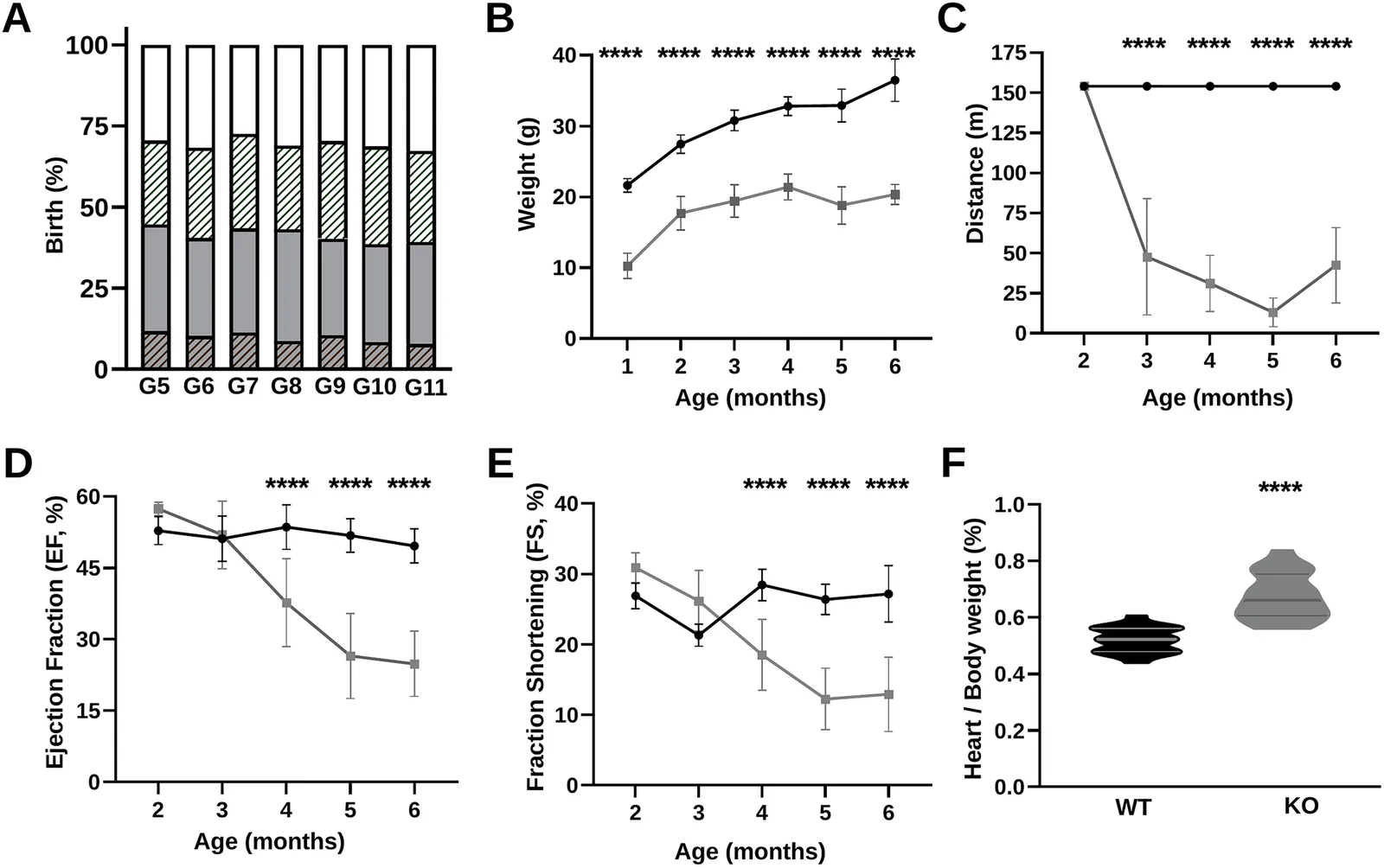
Characterization of the BTHS mouse model at the whole animal level. (**A**) Survival of TAZ-KO mice. TAZ-KO mice were present at below the expected Mendelian ratio from birth. Percentage of births from generation 8 to generation 11. WT females are in white bars; HET females are in white striped bars; WT males are in gray bars; KO males are in striped gray bars. (**B**) Growth curve from 1 month to 6 months. Statistical significance between the TAZ-KO and WT groups is shown. *n* is between 18 and 55 according to age. (**C**) Decreased endurance in male TAZ KO mice from 3 months of age. Measurement of treadmill running distance of male WT (*n* = 10 to 52) and KO (*n* = 10 to 32) mice from 2 to 6 months of age. (**D**, **E**) Cardiomyopathy in male TAZ KO mice at 4 months of age. Cardiac parameters measured by echocardiography in male WT (*n* = 7 to 19) and KO (*n* = 7 to 20) mice from 2 to 6 months of age, with measurements of ejection fraction (EF) (**D**) and fraction shortening (FS) (**E**). (**F**) Cardiac hypertrophy in male TAZ KO mice. Ratio of heart weight to animal weight of male TAZ KO mice (*n* = 18) compared to male WT mice (*n* = 18). For (**B**–**F**), data were presented as mean ± SEM and statistical analyses are performed for TAZ KO mice (gray lines) versus WT mice (black lines) with ***p < 0.001 and ****p < 0.0001.

To build upon the results obtained in yeast and human cell models, we next evaluated the therapeutic potential of EBS in a mouse model of BTHS, a key step in translating these findings toward a clinical trial. To generate male Taz-KO (Taz^∆/Y^) mice, heterozygous females (Taz^∆/+^) were crossed with WT male C57BL/6J mice, since TAZ-deficient males are sterile. This breeding strategy produced WT males and females, Taz-KO (Taz^∆/Y^) males and heterozygous females (Taz^∆/+^). However, the C57BL/6 J strain harbors a mutation in the NNT gene, which encodes nicotinamide nucleotide transhydrogenase, a key mitochondrial enzyme required for NADPH production and antioxidant defense. To avoid any potential interference from the NNT mutation in mitochondrial analyses, we backcrossed the mouse line into the C57BL/6N background. From the 6th generation onward, the mice were considered almost fully congenic with the 6N background. Characterization of this model revealed several phenotypes consistent with previous reports in other genetic backgrounds (Tomczewski et al, 2023; Wang et al, 2023). These included a sub-Mendelian frequency of Taz-KO male (about 6%), suggesting partial embryonic lethality, a reduced body weight from birth (25%), a reduced adipose tissue accumulation, a progressive exercise intolerance from 3 months of age (as assessed by treadmill performance), and a ventricular dysfunction detectable by echocardiography from 4 months of age (Fig. EV2). At the tissue level, hearts from Taz-KO mice displayed hallmarks of BTHS features, including reduced CL levels, accumulation of MLCL, disorganized muscle fibers and mitochondrial structure, and impaired mitochondrial bioenergetics, including reduced ATP production, oxygen consumption and defective OXPHOS complex assembly (Appendix Fig. S3 and Movies EV1 and EV2). It is important to highlight that these phenotypes closely mirror those observed in BTHS patients, particularly the marked skeletal muscle weakness and fatigue. Exercise intolerance, often assessed clinically using the 6-min walk test, is a key outcome measure in drug trials for BTHS and MD more generally. In patients, this limitation is likely due to an impaired capacity to increase oxygen consumption during exercise, resulting from both reduced cardiac reserve and skeletal muscle impairment. Interestingly, in our mouse model, we observed a clear dissociation between cardiac function and treadmill performance, suggesting that cardiac insufficiency may not be the primary driver of exercise limitation.

**Figure 4:**
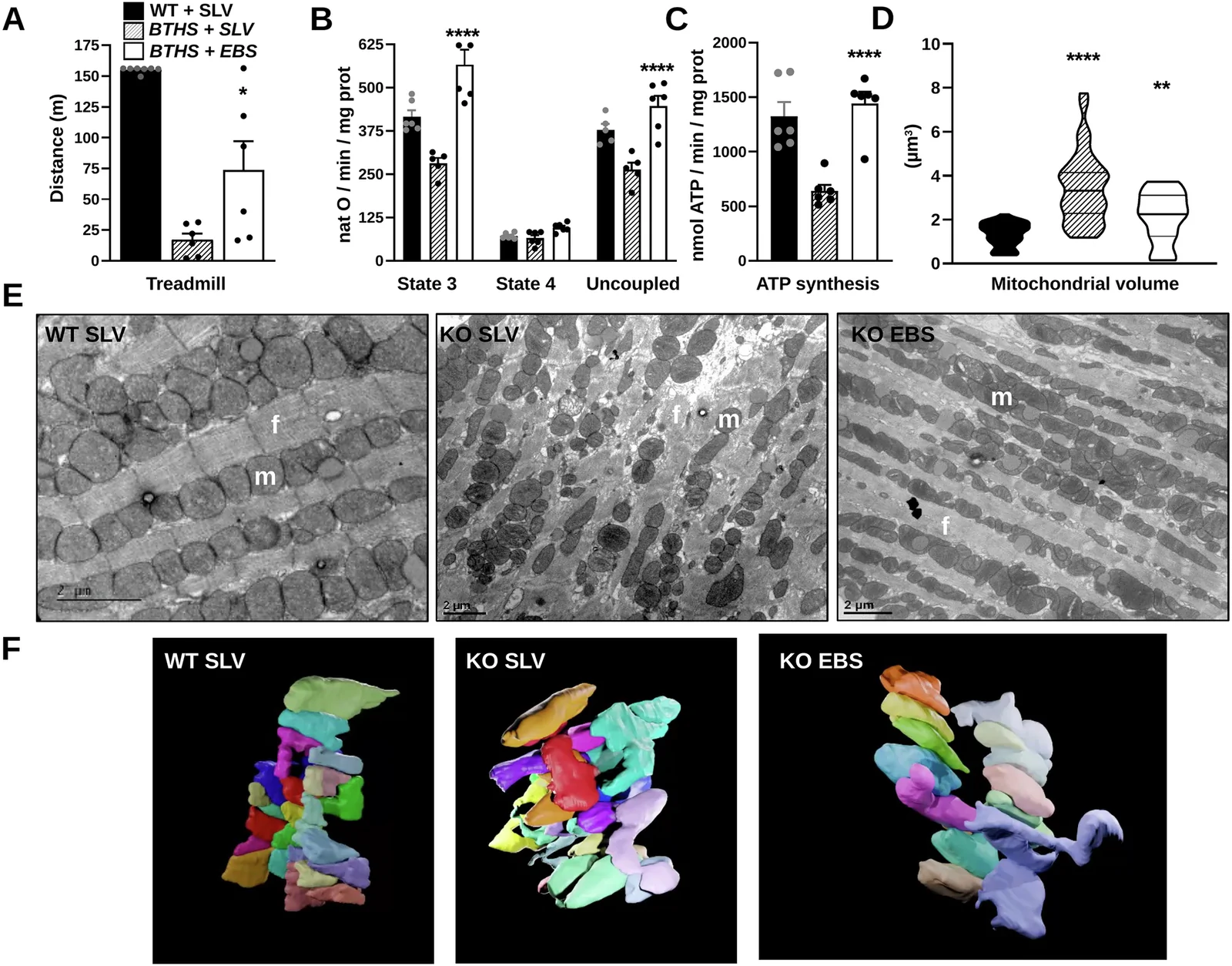
EBS improves mitochondrial function and endurance performance in a mouse model of BTHS treated with 10 mg/kg twice daily from weaning until three months of age. (**A**) Effect of EBS on endurance capacity. Endurance was assessed using a treadmill test in 3-month-old mice: WT treated with solvent (WT), Taz-KO treated with solvent (BTHS + SLV), and Taz-KO treated with EBS (BTHS + EBS). Mice were subjected to forced uphill running until exhaustion. Total running distance is shown. Statistical significance was assessed by one-way ANOVA followed by Tukey’s multiple-comparisons test. (**B**) Effect of EBS on mitochondrial oxygen consumption. Respiration of intact heart mitochondria was measured in the presence of mitochondrial CI substrates (PGM: pyruvate 10 mM, glutamate 5 mM, malate 5 mM). Respiration was recorded under state 3 (phosphorylating condition, ADP + Pi), state 4 (non-phosphorylating conditions, oligomycin), and uncoupled (CCCP titration). Statistical significance was assessed by one-way ANOVA followed by Sidak’s multiple-comparisons test. (**C**) Effect of EBS on ATP synthesis. ATP production by intact heart mitochondria was measured in the presence of mitochondrial CI substrates (PGM: pyruvate 10 mM, glutamate 5 mM, malate 5 mM) and 2.5 mM ADP. Statistical significance was assessed by one-way ANOVA followed by Dunnett’s multiple-comparisons test. For (**A**–**C**), data were expressed as mean ± SEM with *n* = 6. Statistical comparisons are shown between EBS-treated and untreated mutant cells. (**D**) Effect of EBS on mitochondrial volume in heart tissue. Intact heart tissue from WT treated-solvent, KO treated-solvent and KO treated-EBS were fixed, sliced and imaged by transmission electron microscopy (TEM). The volume of 100 mitochondria for each condition was measured. Data were presented as violin plots. Statistical significance was assessed between EBS-treated and solvent-treated BTHS mice using one-way ANOVA followed by Dunnett’s multiple-comparisons test. For (**A**–**D**), WT treated-solvent are in black bar, KO treated-solvent are in hatched bar, and KO treated-EBS are in white bar. (**E**) Effect of EBS on fiber and mitochondrial organization in heart tissue. Intact heart tissue from WT treated-solvent (left panel), KO treated-solvent (middle panel) and KO treated-EBS (right panel) were fixed, sliced and imaged by TEM. Representative images are shown and the same representative image from WT SLV is shown in Appendix Fig. S3F. m mitochondria, f fibers. Scale bars are at 2 µm as indicated. (**F**) Serial block-face imaging. 3D reconstruction movies illustrate mitochondrial ultrastructure from the different conditions. *p < 0.05, **p < 0.01, ***p < 0.001, ****p < 0.0001. Exact *P* values for all statistical comparisons are provided in Appendix Table S1. Source data are available online for this figure.

To test the efficacy of EBS, we selected the most reproducible and robust functional readouts, including treadmill endurance capacity, mitochondrial oxygen consumption and ATP synthesis in isolated cardiac mitochondria, and structural organization of cardiac tissue. Pharmacokinetic (PK) analyses of EBS revealed a short half-life of ~2–3 h, and a relatively low plasmatic concentrations after 10 mg/kg single oral administration (Table EV1). We therefore implemented a twice-daily oral gavage regimen (10 mg/kg) from weaning to three months of age. Remarkably, Taz-KO mice treated with EBS showed substantial improvement across all assessed parameters. Endurance capacity increased approximately threefold in treadmill tests (Fig. 4A), mitochondrial oxygen consumption in isolated cardiac mitochondria was significantly enhanced under both phosphorylating and maximal uncoupled conditions (Fig. 4B), and ATP synthesis capacity was restored (Fig. 4C). Morphological analyses further demonstrated recovery of mitochondrial volume to levels comparable to wild-type controls (Fig. 4D). At the tissue level, wild-type hearts displayed well-organized muscle fibers and mitochondria arranged in parallel arrays, whereas Taz-KO hearts showed severe disorganization with misaligned fibers and dispersed mitochondria. Strikingly, EBS treatment restored a more ordered architecture, with aligned fibers and properly positioned mitochondria resembling the wild-type organization (Fig. 4E,F and Movies EV3–EV5).

### A 2.5-fold higher EBS dose maintains beneficial effects without adverse outcomes in a BTHS mouse model

**Figure EV3:**
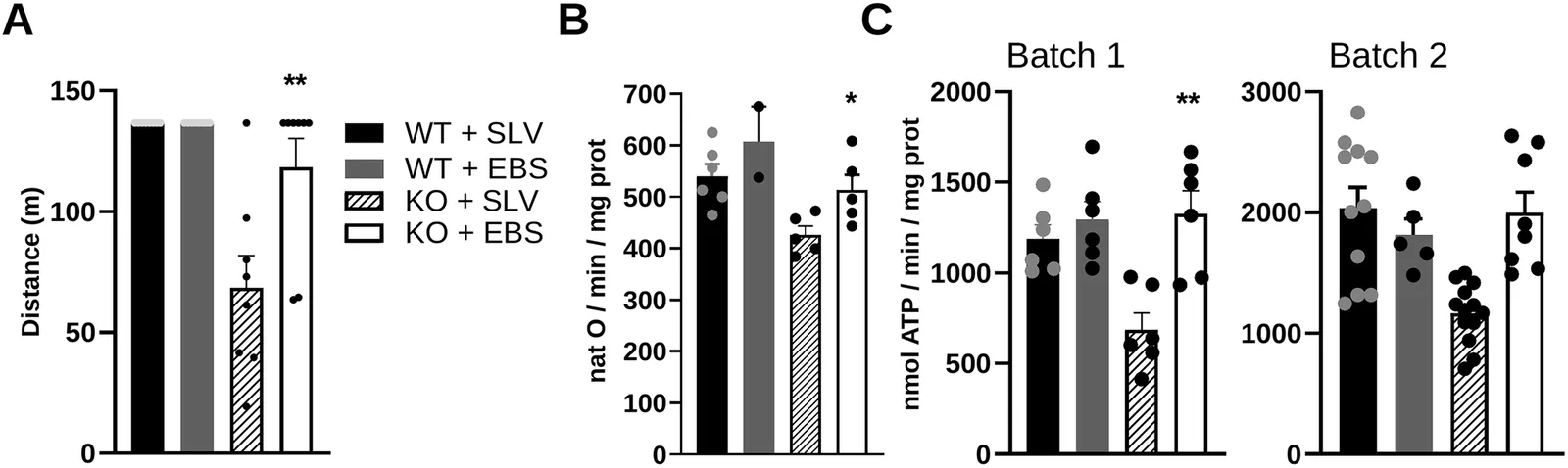
EBS improves mitochondrial function and exercise capacity in a mouse model of BTHS at 50 mg/kg once daily from weaning until three months of age. (**A**) Effect of EBS on endurance capacity. Endurance performance was evaluated using a treadmill test. Three-month-old WT treated with solvent (WT SLV), WT treated with EBS (WT EBS), Taz-KO treated with solvent (KO SLV), and Taz-KO treated with EBS (KO EBS) were subjected to forced uphill running until exhaustion. Muscle performance was measured as total running distance. (**B**) Effect of EBS on mitochondrial oxygen consumption. Respiration in intact heart mitochondria was performed in presence of mitochondrial complex II substrate (succinate 10 mM) and respiration was assessed under state 3 (phosphorylating condition, ADP + Pi). (**C**) Effect of EBS on ATP synthesis. ATP production in intact heart mitochondria was measured in presence of mitochondrial CI substrates (PGM: pyruvate 10 mM, glutamate 5 mM, malate 5 mM) and 2.5 mM ADP. Data were expressed as mean ± SEM with *n* = 6 (2 batches of at least *n* = 6 for ATP synthesis analyses). Statistical significance of differences with DMSO-treated mutant cells was estimated using one-way ANOVA followed by Dunnett’s multiple-comparisons test. *p < 0.005, **p < 0.01. WT SLV is shown in black bar, WT EBS in gray, KO SLV in hatched, and KO EBS in white.

Following the positive outcomes observed with EBS administered at 10 mg/kg twice daily, we next assessed whether a higher dose could enhance efficacy or reveal potential toxicity. A 2.5-fold increased dose of EBS (50 mg/kg, once daily to minimize gavage stress) was administered to BTHS mice and continued to produce beneficial effects on both endurance and cardiac mitochondrial function. In this experiment, WT mice were also treated with EBS and showed no evidence of either beneficial or adverse effects (Fig. EV3). These findings confirm a dose-dependent response to EBS and suggest that higher doses remain well tolerated, with no apparent signs of toxicity or overdose-related side effects. Overall, these results further support EBS as a promising therapeutic candidate for MD and provide a strong rationale for its progression toward clinical evaluation.

### EBS does not restore OXPHOS protein abundance, complex assembly, or mitochondrial biogenesis

**Figure EV4:**
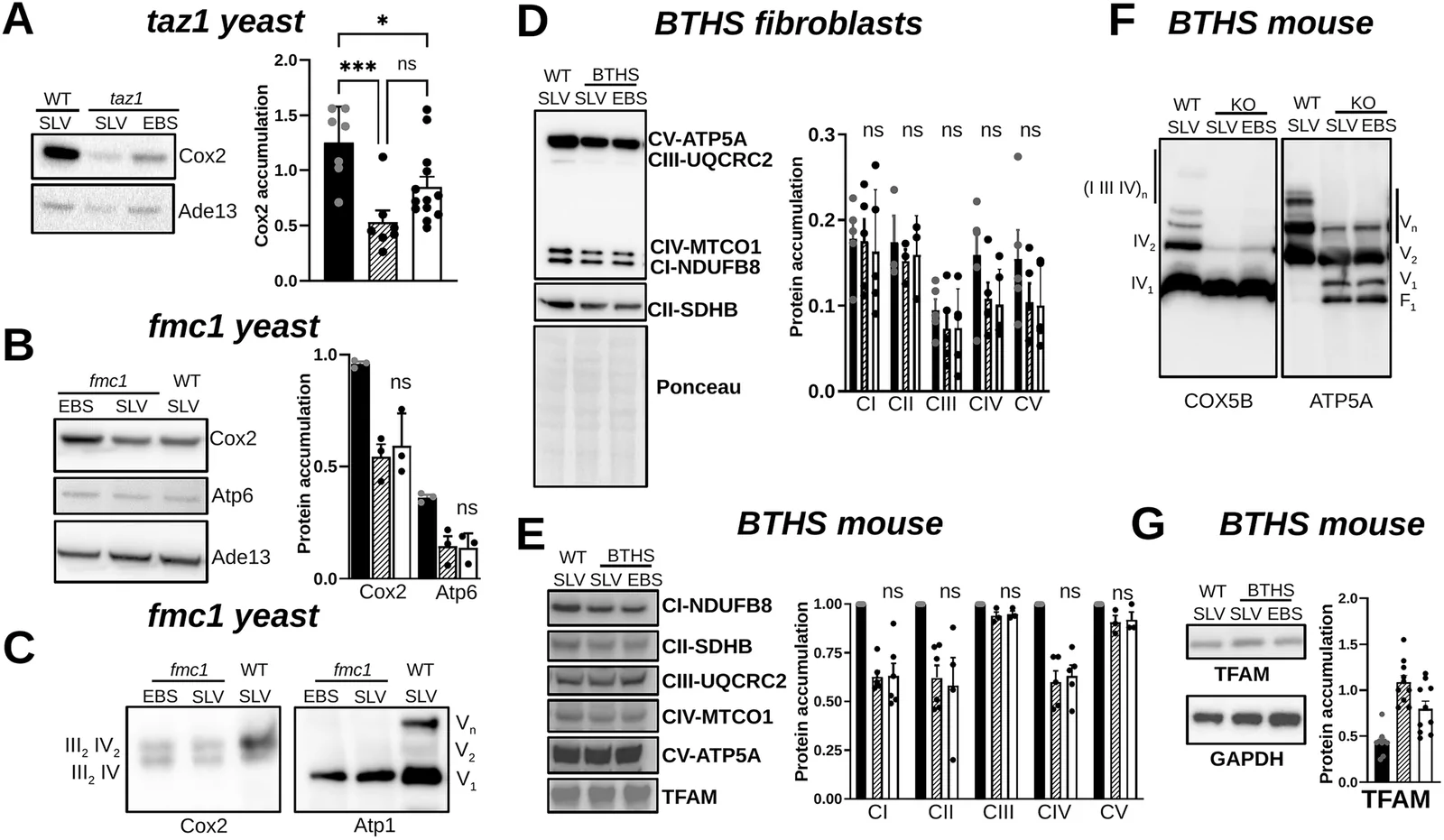
EBS does not enhance OXPHOS protein accumulation or assembly. (**A**,** B**) Steady-state levels of proteins in yeast. Total protein extracts from *taz1* (**A**) and *fmc1* (**B**) yeast were separated by SDS-PAGE, transferred onto nitrocellulose membranes, and probed with the indicated antibodies. Protein levels were normalized to Ade13. Mutant cells treated with solvent (SLV) or EBS were compared. Quantification is shown in the right panels. Values represent the mean ± SEM from at least four independent experiments. One dot represents one independent experiment. WT samples are represented by black bars, solvent-treated mutant samples by hatched bars, and EBS-treated mutant samples by white bars. (**C**) Assembly of OXPHOS complexes in yeast. Mitochondrial proteins from *fmc1* yeast cells treated with solvent (SLV) or 1 µM EBS, and from wild-type cells, were separated using Blue Native-PAGE on a 3–12% polyacrylamide gradient and probed with the indicated antibodies. The immunological signals corresponding to ATP synthase oligomers (Vn), dimers (V2), and monomers (V1) and immunological signals corresponding to supercomplexes (III_2_IV_2_, III_2_IV_1_) are indicated. (**D**,** E**) Steady-state levels of proteins in human cells and mouse heart. Total protein extracts from BTHS patient fibroblasts (**D**) and mitochondrial protein extracts from BTHS mouse hearts (**E**) were analyzed by SDS-PAGE and immunoblotting. Protein levels were normalized to Red Ponceau (**D**) and TFAM (**E**). Quantification is shown in the right panels. Values represent the mean ± SEM from at least four independent experiments. Statistical analyses were restricted to comparisons between BTHS SLV and BTHS EBS-treated groups. (**F**) Assembly of OXPHOS complexes in the mouse heart. Heart tissue from BTHS mice treated with solvent or EBS and wild-type mice was analyzed by Blue Native-PAGE as described above. The immunological signals corresponding to OXPHOS supercomplexes (I III IV)_n_ and dimers and monomers of CIV (IV_2_ and IV_1_) and the immunological signals corresponding to ATP synthase oligomers (Vn), dimers (V2), monomers (V1) and the free particle F_1_ are indicated. The same WT SLV and KO SLV images are shown in Appendix Fig. S3G. (**G**) Steady-state protein levels in mouse heart. Mitochondrial protein extracts from BTHS mouse hearts were analyzed by SDS-PAGE and immunoblotting. Protein levels were normalized to GAPDH. Quantification is shown in the right panel. Statistical significance of differences with DMSO-treated mutant cells was estimated using one-way ANOVA followed by Tukey’s multiple-comparisons test. *p < 0.05, ***p < 0.001.

Having established that EBS consistently improves mitochondrial respiration and ATP production across multiple experimental models, we next investigated whether these beneficial effects could result from enhanced accumulation or assembly of oxidative phosphorylation (OXPHOS) complexes. Because CL is required for the stability and organization of respiratory complexes, defects in CL remodeling are expected to impair OXPHOS assembly. Consistent with previous reports (de Taffin de Tilques et al, 2017; Wang et al, 2023), yeast, human and mouse models of BTHS, as well as the *fmc1* yeast mutant, showed reduced steady-state levels of several OXPHOS subunits (Fig. EV4A,B,D,E), together with decreased levels of respiratory supercomplexes and ATP synthase oligomers (Fig. EV4C,F) compared to WT. Surprisingly, despite the marked improvement in mitochondrial respiration and ATP synthesis following EBS treatment (Figs. 1, 2, and 4), neither the abundance of individual OXPHOS subunits nor the assembly of respiratory supercomplexes and ATP synthase oligomers was restored in any of the models examined, including yeast (Fig. EV4A–C), human cells (Fig. EV4D) and mouse heart (Fig. EV4E,F). We next investigated whether EBS could instead enhance mitochondrial function by stimulating mitochondrial biogenesis. To this end, we analyzed the expression of the mitochondrial transcription factor TFAM, a key regulator of mitochondrial DNA replication and transcription. TFAM abundance was increased in the hearts of BTHS mice compared with wild-type animals, consistent with a compensatory response to mitochondrial dysfunction. However, EBS treatment did not further modify TFAM levels (Fig. EV4G), indicating that the improvement in mitochondrial function is unlikely to result from enhanced mitochondrial biogenesis. Together, these findings show that EBS restores mitochondrial function without increasing OXPHOS protein abundance, promoting respiratory complex assembly or stimulating mitochondrial biogenesis, suggesting that its mechanism of action acts through an alternative pathway.

### The beneficial effects of EBS in mitochondrial disease are independent of its antioxidant activity and IMP inhibition

**Figure 5:**
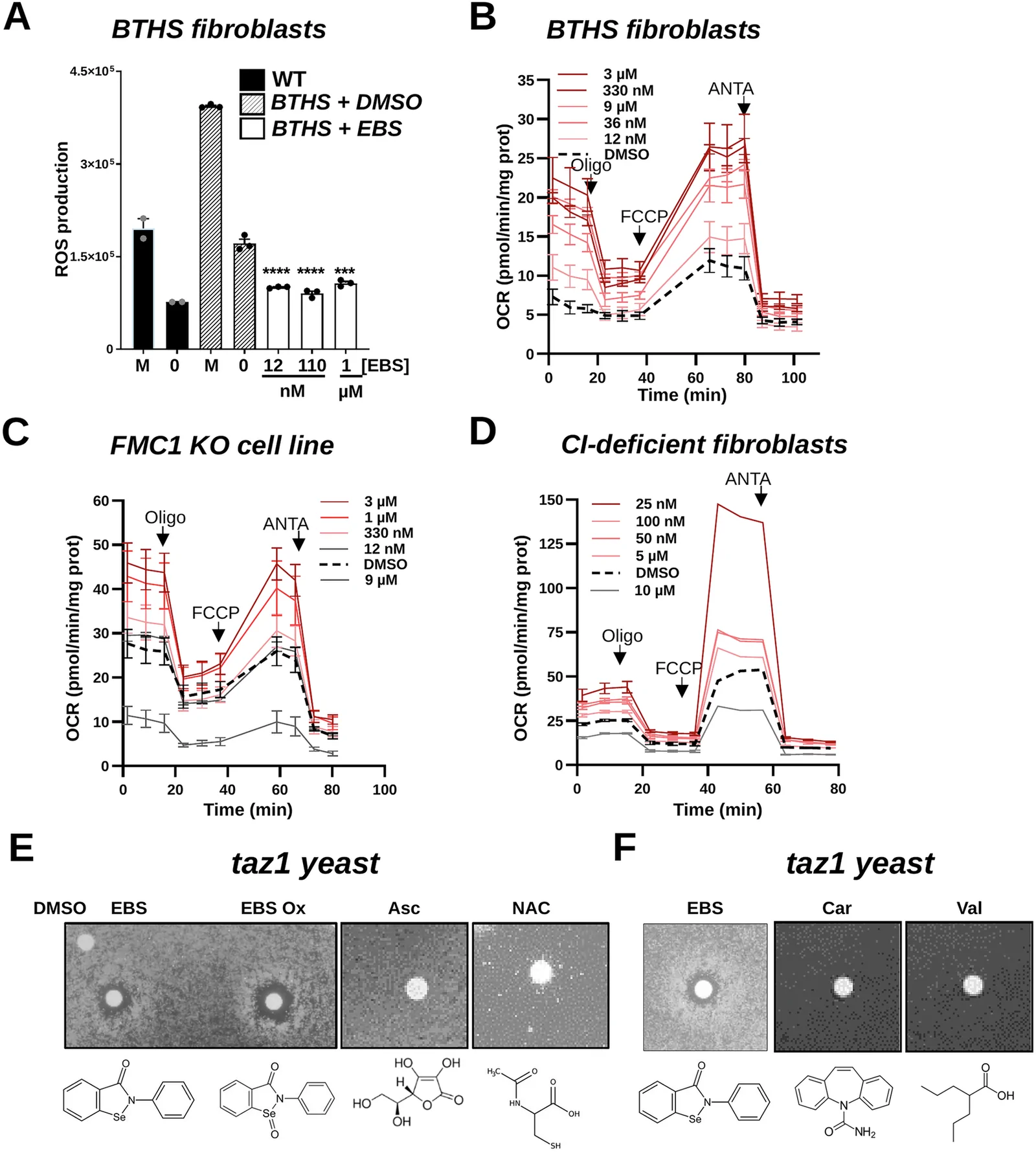
The beneficial effect of EBS in MD models is independent of its antioxidant activity and unrelated to IMP inhibition. (**A**) ROS production in BTHS patient fibroblasts. Cells (1200/well) were seeded in LG medium and treated for 96 h with the indicated concentrations of EBS or DMSO (vehicle control, noted as 0). Reactive oxygen species (ROS) formation was assessed using CellROX Green fluorescence. Menadione (M) was used as a positive control for ROS induction. WT cells are shown as black bars, solvent-treated patient cells as hatched bars, and EBS-treated patient cells as white bars. Data are presented as mean ± SEM from three independent experiments, normalized to cell number. Statistical comparisons were made between EBS-treated and DMSO-treated patient cells. Statistical significance was assessed by one-way ANOVA followed by Tukey’s multiple-comparisons test. ***p < 0.001, ****p < 0.0001. Exact *P* values for all statistical comparisons are provided in Appendix Table S1. (**B**–**D**) Effect of an oxidized form of EBS lacking the antioxidant activity on human MD models. Oxygen consumption rate (OCR) was assessed using the Seahorse mitochondrial stress test in BTHS patient fibroblasts (**B**), HEK FMC1 knockout cells (**C**), and CI-deficient patient cells (**D**), treated for 96 h with increasing concentrations of EBS oxide. OCR was measured under basal conditions, after addition of oligomycin (Oligo, ATP synthase inhibitor), and after uncoupling with FCCP (Uncoupled). Non-mitochondrial respiration was determined after addition of antimycin A (ANTA, complex III inhibitor). For panels (**B**–**D**), increasing EBS oxide concentrations are shown in order of increasing efficacy. Values are means ± SEM from three independent experiments, normalized to total protein content. Statistical significance was calculated between EBS oxide-treated and control conditions. (**E**,** F**) Testing EBS oxide, antioxidants, and IMP inhibitors in the yeast model of BTHS. Taz1 mutant yeast cells were spread as dense layers on solid respiratory medium and exposed to paper disks containing the indicated quantities of each compound. Compounds tested include EBS oxide and two well-known antioxidants (ascorbate (Asc) and *N*-acetylcysteine (NAC)) (**E**), and the IMP inhibitors carbamazepine (Car) and valproate (Val) used in bipolar disorder therapy (**F**). Incubation was carried out for 4–7 days at the indicated temperature (Table 2). The chemical structures of the tested compounds are shown below each corresponding image. Source data are available online for this figure.

Having excluded major effects of EBS on CL abundance, OXPHOS complex assembly and mitochondrial biogenesis, we next investigated whether its beneficial effects could instead be explained by its known pharmacological activities. EBS is currently being evaluated in Phase III and Phase II clinical trials for Ménière’s disease and bipolar disorder, respectively (ClinicalTrials.gov identifiers NCT03325790 and NCT03013400). Its therapeutic efficacy has been attributed either to its antioxidant properties or to its ability to inhibit inositol monophosphatase (IMPase) (Ramli et al, 2022; Singh et al, 2013). Consistent with its reported antioxidant activity, EBS significantly reduced reactive oxygen species (ROS) production in fibroblasts derived from BTHS patients (Fig. 5A). However, an oxidized derivative of EBS, in which the selenium atom is modified and antioxidant activity is abolished (Mouithys-Mickalad Mareque et al, 2004; Sarma and Mugesh, 2008), retained its ability to improve mitochondrial respiration in BTHS fibroblasts (Fig. 5B), CRISPR/Cas9-generated *FMC1* knockout cells (Fig. 5C) and fibroblasts from patients with complex I deficiency (Fig. 5D). Similarly, this compound also restored respiratory growth in the *taz1* yeast model (Fig. 5E). Conversely, the classical antioxidants ascorbate and N-acetylcysteine failed to improve respiratory growth in the BTHS yeast model (Fig. 5E). Together, these findings indicate that although EBS reduces oxidative stress, its beneficial effects on mitochondrial function are independent of its antioxidant activity. We next examined the contribution of IMPase inhibition. Neither carbamazepine nor valproic acid, two clinically used IMPase inhibitors, improved respiratory growth in the BTHS yeast model (Fig. 5F). These results indicate that inhibition of IMPase is also unlikely to account for the beneficial effects of EBS in MD models.

### EBS predominantly localizes to lysosome-associated mTOR signaling complexes

**Figure 6:**
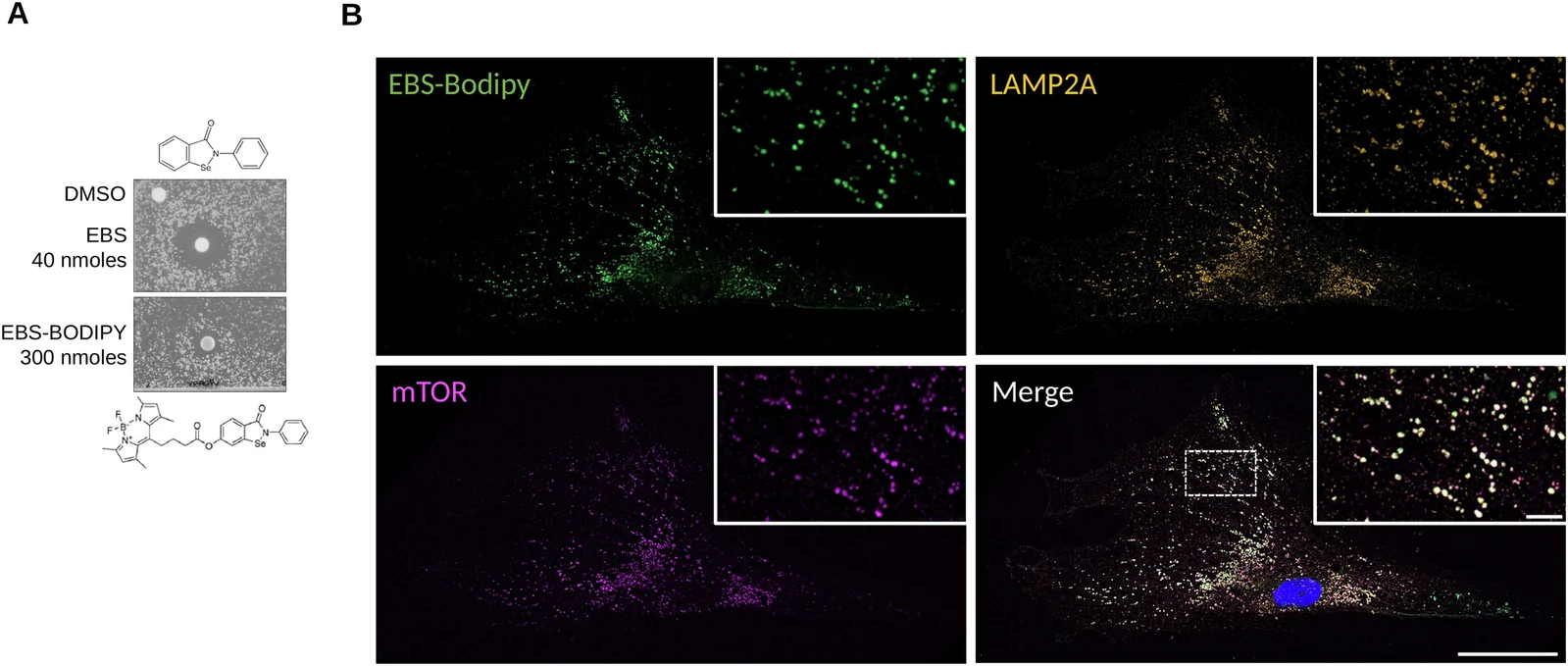
EBS predominantly localizes to lysosome-associated mTOR signaling complexes. (**A**) Modified EBS coupled to BODIPY retains its activity. EBS conjugated to BODIPY was tested for its ability to improve respiratory growth in the BTHS yeast model, using the same strain and method described in Fig. 1. Molecular structures of EBS and EBS-BODIPY are shown above and below the images, respectively. (**B**) Localization of EBS-BODIPY by fluorescence microscopy. BTHS fibroblasts were cultured under the same conditions as in Fig. 3 and treated with 1 µM EBS-BODIPY for 48 h. After fixation, cells were stained with LAMP2A and mTOR. Insets corresponding to 3x enlargement of the selected area shown in the overlay image show that the vesicular localization of EBS-BODIPY (green) colocalizes with the lysosomes (orange) and mTOR (magenta). Scale bars: 50 µm for full images and 5 µm for insets.

Having excluded several established mechanisms that could account for the beneficial effects of EBS, we next sought to identify the intracellular compartment in which EBS exerts its primary activity. Because the subcellular localization of a small molecule can provide important clues to its mechanism of action, we generated a fluorescent EBS derivative by conjugating EBS to the small fluorophore BODIPY (Appendix Fig. S4A), chosen for its minimal impact on cellular uptake and biological activity. Importantly, the EBS-BODIPY conjugate retained the ability to improve respiratory growth in the BTHS yeast model, indicating that fluorophore attachment did not compromise the biological activity of EBS (Fig. 6A). As previously reported (Spangenburg et al, 2011), free BODIPY alone accumulates predominantly in lipid droplets and the Golgi apparatus (Appendix Fig. S4). In contrast, the EBS-BODIPY conjugate displayed a markedly different intracellular distribution, demonstrating that its localization was dictated by the EBS moiety rather than by the fluorophore itself. Microscopy analyses revealed minimal colocalization with a mitochondrial marker (Appendix Fig. S4B) and partial colocalization with an endoplasmic reticulum marker (Appendix Fig. S4C). By contrast, EBS–BODIPY strongly accumulated in lysosome-associated compartment (Appendix Fig. S4D), which constitutes the major cellular platform for nutrient sensing and mTOR signaling. To further define its intracellular localization, we performed additional immunofluorescence experiments with mTOR and the lysosomal marker LAMP2. EBS–BODIPY exhibited extensive colocalization with mTOR, while mTOR itself co-localized with LAMP2, consistent with its expected localization at the lysosomal membrane. EBS–BODIPY also partially co-localized with LAMP2 (Fig. 6B), indicating that EBS is enriched in lysosome-associated mTOR signaling compartments. Together, these findings identify lysosome-associated mTOR signaling compartments as the predominant intracellular site of EBS accumulation and prompted us to investigate the signaling pathways regulated by EBS.

### Affinity proteomics identifies conserved EBS-associated proteins across yeast, human cells and mouse heart

**Figure EV5:**
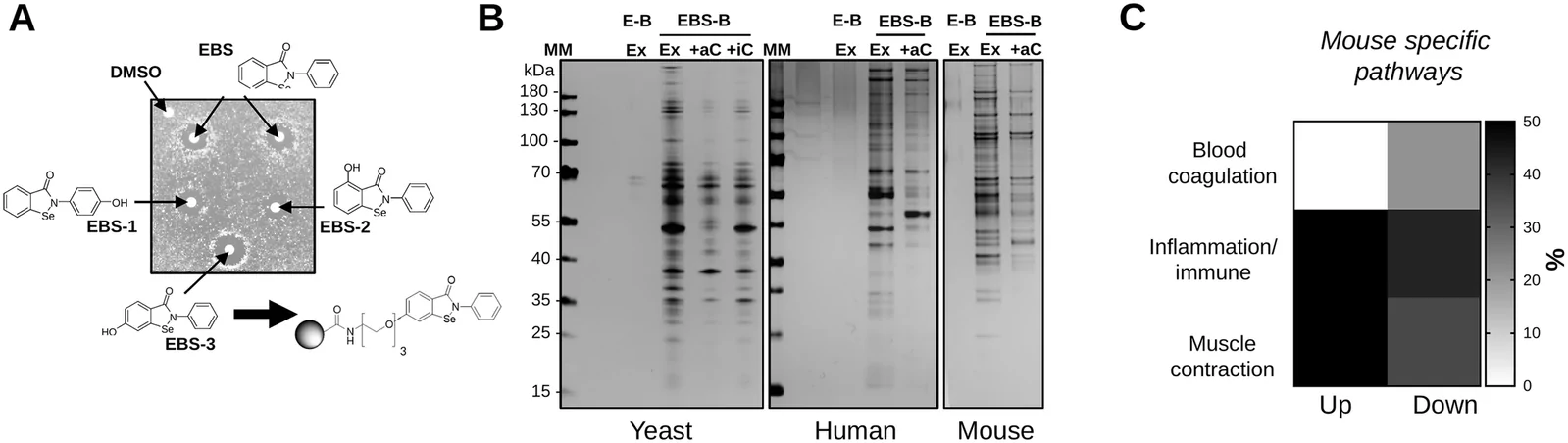
EBS-interacting proteins across disease models and mouse-specific EBS-regulated proteins. (**A**) A modified EBS derivatives retain biological activity. A hydroxy group was introduced at positions 1, 2, or 3 of the EBS molecule, as shown in the molecular structures. These derivatives were tested for their ability to retain their capacity to improve respiratory growth of the BTHS yeast model using the same strain and method as depicted in Fig. 1. The molecular structure of the active EBS derivative with a linker at position 3, covalently bound to magnetic beads, is shown in the bottom right. (**B**) Identification of EBS-binding proteins by affinity chromatography. Protein extracts (Ex) from BTHS yeast (left panel), fibroblasts (middle panel) and murine heart tissue (right panel) were incubated with EBS-functionalized magnetic beads (EBS-B). Empty beads (E-B) served as a negative control. Competitive elution was performed using free EBS (aC) or an inactive oxygenized form of EBS (iC, corresponding to EBS form with an oxygen atom shown in Appendix Fig. S1). A portion of the eluted proteins was resolved by SDS-PAGE and silver-stained, while the remainder was subjected to mass spectrometry analysis to identify specific interactors. (**C**) Mouse-specific EBS-regulated proteins. Proteins significantly deregulated following EBS treatment in the BTHS mouse heart were compared with EBS-regulated proteins identified in the yeast and human models. The panel shows proteins deregulated specifically in the mouse model and not shared with the yeast and human datasets.

**Figure 7:**
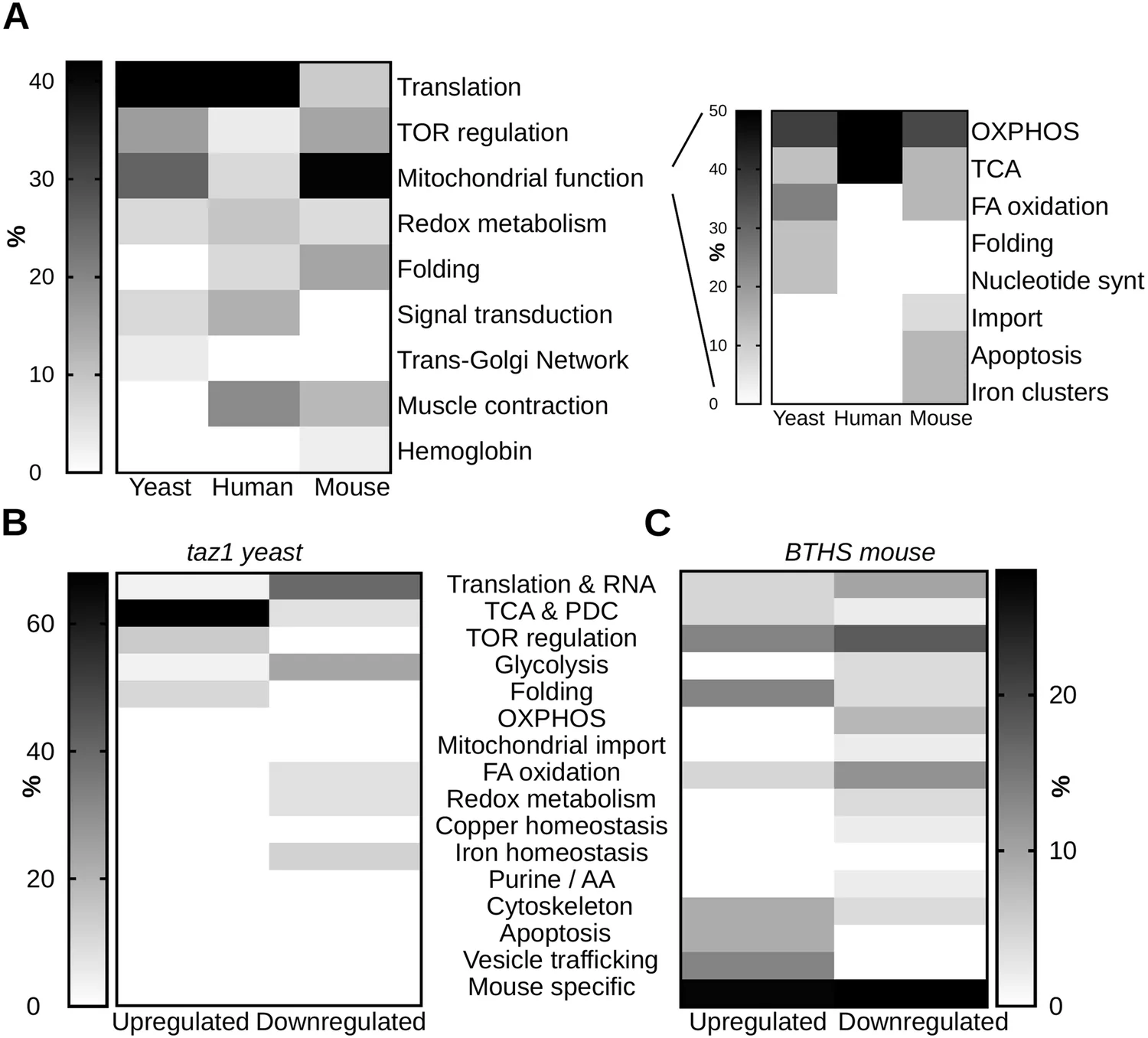
EBS induces proteome remodeling centered on translation, mTOR signaling and mitochondrial metabolism. (**A**) Translation is the most enriched EBS-interacting pathway. Proteins identified as specific EBS binders (filtered using a ratio of EBS-B/E-B > 3 and EBS-B/aC > 3) were categorized into functional pathways and visualized using a heatmap. The 30 most specifically enriched proteins were selected and filtered to retain master proteins with at least two unique peptides and additional selection criteria, including statistical significance and low inter-replicate variability. For each organism, nine major pathways were represented, normalized to 100%. The inset on the right highlights the mitochondrial function pathway. (**B**,** C**) Proteins significantly altered by EBS treatment compared to solvent control were identified in BTHS yeast (**B**) and BTHS mouse heart tissue (**C**), and categorized into functional pathways. Upregulated and downregulated proteins were grouped and visualized using a heatmap representation. For each dataset, the proportion of proteins within each pathway category was calculated as a percentage of the total number of regulated proteins (either upregulated or downregulated), summing to 100%. In Yeast, 25 proteins were significantly downregulated and 28 upregulated, as stated in Table EV5. In the mouse, 50 proteins were significantly downregulated and 23 upregulated following EBS treatment, as stated in Table EV6.

Having identified lysosome-associated mTOR signaling compartments as the principal intracellular site of EBS accumulation, we next sought to identify proteins physically associated with the compound. To this end, we developed EBS-based affinity chromatography matrices capable of capturing intracellular macromolecules interacting with EBS (Guiffant et al, 2007; Tribouillard-Tanvier et al, 2008). To minimize potential steric hindrance between EBS targets and the matrix, a chemical spacer was introduced between the EBS moiety and the magnetic beads. The optimal site for linker attachment was determined through structure-activity relationship analyses, in which several functionalized EBS derivatives were evaluated for their ability to rescue respiratory growth in the BTHS yeast model (Appendix Fig. S1). Among the three derivatives tested, modification of the benzoselenazole ring (EBS-3) best preserved biological activity, whereas modifications at the two alternative positions (EBS-1 and EBS-2) markedly reduced efficacy (Fig. EV5A). The active functionalized EBS molecule was then coupled *via* the selected linker to magnetic beads to generate the affinity chromatography matrices shown in Fig. EV5A (Appendix Fig. S5). Crude protein extracts from the BTHS yeast model, patient-derived human cells and BTHS mouse hearts were incubated with either EBS-coupled beads (EBS-B) or ethanolamine-quenched control beads (E-B). To distinguish specific from nonspecific interactors, competition assays were performed in parallel using an excess of either free active EBS (active competition; aC) or its inactive oxygenated derivative (inactive competition; iC). Proteins specifically interacting with EBS were expected to be displaced by active, but not inactive, EBS (Fig. EV5B). Bound proteins were subsequently identified by mass spectrometry, and high-confidence interactors were selected using stringent enrichment criteria (EBS-B/E-B > 3 and EBS-B/aC >3) (Tables EV2–4). As expected, proteins involved in oxidative stress responses were recovered, providing an internal validation of the affinity purification strategy. More importantly, comparative analysis across the three experimental systems identified a conserved set of EBS-associated proteins involved in cytosolic translation, TOR signaling and central mitochondrial metabolism, including oxidative phosphorylation and the tricarboxylic acid (TCA) cycle (Fig. 7A). In addition, proteins specifically recovered from mammalian samples included factors involved in cardiac contraction and hemoglobin function, reflecting the greater cellular complexity and tissue specialization of the mouse heart. Together, these findings reveal that EBS associates with a conserved molecular network spanning evolutionarily distant models and point to nutrient-sensing, protein translation and mitochondrial metabolism as candidate pathways underlying its broad therapeutic activity.

### EBS remodels the abundance of proteins identified by affinity proteomics

Building on the identification of EBS-associated proteins across evolutionarily distant models, we next investigated whether these candidate targets were functionally modulated following EBS treatment. To this end, we performed comparative quantitative proteomic analyses in BTHS yeast and mouse models treated or not with EBS. Across both models, EBS treatment was associated with a coordinated deregulation of conserved metabolic and signaling pathways under conditions in which mitochondrial function was improved (Figs. 1 and 4). Remarkably, the pathways most consistently affected corresponded to those identified by affinity proteomics (Fig. 7B,C), supporting their functional relevance. In both species, proteins involved in cytosolic translation were predominantly downregulated, representing ~40 and 10% of all downregulated proteins in yeast and mouse, respectively. Glycolytic enzymes were also consistently decreased (~24% in yeast and 5% in mouse). Conversely, proteins associated with mitochondrial metabolism were preferentially upregulated. These included numerous enzymes of the tricarboxylic acid (TCA) cycle, which accounted for nearly 60% of all upregulated proteins in yeast, together with components of the mTOR signaling pathway (~15%). Additional conserved responses included increased abundance of proteins involved in protein folding, fatty acid β-oxidation and redox metabolism (Table EV5 and Table EV6). Importantly, these proteomic signatures were strongly supported by independent transcriptomic analyses (Appendix Fig. S6), which showed highly concordant regulation of the same functional pathways, including reduced cytosolic translation and increased mitochondrial metabolism, TCA cycle activity and mTOR signaling. This multi-omics convergence confirms the functional relevance of the affinity proteomics hits and supports their role as bona fide EBS-responsive networks rather than secondary fluctuations. Although these conserved signatures were observed in both organisms, the mouse heart displayed a broader spectrum of deregulated pathways, likely reflecting the greater physiological complexity of mammalian tissue. In particular, EBS also modulated proteins involved in inflammation, cardiac muscle contraction and blood coagulation, thereby reducing the relative contribution of translation, TCA cycle and mTOR signaling among the total deregulated proteins (Figs. 7C and EV5C). Among the metabolic enzymes affected, pyruvate dehydrogenase phosphatase 1 (PDP1) was strongly induced following EBS treatment, whereas pyruvate dehydrogenase kinase 4 (PDK4) was downregulated by ~2.5-fold (Table EV6). Because PDP1 activates, whereas PDK4 inhibits, the pyruvate dehydrogenase complex (PDC), this reciprocal regulation is predicted to promote the conversion of pyruvate into acetyl-CoA, thereby increasing substrate availability for the TCA cycle. Taken together, these results identify cytosolic translation, mTOR signaling and the TCA cycle as evolutionarily conserved pathways that are both associated with EBS and consistently remodeled following treatment, making them prime candidates to mediate its beneficial effects in MD.

### EBS partially reduces cytosolic protein synthesis in an mTOR-dependent manner

**Figure 8:**
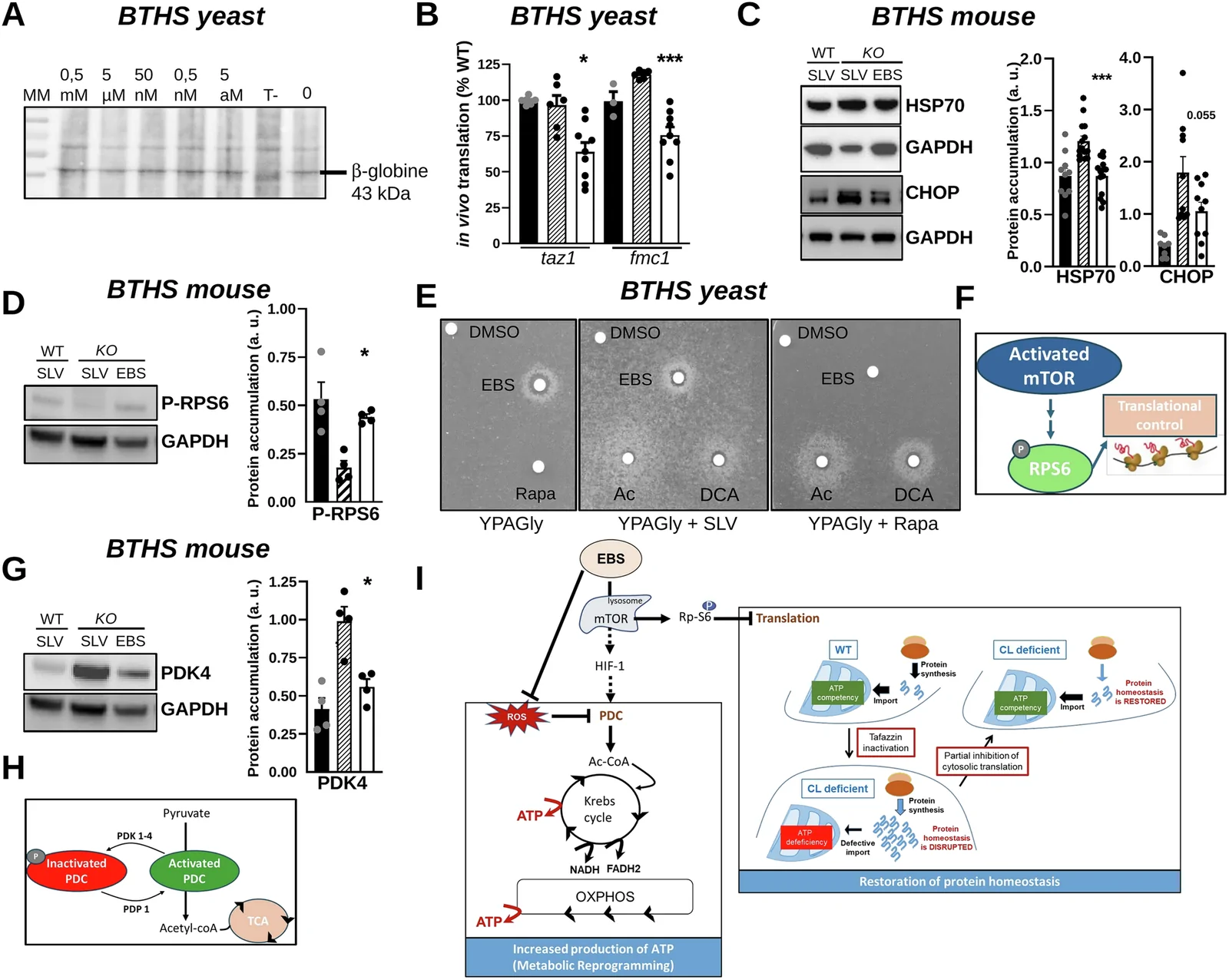
EBS restores mitochondrial function through mTOR-dependent translational control and activation of the PDH pathway. (**A**) In vitro translation. β-globin mRNA was translated using a rabbit reticulocyte lysate (RRL) system in the presence of increasing concentrations of EBS (ranging from 5 aM to 0.5 mM) or solvent alone (0, corresponding to the vehicle used for mouse gavage). A reaction without mRNA (T–) served as a negative control. (**B**) In vivo translation. Total and mitochondrial protein synthesis were assessed in wild-type, *taz1*, and *fmc1* yeast cells treated for 4–5 generations with either DMSO (SLV) or 1 µM EBS under the same conditions as described in Fig. 1. Cells were pulse-labeled for 20 min with [³⁵S]-methionine and [³⁵S]-cysteine, followed by protein extraction and separation by SDS-PAGE (12% polyacrylamide). Gels were dried and analyzed using a PhosphorImager. Cytosolic translation rates were calculated by subtracting mitochondrial signal from total incorporation. Quantification was performed using ImageJ and is expressed as a percentage relative to WT (*n* of 3 with 3 technical replicates). WT cells are represented by black bars, mutant cells treated with solvent by hatched bars, and mutant cells treated with EBS by white bars. (**C**,** D**) Steady-state levels of proteostasis stress markers and phosphorylated RPS6. Total protein extracts from the hearts of WT mice and Taz-KO (BTHS) mice treated with solvent (SLV) or EBS were resolved by SDS-PAGE, transferred to nitrocellulose membranes, and probed with antibodies against CHOP, HSP70, and P-RPS6. Protein levels were normalized to GAPDH. The same GAPDH loading control is shown in panels (**D**,** G**). Quantifications are shown in the right panels. Data were expressed as mean ± SEM (*n* = 10-14 in (**C**) and *n* = 4 in (**D**)). WT mice treated with solvent are shown in black bars, KO mice treated with solvent in hatched bars, and KO mice treated with EBS in white bars. (**E**) Inhibition of mTOR abolishes the effect of EBS, but not of acetate or dichloroacetate, in the yeast BTHS model. The yeast *taz1* mutant was spread as a dense layer on solid respiratory medium, either without additives (left panel), with solvent (SLV, middle panel), or with 500 pM rapamycin (Rapa, right panel). Paper disks were then applied and spotted with 0.025 nmol of Rapa and 30 nmol of EBS, acetate (Ac), or dichloroacetate (DCA). Plates were incubated for 4–7 days at the appropriate temperature (see Table 2). (**F**) Schematic models of the regulation of translation through RPS6 phosphorylation by activated mTOR. (**G**) PDK4 protein levels. PDK4 levels were quantified and normalized to GAPDH as described in panels (**C**, **D**). The same GAPDH loading control is shown in panels (**D**, **G**). Data were expressed as mean ± SEM (*n* = 4). (**H**) Schematic models of PDC activation. (**I**) Schematic representation of identified mechanisms of action of EBS explaining its beneficial effects in MD models. PDC pyruvate dehydrogenase complex, Ac-CoA acetyl-CoA, CL cardiolipin. Statistical comparisons were made between EBS-treated and DMSO-treated patient cells. For (**B**,** C**), statistical significance was assessed by one-way ANOVA followed by Tukey’s multiple-comparisons test. For (**D**–**G**), statistical significance was assessed by one-way ANOVA followed by Dunnett’s multiple-comparisons test. ***p < 0.001, ****p < 0.0001. Exact *P* values for all statistical comparisons are provided in Appendix Table S1. Source data are available online for this figure.

Given the consistent deregulation of the translation pathway across models, we next investigated whether EBS directly affects cytosolic protein synthesis. To this end, we first assessed its impact in a cell-free translation system. EBS had no effect on β-globin translation in vitro (Fig. 8A), indicating that it does not directly interfere with the translational machinery.

We then examined cytosolic protein synthesis in vivo using radiolabelled amino acid incorporation in yeast models of MD. In contrast, EBS significantly reduced cytosolic translation by ~25% in both *taz1* and *fmc1* yeast mutants, under the same conditions in which mitochondrial respiration and ATP synthesis were improved (Fig. 8B). These data indicate that EBS induces a moderate reduction of cytosolic protein synthesis in vivo, likely through indirect mechanisms. Consistently, markers of proteostatic stress were reduced following EBS treatment. In the BTHS mouse model, levels of HSP70 and CHOP were decreased after EBS administration (Fig. 8C), indicating an alleviation of stress responses associated with mitochondrial dysfunction and impaired protein homeostasis. Given the central role of mTOR signaling in the regulation of cytosolic translation, we next investigated whether this pathway could mediate the effects of EBS. Western blot analysis of phospho-ribosomal protein S6 (p-RPS6), a downstream effector of mTOR activity, revealed that p-RPS6 levels were reduced in the hearts of BTHS mice, consistent with repression of mTOR signaling, whereas EBS treatment restored p-RPS6 abundance toward control levels (Fig. 8D). This indicates that EBS reactivates mTOR signaling in conditions of mitochondrial dysfunction. To test whether mTOR activity is required for the beneficial effects of EBS, we used rapamycin in the BTHS yeast model. Rapamycin strongly inhibited growth and prevented full restoration of respiratory growth by EBS (Fig. 8E), indicating that mTOR activity is necessary for the beneficial effects of EBS in this model. Together, these findings support a model in which EBS modulates mTOR signaling, leading to changes in p-RPS6 phosphorylation and a moderate reduction of cytosolic protein synthesis, accompanied by a decrease in proteostatic stress (Fig. 8F).

### EBS promotes pyruvate flux into the TCA cycle through regulation of the PDC inhibitory axis

Building on the identification of conserved metabolic pathways modulated by EBS, we next investigated whether the compound affects the control of mitochondrial carbon entry *via* the pyruvate dehydrogenase complex (PDC), a key metabolic gatekeeper linking glycolysis to the tricarboxylic acid (TCA) cycle. In the BTHS mouse model, expression of pyruvate dehydrogenase kinase 4 (PDK4), a negative regulator of PDC activity, was significantly increased compared with controls, consistent with a metabolic shift away from oxidative metabolism. Strikingly, EBS treatment markedly reduced PDK4 abundance (Fig. 8G), suggesting a reversal of this inhibitory state. Conversely, pyruvate dehydrogenase phosphatase 1 (PDP1), which promotes PDC activation by dephosphorylation, was increased upon EBS treatment (Table EV6). Together, these reciprocal changes are predicted to shift the PDC regulatory balance toward an active state, thereby enhancing the conversion of pyruvate into acetyl-CoA and increasing substrate availability for the TCA cycle (Fig. 8H). This model is consistent with a coordinated metabolic reprogramming induced by EBS, characterized by enhanced mitochondrial oxidative metabolism. Importantly, while mTOR signaling regulates cytosolic translation, the PDC regulatory axis represents an independent metabolic node controlling mitochondrial carbon entry, suggesting that EBS acts on complementary pathways that converge on mitochondrial function.

## Discussion

EBS is a versatile seleno-organic compound currently under investigation for a broad range of therapeutic indications, including Alzheimer’s disease (Xie et al, 2017), amyotrophic lateral sclerosis (ALS) (Capper et al, 2018), acute ischemic stroke (Yamaguchi et al, 1998), schizophrenia (Cabungcal et al, 2014), Parkinson’s disease (Moussaoui et al, 2000), and diabetes (Polianskyte-Prause et al, 2022). It has also shown antimicrobial activity against pathogens such as *Staphylococcus aureus*, *Bacillus anthracis* (Gustafsson et al, 2016), *Escherichia coli* (Zou et al, 2017), *Trypanosoma brucei* (Lu et al, 2013), fungi (Thangamani et al, 2017), and several viruses, including SARS-CoV-2 (Jin et al, 2020), HIV (Thenin-Houssier et al, 2016), Influenza A (Belvisi et al, 2000), and Zika virus (Simanjuntak et al, 2018). Furthermore, EBS has been proposed as an anticancer agent (Feng et al, 2020; Kim et al, 2019; Santofimia-Castano et al, 2018). EBS is currently undergoing Phase III clinical trials for bipolar disorder (Fenn et al, 2020; Sharpley et al, 2020), and very recently, clinical trials for Ménière’s disease were successfully completed by the company Sound Pharmaceuticals (Kil et al, 2022). These ongoing and completed studies underscore EBS’s favorable safety profile, good bioavailability, and its ability to cross the blood–brain barrier (Santi et al, 2021; Takasago et al, 1997).

Our study is the first to demonstrate robust efficacy of EBS across multiple MD models (yeast, human cells, and mice) with impairments in CL, mitochondrial OXPHOS complexes, mtDNA maintenance, and translation. The variability in EBS efficacy and toxicity across models likely reflects differences in the underlying mitochondrial defects and cellular context. In particular, residual respiratory capacity and metabolic state may influence both the magnitude of rescue and the therapeutic window. Importantly, the consistent beneficial effects of EBS across genetically and physiologically diverse systems suggest that its mechanism of action is not restricted to a single disease-specific defect, but rather targets conserved cellular processes relevant to mitochondrial dysfunction.

A major strength of this work lies in the use of evolutionarily distant models, which allowed the identification of conserved cellular responses to EBS. Across systems, one of the most consistent signatures was modulation of cytosolic translation. Proteomic and functional analyses revealed a reduction in components of the cytosolic translation machinery in yeast and mouse models following EBS treatment. This observation is consistent with our previous findings showing that partial pharmacological inhibition of translation with cycloheximide improves mitochondrial phenotypes in Barth syndrome models (de Taffin de Tilques et al, 2018). Although EBS is not a classical translation inhibitor, it has been reported to interfere with protein synthesis in bacteria (Thangamani et al, 2015), and ribosomal proteins have been identified among its potential interactors (Chen et al, 2018). In vivo, EBS induced a moderate (25%) reduction in cytosolic translation in yeast models of MD, without affecting translation in a cell-free system, indicating an indirect mechanism. This reduction was accompanied by decreased expression of proteostatic stress markers such as HSP70 and CHOP in BTHS mouse hearts, consistent with improved protein homeostasis. Given that CL deficiency impairs mitochondrial protein import and leads to cytosolic proteotoxic stress (Gebert et al, 2009; Gebert et al, 2011; Jiang et al, 2000; Lionaki et al, 2023), a moderate reduction in protein synthesis may alleviate this burden and contribute to improved cellular fitness.

In parallel, EBS affected mitochondrial metabolism at the level of pyruvate utilization. We observed increased PDK4 expression in the BTHS mouse model, consistent with inhibition of pyruvate dehydrogenase complex (PDC) activity and reduced carbon entry into the tricarboxylic acid (TCA) cycle. EBS treatment reduced PDK4 abundance and increased PDP1 expression, suggesting a shift toward PDC activation and enhanced metabolic flux into the TCA cycle. This regulatory axis is consistent with previous studies reporting a broad deregulation of TCA cycle enzymes in Barth syndrome, resulting in a general slowdown of mitochondrial metabolism (Li et al, 2019), and with the reported upregulation of PDK4 in BTHS models (Liang et al, 2024). In line with this mechanism, pharmacological modulation of PDK activity using dichloroacetate or acetate, two non-selective PDK inhibitors (Olaniyi and Areloegbe, 2023; Skorja Milic et al, 2021), partially reproduced the beneficial effects of EBS on respiratory growth in yeast models. These findings support the idea that the PDK–PDC axis represents a key regulatory node of metabolic dysfunction in Barth syndrome. Importantly, these metabolic changes occur without restoration of oxidative phosphorylation complex abundance or mitochondrial biogenesis, as TFAM levels, although elevated in BTHS mice, were not further increased by EBS. Thus, EBS improves mitochondrial function primarily by enhancing metabolic substrate flux rather than by correcting structural defects of the respiratory chain.

Respiratory analyses further support this interpretation. In isolated yeast mitochondria supplied with exogenous NADH, which bypasses pyruvate oxidation and the TCA cycle, EBS induced only modest improvements in oxygen consumption. In contrast, in mouse heart mitochondria supplied with physiological substrates feeding into the TCA cycle, EBS restored respiration close to wild-type levels. These results indicate that the primary metabolic effect of EBS is exerted upstream of the respiratory chain under physiological conditions.

Interestingly, modulation of cytosolic translation appears to be linked to mTOR signaling, a central regulator of cellular growth and metabolism. Localization studies revealed that EBS predominantly accumulates in lysosome-associated compartments, where it colocalizes with mTOR, suggesting an interaction with mTOR signaling complexes. Consistently, affinity purification identified mTOR-associated proteins, including MEAK7, further supporting the association of EBS with lysosomal mTOR regulatory hubs. Western blot analyses confirmed that phosphorylation of ribosomal protein S6 (p-RPS6), a canonical downstream effector of mTOR activity and key regulator of cytosolic translation, was reduced in BTHS mouse hearts and restored following EBS treatment. Importantly, pharmacological inhibition of mTOR using rapamycin attenuated the beneficial effects of EBS in yeast models, demonstrating that mTOR activity is required for full phenotypic rescue. Together, these data provide strong evidence that EBS modulates lysosome-associated mTOR signaling, leading to changes in translational control and cellular adaptation to mitochondrial dysfunction.

Beyond this primary axis, mTOR signaling has been reported to regulate broader metabolic programs through downstream effectors such as S6K and HIF1, which have been implicated in the control of metabolic enzymes involved in mitochondrial carbon flux (Karagiota et al, 2023; Panwar et al, 2023; Petr Fojtík et al, 2025). Although not demonstrated in our system, it is tempting to speculate that mTOR-associated signaling may indirectly contribute to the regulation of mitochondrial metabolic pathways observed upon EBS treatment. Altogether, our findings support a model in which EBS engages lysosome-associated mTOR signaling to orchestrate a key adaptive response in MD, characterized by reduced cytosolic translation and improved cellular bioenergetic output. These coordinated effects contribute to enhanced protein homeostasis and metabolic efficiency, thereby counteracting the hallmark deficits of mitochondrial dysfunction (Fig. 8I).

EBS is a pleiotropic compound whose effects are likely dose- and context-dependent. Our affinity and proteomic analyses identified additional EBS-associated proteins involved in redox regulation, protein quality control, iron–sulfur cluster biology, and lipid metabolism, including enzymes such as ACC1 in yeast and LCAT in mouse, as well as proteins involved in fatty acid elongation (Tables EV2–EV5). In Barth syndrome models, EBS did not restore total CL levels, indicating that correction of the primary lipid defect is not required for functional rescue. However, EBS consistently altered CL molecular species toward longer acyl chains in yeast, suggesting an indirect influence on lipid remodeling or metabolism. These lipid changes likely represent secondary adaptations rather than primary drivers of the observed phenotypes, but may contribute to the overall metabolic remodeling induced by EBS.

In conclusion, this study identifies EBS as a promising therapeutic candidate for MD by demonstrating its ability to improve mitochondrial function across multiple preclinical models. Beyond its known antioxidant properties, EBS acts through conserved cellular pathways involving mTOR-associated signaling, regulation of cytosolic translation, and metabolic reprogramming. These coordinated effects improve proteostatic balance and mitochondrial energy production, thereby alleviating key features of mitochondrial dysfunction. The favorable safety profile of EBS supports its further exploration in clinical settings for MD.

## Methods

**Table:** Reagents and tools table

Reagent/resource	Reference or source	Identifier or catalog number
**Experimental models**		
*S.c tRNA*^*Leu*^ model	De Luca et al, 2009	N/A
*S.c shy1* model	Almyre et al, 2025	N/A
*S.c cox2* model	Bonnefoy et al, 2001	N/A
*S.c taz1* model	de Taffin de Tilques et al, 2017	N/A
*S.c sym1* model	Almyre et al, 2025	N/A
*S.c fmc1* model	Lefebvre-Legendre et al, 2001; Schwimmer et al, 2005	N/A
*S.c atp6* model	Rak et al, 2007	N/A
*P.a* NUO-51 model	Sellem et al, 2026	N/A
NDUFV1 mutant fibroblasts	Vincent Procaccio	N/A
BTHS fibroblasts	Agnès Rötig	N/A
BTHS myoblast	Pascale de Lonlay	N/A
*FMC1 KO HEK cells*	CRISP’EDIT, Bordeaux	N/A
NDUFV1 mutant cardiomyocytes	Onnik Agbulut	N/A
BTHS mouse model	Douglas Strathdee	N/A
C57BL/6 N	Jackson Lab	N/A
**Antibodies**		
Yeast Ade13	Benoit Pinson	N/A
Yeast Atp6	Marie-France Giraud	N/A
Yeast Atp1	Jean Velours	N/A
Yeast Cox2	Abcam	110271
Human OXPHOS	Abcam	110411
Human complex II	Abcam	110410
Human ACTIN	Abcam	8226
Human LAMP2A	Abcam	125068
Human mTOR	Proteintech	66888-1-1
Human Troponin T	R&D Systems	MAB1874
anti-mouse IgG2a	Life Technologies	A21131
Mouse OXPHOS	Abcam	110413
Mouse TFAM	Abcam	131607
Mouse Phospho-RPS6	Proteintech	29223
Mouse PDK4	Proteintech	12949
Mouse HSP70	Active motif	61207
Mouse CHOP	Cell Signalling	2895T
Mouse GAPDH	Abcam	8245
Mouse ATP5A	Abcam	14748
Mouse COX5b	Proteintech	11418
anti-mouse AlexaFluor®647	Thermo Fisher	A21236
anti-rabbit AlexaFluor™Plus 555	Thermo Fisher	A32794
GFP	Roche	11814460001
**Oligonucleotides and other sequence-based reagents**		
FMC1-sgRNA2	This study	Method section
FMC-sgRNA4	This study	Method section
F-FMC1-247	This study	Method section
R-FMC1-248	This study	Method section
**Chemicals, enzymes and other reagents**		
Accutase®	Stem Cell Technologies	7920
Acetate	VWR	20103.330
ADP	Sigma	A2754
Antimycin A	Sigma	A8674
Ascorbate	Sigma	A7631
BODIPY	Sigma	790389
Carbamazepine	Sigma	C4024
Carbonyl cyanide m-chlorophenylhydrazone (CCCP)	Sigma	C2759
CellROX™ Green Reagent	Invitrogen	C10444
CellTiter-Glo® kit	Promega	G9242
CHIR99021	Selleckchem	S1263
Complete protease inhibitors	Sigma	11697498001
Dexamethasone	Merck	265005
Dichloroacetate	Sigma	8035410005
DMA	Sigma	271012
DMSO	Sigma	D4540
DMEM high glucose	Gibco	11584516
DMEM low glucose	Gibco	11574446
Ebselen	Sigma	E3520
Glutamate	Sigma	G1251
Glutamine	Biowest	X0551-100
IWP2	Tocris Bioscience	18083912
Kinase-Glo® Max Luminescence Kinase Assay	Promega	V6711
Kolliphor® EL	Sigma	C5135
Malate	Sigma	M1000
Matrigel™, hESC-qualified	Corning	356234
mTeSR™ Plus medium	Thermo Fisher Scientific	17187501
Menadione	Sigma	47775
N-acetyl-L-cysteine	Sigma	A9165
NADH	Sigma	N6005
Oligomycin	Sigma	O4876
PBS	Sigma	P4417
BCA Protein Assay Kit	Thermo Fisher Scientific	10678484
Pluronic™ F-127	Invitrogen	P3000MP
PSC Cryomedium	Life Technologies	A4238801
Pyruvate	Sigma	P2256
Rapamycin	Sigma	553210
Rabbit Reticulocyte Lysate	Promega	L4960
RIPA buffer	Thermo Fisher Scientific	89901
Rotenone	Sigma	R8875
RPMI medium	Thermo Fisher Scientific	10247302
Skeletal Muscle Cell Basal Medium	PromoCell	C-23160
Sendai virus-based kit	Thermo Fisher Scientific	A16517
Sodium pyruvate	Thermo Fisher Scientific	12539059
Succinate	Sigma	S3674
Sulforhodamine B	Sigma	230162
TrypLE™ Select 10X	Thermo Fisher Scientific	A1217701
Triiodothyronine	Merck	642511
Uridine	Sigma	U3003-5G
Valproate	Sigma	P4543
Y-27632	Tocris Bioscience	30001217
Yeastar RNA kit	Zymo	R1002
**Software**		
ImageJ	Schneider CA, Rasband WS, Eliceiri KW. *NIH Image to ImageJ: 25 years of image analysis*. Nature Methods. 2012;9:671–675. https://doi.org/10.1038/nmeth.2089	N/A
3D Manager plugin	ImageJ, NIH, Bethesda, MD, USA	N/A
Thin-Layer Chromatography Visualizer 1® and WinCats VideoScan® software	CAMAG, Muttenz, Switzerland	N/A
Xcalibur software	Thermo Fisher Scientific, Waltham, MA, USA	N/A
**Other**		
The Bioscreen C microcultivation instrument	Oy Growth Curves Ab Ltd, Helsinki, Finland	N/A
Seahorse XFe96 Analyser	Agilent Technologies, Santa Clara, CA, USA	N/A
CLARIOstar®	BMG LABTECH, Ortenberg, Germany	N/A
IonOptix MyoPacer Field Stimulator	IonOptix, Westwood, MA, USA	N/A
treadmill	Rodent Treadmill, Ugo Basile Srl, Gemonio (VA), Italy	N/A
OROBOROS	Oroboros Instruments, Innsbruck, Austria	N/A
Hitachi 7650 transmission electron microscope	Hitachi High-Technologies Corporation, Tokyo, Japan	N/A
iBlot2 Gel Transfer Device	Thermo Fisher Scientific, Waltham, MA, USA	N/A
12% SDS-PAGE gels	Thermo Fisher Scientific	NP0322BOX
Native-PAGE 3-12% Bis-Tris Gels	Thermo Fisher Scientific	BN2011BX10
Automatic TLC Sampler 4	CAMAG, Muttenz, Switzerland	N/A
Ultimate 3000 nanoLC system	Thermo Fisher Scientific, Waltham, MA, USA	N/A
Electrospray an Orbitrap EclipseTM Mass spectrometer	Thermo Fisher Scientific, Waltham, MA, USA	N/A
Orbitrap Fusion™ Lumos™ Tribrid™ Mass Spectrometer	Thermo Fisher Scientific, Waltham, MA, USA	N/A
NanoDrop 2000	Thermo Scientific, Wilmington, DE, USA	N/A
Nextseq 2000	Illumina, San Diego, CA, USA	N/A
scintillation counter	Packard Tri-Carb2100 TR	N/A
fully automated Olympus IX81 microscope	Olympus Corporation, Tokyo, Japan	N/A
fully automated Zeiss Axio Imager M2 microscope	Carl Zeiss Microscopy GmbH, Jena, Germany	N/A

### Fungus

#### Models of mitochondrial diseases

The yeast models of MD used in this study were previously described in (Almyre et al, 2025) and are listed in Table 1. *tRNA*^*Leu*^ is a yeast model of mitochondrial encephalomyopathy —lactic acidosis—stroke-like episode (MELAS) syndrome (De Luca et al, 2009); *shy1 is a* yeast model of Leigh syndrome (Almyre et al, 2025). *cox2* is another yeast model of diseases resulting from defects in the assembly of complex IV (Bonnefoy et al, 2001). *taz1* is a yeast model of Barth syndrome (de Taffin de Tilques et al, 2017). *sym1* is a yeast model of the Navajo hepatocerebral syndrome (Almyre et al, 2025). *fmc1* is a yeast model of cardiomyopathies and neuropathies where a nucleus-encoded protein (Fmc1) involved in the assembly of the F_1_ component of ATP synthase has been deleted (Lefebvre-Legendre et al, 2001; Schwimmer et al, 2005). *atp6 is a* yeast model of NARP and MILS syndromes, where the mitochondrial *ATP6* gene encoding a structural protein of ATP synthase encodes the Atp6-L173R variant equivalent to the human ATP6-L156R mutation (Rak et al, 2007). The *Podospora anserina* complex I disease model strain contains the A357V substitution at the end of the ubiquitin-like domain of the NDUFV1 Podospora homolog, NUO-51. In addition to the *nuo-51*^*A357V*^ mutation (equivalent to the human NDUFV1-A341V mutation), the *aox* and *ndi-1* genes, which encode the alternative oxidase and the main NADH dehydrogenase from the internal membrane, respectively, are also deleted to better mimic the human OXPHOS chain (Sellem et al, 2026).

**Table 1:** Genotypes and origins of yeast strains.

Strain	Nuclear genotype	mtDNA	Plasmid	source
*tRNA*^*Leu*^	*MCC123: MATα, his3–11, ade2–1, leu2–3,112, ura3–1, trp1-D2, can1–100, syn*^*−*^	ρ^+^ *tRNA*^*Leu*^*A30(29)G*	ø	(De Luca et al, 2009)
*cox2*	*D273: MATa lys2 leu2-3,112 ura3-52 his3DHindIII arg8::hisG*	ρ ^+^ *cox2-22*	ø	(Bonnefoy et al, 2001)
*shy1*	*W303: MATa ade2-1 ura3-1 his311, 15 trp1-1 leu2-3,112 can1-100 shy1*^*G137R*^	ρ + intron-less	ø	(Almyre et al, 2025)
*taz1*	*W303: MATa ade2-1 ura3-1 his311, 15 trp1-1 leu2-3,112 can1-100 taz1::TRP1*	ρ +	ø	(de Taffin de Tilques et al, 2017)
*sym1*	*W303: MATa ade2-1 ura3-1 his311, 15 trp1-1 leu2-3,112 can1-100 sym1::kanMX6*	ρ +	ø	(Almyre et al, 2025)
*atp6*	*MR6: MATa ade2-1 his3-11,15 trp1-1 leu2-3,112 ura3-1 CAN1 arg8::HIS3*	ρ + ATP6^L173R^	ø	(Rak et al, 2007)
*fmc1*	*MR6: MATa ade2–1 his3–11,15 trp1–1 leu2–3,112 ura3–1 fmc1::HIS3*	ρ + intron-less	ø	(Lefebvre-Legendre et al, 2001)
*Pa nuo-51*^*A357V*^	*S, mat-, nuo-51*^*A357V*^*::NatR, ∆ndi-1::NatR, ∆aox::HPH*	ρ +	ø	(Sellem et al, 2026)

mtDNA, mitochondrial DNA; ∅, no plasmid.

#### Growth media

YPDA is a rich glucose medium used for growing yeast by fermentation: 1% (w/v) yeast extract, 2% (w/v) bacto peptone, 40 mg/L adenine and 2% (v/v) glucose. YPG and YPE are respectively rich glycerol and ethanol media used for growing yeast by respiration: 1% (w/v) yeast extract, 2% (w/v) bacto peptone, 40 mg/L adenine and 2% (v/v) ethanol (YPE) or glycerol (YPG). Solid media contained 2% (w/v) agar. The *Podospora anserina* mutant is thermosensitive at 32 °C on solid synthetic medium (M2) (http://podospora.i2bc.paris-saclay.fr/) compared to the wild-type. The media and temperatures for growing the different fungus models are indicated in Table 2.

**Table 2:** Mutant-specific growth conditions used to visualize EBS effect.

Strain	Solid medium	Liquid medium	Incubation temperature
*tRNA*^*Leu*^	*YPG*	YPG	28 °C
*cox2*	*YPG*	YPG + 0,1% galactose	36 °C
*shy1*	*YPG*	YPG + 0,1% galactose	28 °C
*taz1*	*YPE*	YPE + 0,2% galactose	36 °C
*sym1*	*YPE*	YPE + 0,2% galactose	36 °C
*atp6*	*YPG*	YPG + 0,2% galactose	28 °C
*fmc1*	*YPG*	YPG + 0,2% galactose	36 °C
*Pa nuo-51*^*A357V*^	*M2*	-	32 °C

EBS, ebselen.

#### Yeast-based drug screening assay

Yeast growth assays were conducted as in our previous report (Almyre et al, 2025). Growth improvement was assessed after several days of incubation at the appropriate temperature (see Table 2).

#### Podospora anserina halo test

The mycelium from cultures of the complex I disease model strain grown for 48 h on M2 plates at 28 °C was collected and dispersed by mixing with M2 liquid medium in a FastPrep apparatus (20 s, speed 4) before spreading on an M2 plate. A 6 mm cellulose disc was then deposited on the plate, and 20 nmol of EBS dissolved in dimethyl sulfoxide (DMSO) that served as a vehicle (VEH) were spotted on the filter; one control filter was also spotted with DMSO only. Within 4 to 6 days of incubation at 32 °C, the molecules diffused in the culture medium and allowed the mycelium to develop further from the disks, as a circle of restored growth around a clear area of inhibited growth, due to EBS toxicity.

#### Automatic monitoring of yeast growth

The Bioscreen C microcultivation instrument was used to monitor the growth of yeast strains in 100 µL of respiratory media (see Table 2) supplemented or not with the drugs at the appropriate temperatures (see Table 2) and under continuous shaking. Cell densities (OD_600 nm_) were automatically recorded every 20 min over a period of 100 h.

#### Oxygen consumption in cells

Oxygen consumption was conducted as in our previous report (Almyre et al, 2025). References are in the reagents and tools table.

#### Oxygen consumption and ATP synthesis in mitochondria

Mitochondria were prepared from yeast grown in rich glycerol (YPG) in the presence of EBS or DMSO to an OD_600_ between 2 and 4 by the enzymatic method described in (Guerin et al, 1979). Oxygen consumption and ATP synthesis on mitochondria was conducted as in our previous report (Almyre et al, 2025) using an OROBOROS oxygraph system. References are in the reagents and tools table.

#### Protein extraction

Protein extraction was conducted as in our previous report (Almyre et al, 2025).

#### Commercial origins of the drugs and chemicals used in individual tests

References are in the reagents and tools table. The synthesis of EBS analogs was carried out using previously published procedures (Sancineto et al, 2015) that had been adapted for the desired compounds by S.A. and M.A.E (Appendix Fig. S1A,B and see section «Compound synthesis» in Appendix Supplementary Information).

### Human cells

#### Human cell culture and assays with EBS

##### Complex I-deficient patient cells

The control and NDUFV1 mutant fibroblasts carrying the c.1162+4 A > C and c.1156 C > T (p.Arg386Cys) NDUFV1 variants were cultured in DMEM high glucose (HG, 4.5 g/L) medium (Pan biotech) supplemented with 10% fetal bovine serum (FBS), 1% glutamine and 50 µg/mL uridine (Sigma) and 100 µg/mL sodium pyruvate (Gibco) at 37 °C with 5% CO_2_ in a humidified atmosphere as described before (Desquiret-Dumas et al, 2012). Prior to EBS treatment, cells were incubated for 48 h in the same medium without uridine and pyruvate. During treatment exposure, cells were cultured in low glucose medium (1 g/L) supplemented with L-glutamine and exposed to different concentrations of EBS (freshly dissolved in DMSO) for 48 h.

##### Barth syndrome cells

Primary dermal fibroblasts from eight unrelated Barth syndrome (BTHS) patients and two from healthy individuals (controls) were cultured like NDUFV1 cells except that the HG DMEM medium was from Gibco. Molecular diagnosis was available for four patients, carrying the following TAZ variants: c.598 G > T, c.356 T > G, c.143delinsGG, and c.583+3 A > G. To test the influence of EBS, patient and control fibroblasts were cultured in low glucose (LG, 1 g/L) DMEM medium and exposed for 96 h to different concentrations of EBS (freshly dissolved in DMSO). Primary myoblasts from one BTHS patient and one healthy donor were obtained from the AP–HP Biological Resource Center (Assistance Publique–Hôpitaux de Paris) and cultured in Skeletal Muscle Cell Basal Medium (PromoCell) at 37 °C in a humidified atmosphere containing 5% CO₂. Cells were treated with increasing concentrations of EBS (dissolved in DMSO) for 120 h under the same culture conditions.

##### FMC1 KO HEK cells

The human FMC1 KO HEK cell line has been constructed by the CRISP’edit, technology platform, which is based in Bordeaux, France. FMC1-sgRNA2: CCGACCCTATCGCGACACCG and FMC-sgRNA4: GTCCTCGAAAAGTGTGCGAC were used. FOR PCR verification, the following oligonucleotides were selected: F-FMC1-247: TCGGTCAACGGACAAGTGAG and R-FMC1-248: TTGGTCCCCGAGTTTATGCC. The FMC1 KO and the control HEK cell lines were cultured like BTHS fibroblasts.

##### Complex I-deficient iPSC cells and the generation of cardiomyocytes

NDUFV1 patient fibroblasts were reprogrammed into IPSCs using a non-integrative method with a Sendai virus-based kit (#A16517, Thermo Fisher Scientific). Two independent patient-derived iPSC clones (clones 17 and 18) and control lines (CW30356 and WTSi004B) were cultured and amplified at 37 °C and 5% CO₂, on Matrigel® (hESC-qualified, Corning) coated dishes, using mTeSR™ Plus medium (Stem Cell Technologies). Morphological analyses were performed using both independent clones (17 and 18), whereas functional analyses (contractility and calcium transients) were performed using clone 17. The cells were passaged in clumps at a 1:30 ratio when they reached approximately 80% confluency, utilizing ReLeSR (Stem Cell Technologies). Cardiac differentiation is based on the GiWi protocol published in 2012 (Lian et al, 2012). Initially, induced pluripotent stem cells (iPSCs) were cultured until they reached 80% confluence. At this point, the cells were detached using Accutase® (Stem Cell Technologies), counted, and seeded at an optimal density. Two days later, cardiac differentiation was initiated by replacing mTeSR™ Plus medium with RPMI medium (Thermo Fisher Scientific) enriched with 2% insulin-free B27 supplement (Thermo Fisher Scientific) containing 6-9 μM CHIR99021 (Selleckchem). This medium was replaced with RPMI supplemented with insulin-free B27 after 24 h. On Day 3 of differentiation, the cells were treated with RPMI containing insulin-free B27 and 5 μM IWP2 (Tocris Bioscience) for 48 h. Following this treatment, the medium was again replaced with RPMI supplemented with insulin-free B27. The culture medium was changed every 2 days, maintaining the cells in RPMI enriched with 2% B27 supplement. At day 12 of differentiation, cardiomyocytes were detached with TrypLE Select 10X (Thermo Fisher Scientific) (incubation at 37 °C for 10 min), suspended in RPMI containing 20% fetal bovine serum (Thermo Fisher Scientific) and 5 μM Y-27632 (Tocris Bioscience), then after centrifugation replated in 50,000 cells/cm^2^ and amplified until day 30 with RPMI enriched with 2% B27 supplement containing 2 μM CHIR99021 (Selleckchem). On Day 30, the cardiomyocytes were detached once more using TrypLE Select 10X, resuspended in RPMI with 20% fetal bovine serum, filtered through a 70-μm cell strainer, and either used for further analysis or frozen in PSC Cryomedium (Life Technologies). After thawing, the cardiomyocytes were cultured in RPMI enriched with 2% B27, 1 μM dexamethasone (Merck), and 100 nM triiodothyronine (Merck) for 7 days to promote maturation prior to experiments.

#### Cell line authentication and mycoplasma testing

All human cell lines used in this study were regularly tested for mycoplasma contamination using standard PCR-based detection methods and were confirmed to be mycoplasma-free throughout the experiments. Cell line identity was verified by the providers. No additional STR profiling was performed for the newly generated CRISPR-edited cell lines, which were derived from authenticated parental HEK cells.

#### Human cell proliferation rate measurement

Proliferation rate of human cells was conducted as in our previous report (Almyre et al, 2025) using the Sulforhodamine B (SRB) staining protocol described in (van Tonder et al, 2015). The average data from three independent experiments are expressed as a percentage relative to the controls. Growth is calculated as [OD (cells + EBS) − OD (Day 0 cells)]/[OD (cells +  DMSO) − OD (Day 0 cells)].

#### Oxygen consumption

Cellular respiration was evaluated using the Seahorse XFe96 Analyser (Agilent, USA) and the Seahorse Mitochondrial Stress Test kit. Cellular oxygen consumption rate (OCR) was analysed using the Seahorse extracellular flux (XF) 96 analyser (Agilent, Lexington, USA). A total of 1500 BTHS fibroblasts, 1000 FMC1 KO cells, 40,000 NDUFV1 mutant cells, and 50,000 cardiomyocytes were seeded on a Seahorse microplate in 80 µL growth medium overnight. On the day of the Seahorse assay, cells were switched to Seahorse XF base medium and maintained in a CO_2_-free incubator at 37 °C for 1 h. For the mitochondrial stress test, the XF base medium was supplemented with 10 mM LG, 1 mM pyruvate and 2 mM glutamine. OCR was continuously monitored at each of the following phases: baseline and following sequential addition of oligomycin (1.5 µM), carbonyl cyanide p-trifluoromethoxyphenyl-hydrazone (2 µM) and a mixture of rotenone/antimycin A (0.5 µM). Data were acquired and analysed using Wave software (Agilent, California, USA).

#### ATP rate

The production of adenosine triphosphate (ATP) by ATP synthase in BTHS fibroblasts was quantified using bioluminescence, with the CellTiter-Glo® kit (Promega). Briefly, the patient fibroblasts were seeded in LG culture medium in opaque-walled 96-well plates at a concentration of 5000 cells per well in the presence of increasing concentrations of EBS or DMSO. Following a 96 h incubation period at 37 °C and 5% CO₂, medium was removed from wells, and LG medium lacking SVF or distinct OXPHOS inhibitors (oligomycin 1.26 mM, rotenone 1 mM, and antimycin A 1 mM) was introduced. Following a 30-min incubation period at 37 °C, a volume of CellTiter-Glo® Reagent was added to the plates, which were then incubated for a further two min on a shaker at room temperature. The ATP produced was subsequently quantified using a luminometer (CLARIOstar®). The percentage of mitochondrial ATP used was then calculated using the following formula: %ATPm/ATPtotal = [mean (ATP) - ATP (cocktail inhibitors)/mean (ATP) × 100].

#### ROS production

The production of reactive oxygen species was quantified using the CellROX™ Green Reagent (Invitrogen™, ThermoScientific™), a fluorescent dye that is specifically designed to bind to and visualize reactive oxygen species in live cells. Briefly, the cells were seeded in 96-well plates at a concentration of 5000 cells per well and cultured for 96 h in LG culture medium in the presence of increasing concentrations of EBS or DMSO. Subsequently, 10 µL of a final 4 mM menadione solution, a potent prooxidant agent, was added to selected wells 2 h prior to measurement at 37 °C, serving as the positive control. Subsequently, the cells were incubated with CellROX™ Green Reagent (Invitrogen™, ThermoScientific™) at a final concentration of 3.6 µM for 45 min at 37 °C, after which they were washed three times with PBS and resuspended in 100 µL of PBS for the fluorescence readout (excitation wavelength at 485 nm and emission wavelength at 520 nm) using CLARIOstar®. Following the quantification of reactive oxygen species, an SRB proliferation test was conducted in order to normalize the data with respect to cell number.

#### Protein extraction

The cell pellets were collected and resuspended in 1 mL of RIPA buffer (Thermo Fisher) containing Complete protease inhibitors (Sigma). This solution was incubated 30 min on ice and then centrifuged at 20,000 × *g* for 10 min at 4 °C. The resulting supernatants were collected, and the protein concentration was measured by using the detergent-compatible Pierce BCA Protein Assay Kit.

#### Morphological analysis of cardiomyocytes derived from NDUFV1 mutant iPSCs

Cardiomyocytes derived from NDUFV1 or control hiPSCs were seeded on a micropatterned surface obtained using PRIMO patterning (Alvéole, France). Cardiomyocytes were cultured on Matrigel-coated micropatterned substrates (20-μm wide) for 14 days in maturation medium consisting of RPMI 1640 enriched with 2% B27, 1 μM dexamethasone, and 100 nM triiodothyronine. Then washed with PBS, fixed with 4% PFA for 5 min and permeabilized in 0.1% Triton prior to immunostaining. Troponin T (dilution 1:1000) primary antibody was incubated overnight at 4 °C. Then, anti-mouse IgG2a (Alexa 488, dilution 1:1000) secondary antibody was incubated for 1 h at room temperature, diluted in PBS containing 2% of bovine serum albumin. Nuclei were counterstained with Hoechst. At least ten cardiomyocytes were imaged from each experiment with an inverted confocal microscope (Zeiss, France). All images were acquired with a Z-step of 0.5-μm (image size 512 × 512, magnification 63×). The morphological analysis was performed using ImageJ and the 3D Manager plugin to measure cardiomyocyte length-to-width ratios and to determine the volumes and perimeter of cardiomyocytes.

##### Generation of cardiac spheroids and measurement of contractility and calcium transient

Cardiac spheroids were formed from the combination of cardiomyocytes and human primary cardiac fibroblasts (NHCF-V, Lonza) as previously described (Hovhannisyan et al, 2024; Vartanian-Grimaldi and Agbulut, 2026). Briefly, appropriate amounts of cardiomyocytes (8500/Spheroid) and fibroblasts (1500/Spheroid) were mixed in RPMI (Thermo Fisher Scientific) containing 20% of fetal bovine serum (Thermo Fisher Scientific) and seeded into the seeding chamber of an agarose 3D Petri Dish® (Microtissues), cast from a silicone mold containing 35 individual microwells. In this system, cells settled by gravity at the bottom of each microwell, leading to the formation of 35 spheroids. Medium was changed the day after and every 2 days until day 7.

At day 7, the medium contained in the seeding chamber of the 3D Petri Dish® was aspirated and replaced with fresh medium supplemented with Fluo-4 calcium indicator (Thermo Fisher Scientific). Fluo-4 was prepared in RPMI supplemented with 2% B27 together with DMSO containing 20% Pluronic™ F-127 (Invitrogen), to obtain a final dye concentration of 21.5 µg/mL. After 1 h incubation, the medium was removed and replaced with fresh medium. To measure the contractility and calcium transient, the 3D Petri Dish® containing the cardiac spheroids was then placed on a microscope equipped with a temperature-controlled chamber maintained at 37 °C. Spheroids were electrically stimulated using field stimulation electrodes and continuously perfused with medium equilibrated at 5% CO₂. The spheroids were first stimulated at 1 Hz and 40 V for 5 min (IonOptix MyoPacer Field Stimulator), then 6 s acquisitions were made with a 10x objective. The fluorescence videos will be used to analyze calcium flux, while the bright-field videos will be used to analyze contractility using custom-developed MATLAB scripts. All acquisitions for functional evaluation were performed at 82 frames per second.

### Mouse model of BTHS

All mouse experiments were designed and reported in accordance with the ARRIVE Essential 10 guidelines.

#### Mouse model of Barth syndrome

All animal procedures were conducted in accordance with the guidelines set forth by the French Ministry of Agriculture and the French Council on Animal Care, as well as the standards set by the University of Bordeaux Animal Care Committee. The relevant approvals from the French Ministry of Agriculture were obtained for the following projects: #29195; #32581; #39610; and #42074. Mice harboring the Taz-KO and Taz-flox alleles and polymerase chain reaction (PCR) genotyping protocols were described previously (Cadalbert et al, 2015; Ren et al, 2019; Wang et al, 2020). These alleles have been backcrossed onto C57BL/6 N for over ten generations. WT mice in the C57BL/6 N background were purchased from Jackson Laboratories. Heterozygous TazΔ/+ females were crossed with WT C57BL/6 N males to generate experimental male TazΔ/Y knockout mice, WT littermates, and heterozygous females. Male mice were used for phenotypic analyses unless otherwise specified. Animals were studied at defined ages ranging from 1 to 4 months (as indicated in each experiment).

#### Housing and husbandry conditions

Mice were housed in a specific and opportunistic pathogen-free (SOPF) facility at the University of Bordeaux animal facility under controlled environmental conditions (22 ± 1 °C, 50–60% humidity) with a 12 h light/12 h dark cycle. Animals were housed in individually ventilated cages (IVC) with ad libitum access to a standard chow diet and water. Cage bedding was changed regularly according to institutional animal care protocols. Mice were group-housed (2–5 animals per cage) unless otherwise required for experimental procedures. All efforts were made to minimize animal suffering and to reduce the number of animals used.

##### Study design

Male Taz-KO mice and wild-type (WT) littermate controls on a C57BL/6 N background were compared across vehicle- and EBS-treated groups. Vehicle-treated WT mice served as controls. The experimental unit was a single mouse.

##### Sample size

Group sizes were typically *n* = 6 mice per group, with exact n values reported in each figure legend. Sample sizes were determined based on prior experience with this model and feasibility constraints related to the low Mendelian yield of Taz-KO animals; no formal a priori sample size calculation was performed. The total number of animals used is reported in the corresponding Results sections.

##### Inclusion and exclusion criteria

Inclusion criteria were predefined and included correct genotyping, completion of the treatment protocol, and successful completion of the relevant assays. No animals or data points were excluded from analysis unless a technical failure occurred; any exclusions are explicitly reported in the figure legends. Exact n values are reported for each analysis.

##### Randomization

Mice were randomly assigned at the cage level to vehicle or EBS treatment, independently of genotype composition within each cage, to account for variable Mendelian ratios. Cage location and order of testing were balanced when feasible to minimize potential confounders.

##### Blinding

Investigators were aware of group allocation during treatment administration but were blinded during outcome assessment and data analysis whenever feasible.

##### Outcome measures

Outcome measures included endurance capacity, mitochondrial oxygen consumption rates, ATP synthesis rates, mitochondrial ultrastructure, and protein expression levels. For hypothesis-testing experiments, primary outcome measures are specified in the corresponding figure legends.

##### Statistical methods

Statistical analyses were performed using appropriate parametric or non-parametric tests as detailed in the statistical methods section. Data distribution and variance were assessed prior to analysis, and adjustments were made when assumptions of the statistical tests were not met.

##### Experimental animals

All experiments were performed using male mice, as Barth syndrome is an X-linked disorder predominantly affecting males. Mice were on a C57BL/6N background, generated from heterozygous females crossed with WT males, and were analyzed at 3 months of age. Animal provenance and regulatory approvals are detailed above. Experimental mice were produced by breeding heterozygous females with a germline deletion of exons 5–10 of the Taz gene with wild-type male C57BL/6 N mice (Jackson Labs, Bar Harbor, ME, USA) to generate wildtype (WT) male (Taz+/Y) and female (Taz+/+) mice, heterozygous female (Taz∆/+) mice, and hemizygous null (Taz∆/Y [Taz-KO]) male mice.

#### EBS administration

Male wild-type (WT) and Taz-KO mice were administered *via* oral gavage twice daily with a solution of 10 mg/kg of EBS or once daily with a solution of 50 mg/kg of EBS, freshly prepared in a solvent constituted with 0.4% DMA (Sigma), 9.6% Kolliphor EL (Sigma) and 90% PBS (Sigma) from weaning (at three weeks of age) to 3 months of age. The mice were randomly assigned to one of three groups, with six mice in each group. The weight of the mice was recorded on a weekly basis, and their behavior was closely monitored. At 3 months of age, a few days after the endurance test, the mice were sacrificed, and the hearts were collected. The distal region was used for electron microscopy, while a portion of the remaining heart was rapidly frozen in liquid nitrogen and preserved at a temperature of −80 °C for future analyses and protein extraction. The remainder was freshly used for mitochondrial preparation.

#### Endurance test

The Exercise capacity was determined using an incremental exercise test on a treadmill (Ugo Basile). The treadmill is equipped with an electrified metal grid at the end of the moving belt, which encourages mice to run rather than rest on the grid. The first two days were allocated to acclimatizing the mice to the apparatus. The mice were trained to run at a pace between 3 and 10 m/min for a period of 12 min, with a fixed slope of 5° and without the use of electric shocks. On day 3, the mice were permitted to rest. On day 4, the mice were tested for their exercise capacity. This involved running for 5 min at a speed between 3 and 8 m/min, followed by 5 min at a speed between 8 and 12 m/min, and finally 5 min at a speed of 12 m/min, with a fixed slope of 5°. The Mice were removed from the treadmill for exhaustion if they received ten shocks or remained on the shock grid for over 5 s. Running time until exhaustion and total distance run were recorded.

#### Oxygen consumption and ATP synthesis in mitochondria

The isolation of heart mitochondria was performed by differential centrifugation, which enabled the obtaining of a mitochondrial fraction with an extremely high degree of purity while preserving the mitochondria’s metabolic and bioenergetic state. The purity and intactness of this isolation protocol has been previously demonstrated in reference (Brandt et al, 2017). Immediately following the sacrifice, the tissues were collected, minced and cleaned with cold PBS. The pieces of heart were then added to the mitochondria isolation buffer (IB: 300 mM sucrose, 20 mM Tris-base, 1 mM EGTA, and 0.25 mg/L bovine serum albumin (BSA), pH of 7.2) and homogenized with a few strokes of a Potter S homogenizer (Sartorius) using a loose Teflon pestle in an ice-cold IB supplemented with trypsin (0.5 g/l). The mitochondria were isolated by differential centrifugation. The first step was a low-speed centrifugation (1000 × *g*, 10 min, 4 °C) in a swing-out rotor. The second step was a high-speed centrifugation (8000×*g*, 10 min, 4 °C for heart mitochondria and 10,000 × *g*, 10 min, 4 °C for liver mitochondria) using a fixed-angle rotor. The resulting pellet of crude mitochondria was then suspended in IB. The protein concentration was measured using the Pierce BCA protein assay kit, in accordance with the instructions provided by the manufacturer. Mitochondria were diluted to 25 µg/mL in respiration buffer (120 mM sucrose, 50 mM KCl, 20 mM Trizma Base, 4 mM KH_2_PO_4_, 2 mM MgCl_2_, 25 mg BSA). Oxygen consumption rates were measured using the oxygraph OROBOROS in the presence of 50 nM rotenone (Sigma), 8.3 mM succinate (Sigma) and 4.2 mM ADP (state 3 respiration), 80 ng/mL Oligomycine and 50 nM CCCP (uncoupled respiration). For ATP synthesis, mitochondria were diluted to 75 µg/mL in respiration buffer and 1 mM PGM (Pyruvate, Glutamate, Malate from Sigma) and 1.25 mM ADP (Sima) was used to measure the rate of ATP synthesis in the absence and presence (50 μg/mL) of oligomycin. Aliquots were taken every 30 s, and the production of ATP, after stopping the reaction with 3.5% (w/v) perchloric acid and 12.5 mM EDTA, was quantified using KinaseGlo Max Luminescence Kinase Assay (Promega) and a Clariostar Plate Reader.

#### Electron microscopy

##### Ultrastructural studies

Samples were fixed with 2.5% glutaraldehyde in 0.1 M phosphate buffer, pH 7.2, at 4 °C for 4 h, and postfixed overnight with 1% osmic acid + 15 mg/ml of potassium ferricyanide in 0.1 M phosphate buffer, pH 7.2, at 0 °C. Samples were then dehydrated in acetone and embedded in epoxy resin (Araldite, Fluka). 80 nm ultrathin sections were contrasted with 2% lead citrate in water for 1 min, and were observed with a Hitachi 7650 transmission electron microscope.

##### Serial block-face imaging

Fixation, staining, and embedding of tissues were performed according to established protocols developed at the National Center for Microscopy and Imaging Research (NCMIR; UC San Diego, San Diego, CA) (Deerinck et al, 2018) for serial block-face scanning electron microscopy of biological tissue specimens. Resin-embedded samples were glued to a pin (Gatan, Abingdon, UK) using silver-filled conductive resin (EMS Conductive Silver Paint, Hatfield, PA, USA) and left overnight to dry at room temperature. Blocks were faced and trimmed with a diamond knife, Trim90 (Diatome, Nidau, Switzerland), to produce a square of about 1 mm^2^. The four sides were then covered with a thin layer of silver-filled conductive resin. The pin was placed into a ZEISS Gemini field emission gun SEM300, equipped with a 3View2XP in situ ultramicrotome (Gatan, Abingdon, UK). Serial images of the block-face were recorded at an accelerating voltage of 1.2 kV. All images were taken with the following scanning settings: Pixel size 5 nm, pixel dwell time 5 µs, slice thickness 50 nm (Z-slice), image size 5000 × 5000. Images were collected in a DM4 format from Back Scattered Electron with a specific BSE Detector (On point - Gatan Abingdon, UK) using the software Digital Micrograph (Gatan, Abingdon, UK). The data were treated and analysed using Amira (https://www.thermofisher.com/fr/fr/home/electron-microscopy/products/software-em-3d-vis/amira-software.html) for image segmentation and 3D reconstruction. Measurement of mitochondria volumes was carried out with the MaterialStatistics menu of Amira software. Movies of each cell were made using Blender software.

#### Protein extraction

The snap-frozen heart was added to 1 mL of RIPA buffer (Thermofisher) containing Complete protease inhibitors (Sigma). The heart was immediately cut into small pieces and homogenized with a few strokes of a Potter S homogenizer (Sartorius). It was then sonicated, before being centrifuged at 20,000×*g* for 20 min at 4 °C. The resulting supernatants were collected, and the protein concentration was measured by using the detergent-compatible Pierce BCA Protein Assay Kit.

### Miscellaneous techniques

#### SDS-PAGE and Western blotting

About 50 μg of proteins were run on 12% SDS-PAGE gels (Laemmli, 1970) and then transferred onto a nitrocellulose membrane using an iBlot2 Gel Transfer Device from Thermo Fisher Scientific and analyzed by western blotting with the following antibodies. The antibodies specific to yeast were against Ade13 (provided by Benoit Pinson, Bordeaux, France) at a dilution of 1/50000, Atp6 (provided by Marie-France Giraud, Bordeaux, France) at a dilution of 1/10,000, Atp1 (provided by Jean Velours, Bordeaux, France) at a dilution of 1/10,000 and Cox2 (purchased from Abcam) at a dilution of 1/500. The human-specific antibodies were against OXPHOS (purchased from Abcam) at a dilution of 1/1000, complex II (purchased from Abcam) at a dilution of 1/250, and ACTIN (purchased from Abcam) at a dilution of 1/1000. The mouse specific antibodies were against OXPHOS (purchased from Abcam) at a dilution of 1/1000, TFAM (purchased from Abcam) at a dilution of 1/1000, Phospho-RPS6 (purchased from Proteintech) at a dilution of 1/500, PDK4 (purchased from Proteintech) at a dilution of 1/500, HSP70 (Active motif, gifted by Muriel Priault, Bordeaux, France) at a dilution of 1/5000, CHOP (Cell Signalling, gifted by Arnaud Mourier, Bordeaux, France) at a dilution of 1/1000 and GAPDH (purchased from Abcam) at a dilution of 1/5000. Peroxidase-labeled secondary antibodies (at a 1/10,000 dilution) and the ECL reagent of Amersham International were used to detect the antibody conjugates.

#### BN-PAGE and Western blotting

BN-PAGE was performed as described in (Schagger and von Jagow, 1991) from mitochondrial samples containing 200 μg of proteins suspended in 100 μL of extraction buffer (30 mM HEPES, pH 6.8, 150 mM potassium acetate, 12% glycerol, 2 mM 6-aminocaproic acid, 1 mM EGTA, 1.5% or 10% digitonin for ATP synthase and respiratory chain complexes, respectively). To prevent protein degradation, one protease inhibitor cocktail tablet per 10 mL and 1 mM PMSF were added to the samples. After 30 min incubation on ice, the extracts were cleared by centrifugation at 21,950×g, for 30 min at 4 °C and supplemented with 4.5 μL loading dye (5% Serva Blue G-250, 750 mM 6-aminocaproic acid) and run on Native-PAGE 3–12% Bis-Tris Gels (Thermo Fisher Scientific). After transfer onto a PVDF membrane using an iBlot2 Gel Transfer Device, complexes were detected using polyclonal antibodies against yeast Atp1 (provided by Marie-France Giraud, Bordeaux, France) at a dilution of 1/10,000, yeast Cox2, mouse ATP5A (purchased from Abcam) at a dilution of 1/1000 or mouse COX5b (purchased from Proteintech) at a dilution of 1/750.

#### Lipidomic analyses

##### Mouse heart mitochondria and cultured human cells

CL and MLCL content were measured by High-Performance Thin-Layer Chromatography (HPTLC)-densitometry as described (Pinault et al, 2020). Lipids were extracted following the protocol of Bligh and Dyer (Bligh and Dyer, 1959) from either 100 µg of mouse heart mitochondrial proteins or 5–10^6^ cultured cells. Lipid extracts were then separated by HPTLC-densitometry as previously described (Peyta et al, 2015). In order to quantify CL and MLCL, standards and samples were loaded on silica plates using Automatic TLC Sampler 4 (ATS4, CAMAG, Muttenz, Switzerland). After migration, the plates were immersed in the following solution (10% w/v) copper sulfate in 8% (v/v) phosphoric acid solution and then heated for 15 min at 160 °C for mitochondrial samples or 154 °C for cultured cell samples to visualize phospholipids. Quantification was performed using Thin-Layer Chromatography Visualizer 1® and WinCats VideoScan® software (CAMAG, Muttenz, Switzerland).

##### Yeast mitochondria

*Lipid extraction*. Freeze-dried pellets were transferred to a glass tube and resuspended in 4 mL of boiling ethanol for 5 min to prevent lipid degradation, and lipids were extracted according to (Simionato et al, 2013) by the addition of 2 mL methanol and 8 mL chloroform at room temperature. The mixture was then saturated with nitrogen and incubated for 1 h at room temperature. After filtration through quartz wool, cell remains were rinsed with 3 mL chloroform/methanol 2:1, v/v and 5 mL of NaCl 1% were then added to the filtrate to initiate biphase formation. The lower organic phase corresponding to the lipid extract was collected, dried at 40 °C under nitrogen flux and stored at −20 °C.

*Total fatty acid identification and quantification*. Total glycerolipids were quantified from their fatty acids: in an aliquot fraction of the lipid extract, a known quantity of 15:0 was added, and the fatty acids present were transformed as methyl esters (FAME) by a 1-h incubation in 3 mL 2.5% H2SO4 in pure methanol at 100 °C (Jouhet et al, 2003). The reaction was stopped by the addition of 3 mL water and 3 mL hexane. The hexane phase was analyzed by gas chromatography-mass spectrometry-flame ionization detector (GC-MS-FID) (Agilent) on a BPX70 (SGE) column (0.25 mm ID × 0.25 µm film thickness  × 60 m Length). FAMEs were identified by comparison of their retention times with those of standards (Sigma) and of their mass spectra with the NIST database and quantified by the surface peak method using 15:0 for calibration.

*Glycerolipid analysis*. Lipid fractions were then analyzed by high-pressure liquid chromatography coupled to tandem mass spectrometry (HPLC-MS/MS) using appropriate standard lipids as described in (Jouhet et al, 2017). The lipid extracts corresponding to 25 nmol of total fatty acids were sampled and resuspended in 100 μL of chloroform/methanol [2/1,v/v] containing 125 pmol of each internal standard (phosphatidylethanolamine (PE) 18:0–18:0 and diacylglycerol (DAG) 18:0–22:6 from Avanti Polar Lipid, and sulfoquinovosyldiacylglycerol (SQDG) 16:0–18:0 extracted from spinach thylakoid (Deme et al, 2014) and hydrogenated (Buseman et al, 2006).

About 20 µL of lipids were then injected and separated by HPLC using an Agilent 1200 HPLC system with a 150 mm×3 mm (length × internal diameter) 50-μm diol column (Macherey-Nagel), at 40 °C. The mobile phases were hexane/isopropanol/water/1 M ammonium acetate, pH 5.3 [625/350/24/1, v/v] (A) and isopropanol/water/1 M ammonium acetate, pH 5.3 [850/149/1, v/v] (B). For elution, the percentage of B was originally set at 0 for 5 min and was increased linearly to 100% during 30 min, then kept at 100% for 15 min. This was followed by a rapid return to 100% A in 5 min, and the column was equilibrated for 20 min with 100% A before the next injection, hence a total runtime of 70 min with a flow rate of the mobile phase at 200 μL/min. The distinct glycerophospholipid classes were eluted sequentially depending on their polar head group. Mass spectrometric analysis was performed on a 6460 triple quadrupole mass spectrometer (Agilent) equipped with a Jet Stream electrospray ion source. The following settings were used: drying gas heater at 260 °C, drying gas flow at 13 L·min^−1^, sheath gas heater at 300 °C, sheath gas flow at 11 L·min^−1^, nebulizer pressure at 25 psi, capillary voltage at ±5000 V and nozzle voltage at ±1000 V. Nitrogen was used as a collision gas. The quadrupoles Q1 and Q3 were operated respectively at the widest and unit resolution. Phosphatidylcholine (PC) analyses were carried out in positive ion mode by scanning for precursors of m/z 184 at a collision energy (CE) of 34, 52, 52, and 52 eV. Phosphatidylethanolamine (PE), phosphatidylinositol (PI), phosphatidylglycerol (PG), phosphatidylserine (PS), and phosphatidic acid (PA) measurements were performed in positive ion mode by scanning for neutral losses of 141, 277, 189, 185, and 115 Da at CEs of 20, 12, 16, 20, and 16 eV, respectively. DAG and triacylglycerol (TAG) species were identified and quantified by multiple reaction monitoring (MRM) as singly charged ions [M + NH4]+ at a CE of 16 and 22 eV, respectively. Cardiolipin (CL) species were quantified by MRM as singly charged ions [M-H]- at a CE of −45 eV. Quantification was done for each lipid species by multiple reaction monitoring (MRM) with the various transitions previously recorded (Ejsing et al, 2009). Mass spectra were processed using the MassHunter Workstation software (Agilent) for the identification and quantification of lipids. Lipid quantities (pmol) were corrected for response differences between internal standards and endogenous lipids and by comparison with a qualified control (QC), thanks to an in-house software developed by the LIPANG platform. QC extracts correspond to a known *Saccharomyces cerevisiae* lipid extract previously qualified and quantified by thin-layer chromatography and gas chromatography coupled to ion flame detection as described in (Jouhet et al, 2017).

#### Proteomic analyses

##### Sample preparation and protein digestion

Protein samples were solubilized in Laemmli buffer and 5 µg of proteins were deposited onto the SDS-PAGE gel. After colloidal blue staining, each lane was cut out from the gel and was subsequently cut in 1 mm × 1 mm gel pieces. Gel pieces were destained in 25 mM ammonium bicarbonate 50% ACN, rinsed twice in ultrapure water and shrunk in ACN for 10 min. After ACN removal, gel pieces were dried at room temperature, covered with the trypsin solution (10 ng/µl in 50 mM NH_4_HCO_3_), rehydrated at 4 °C for 10 min, and finally incubated overnight at 37 °C. Spots were then incubated for 15 min in 50 mM NH_4_HCO_3_ at room temperature with rotary shaking. The supernatant was collected, and an H_2_O/ACN/HCOOH (47.5:47.5:5) extraction solution was added onto gel slices for 15 min. The extraction step was repeated twice. Supernatants were pooled and dried in a vacuum centrifuge. Digests were finally solubilized in 0.1% HCOOH.

##### nLC-MS/MS analysis and label-free quantitative data analysis

Peptide mixture was analyzed on a Ultimate 3000 nanoLC system (Dionex, Amsterdam, The Netherlands) coupled to an Electrospray an Orbitrap EclipseTM Mass spectrometer (Thermo Fisher Scientific, San Jose, CA) for yeast proteomic analyses and an Orbitrap Fusion™ Lumos™ Tribrid™ Mass Spectrometer (Thermo Fisher Scientific, San Jose, CA) for mouse proteomic and affinity chromatography analyses. Ten microliters of peptide digests were loaded onto a 300-µm-inner diameter × 5-mm C_18_ PepMap^TM^ trap column (LC Packings) at a flow rate of 10 µL/min. The peptides were eluted from the trap column onto an analytical 75-mm id × 50-cm C18 Pep-Map column (LC Packings) with a 4–40% linear gradient of solvent B in 91 min (solvent A was 0.1% formic acid and solvent B was 0.1% formic acid in 80% ACN). The separation flow rate was set at 300 nL/min. The mass spectrometer operated in positive ion mode at a 2-kV needle voltage. Data were acquired using Xcalibur software in a data-dependent mode. MS scans (*m/z* 375–1500) were recorded in the Orbitrap at a resolution of R = 120,000 (@ m/z 200) and an AGC target of 4 × 10^5 ^ions collected within 50 ms. Dynamic exclusion was set to 60 s, and top speed fragmentation in HCD mode was performed over a 3 s cycle. MS/MS scans were collected in the Orbitrap with a resolution of 30,000 and a maximum fill time of 54 ms. Only +2 to +6 charged ions were selected for fragmentation. Other settings were as follows: no sheath nor auxiliary gas flow, heated capillary temperature, 275 °C; normalized HCD collision energy of 28%, isolation width of 1.6 m/z, AGC target of 5 ×  10^4^ and normalized AGC target of 100%. Monoisotopic precursor selection (MIPS) was set to Peptide, and an intensity threshold was set to 2.5 × 10^4^.

##### Database search and results processing

Data were searched by SEQUEST through Proteome Discoverer 2.5 (Thermo Fisher Scientific Inc.) against a *Saccharomyces cerevisiae* reference Proteome Set (Uniprot 2022-01; 5994 entries) or a *Mus musculus* reference Proteome Set (Uniprot 2022-01; 21,945 entries). Spectra from peptides higher than 5000 Da or lower than 350 Da were rejected. The precursor detector node was included. Search parameters were as follows: mass accuracy of the monoisotopic peptide precursor and peptide fragments was set to 10 ppm and 0.02 Da, respectively. Only b- and y-ions were considered for mass calculation. Sequest HT was used as the search algorithm: Oxidation of methionines (+16 Da), methionine loss (−131 Da), methionine loss with acetylation (−89 Da) and protein N-terminal acetylation (+42 Da) were considered as variable modifications, while carbamidomethylation of cysteines (+57 Da) was considered as a fixed modification. Two missed trypsin cleavages were allowed. Peptide validation was performed using the Percolator algorithm (Kall et al, 2007) and only “high confidence” peptides were retained corresponding to a 1% false positive rate at the peptide level. Peaks were detected and integrated using the Minora algorithm embedded in Proteome Discoverer.

For proteomic analyses: Normalization was performed based on the total *Saccharomyces cerevisiae* or total *Mus musculus* peptide amount. Protein ratios were calculated as the median of all possible pairwise peptide ratios. A *t*-test was calculated based on the background population of peptides or proteins and was adjusted using the Benjamini–Hochberg correction. Quantitative data were considered for proteins quantified by a minimum of two peptides and a statistical *p* value lower than 0.05.

For affinity chromatography analyses: There was no normalization. An ANOVA test was calculated based on individual proteins and was adjusted using the Benjamini–Hochberg correction. Quantitative data were considered for proteins quantified by a minimum of two peptides and a statistical *p* value lower than 0.05.

#### Affinity chromatography

Affinity chromatography was performed as described in (Tribouillard-Tanvier et al, 2008). The homogenization buffer for yeast cell extracts was 25 mM Tris (pH 7.4), 100 mM NaCl, 0.2% Triton X-100, with a protease inhibitor cocktail. The homogenization buffer for mammalian cells or mouse heart extracts was 50 mM Tris-HCl, pH 7.5, 2 mM EDTA, 0.1% Triton X-100, with cocktail protease inhibitors. The bead buffer was 50 mM Tris (pH 7.4), 5 mM NaF, 250 mM NaCl, 5 mM EDTA, 5 mM EGTA, 0.1% Nonidet P-40, and protease inhibitor cocktail. Protein extracts: 50 µL of yeast protein extracts, or 150 µg of mouse heart or BTHS myoblast cell protein extract, were added to 50 µL of beads. The bound proteins were analyzed by SDS-PAGE, silver staining and global proteomics. The synthesis of EBS-coupled beads was carried out by S.A. and M.A.E (Appendix Fig. S5 and see section «Compound synthesis» in Appendix Supplementary Information).

#### Transcriptomic

##### RNA extraction

Isolation of total RNA from each yeast pellet was performed with Yeastar RNA kit (R1002, Zymo) following the supplier's procedure. The RNA concentration was measured by spectrophotometry (NanoDrop 2000, Thermo Fisher), and RNA quality was checked with capillary gel electrophoresis (LabChip GX touch HT, PerKinElmer) using the RNA chip DNA 5K/RNA CZE.

##### RNA-seq libraries

cDNA libraries were synthesized using 500 ng of RNA from each sample with the Illumina Stranded mRNA Prep kit according to the supplier's instructions. Briefly, the first step consisted of capturing mRNAs with magnetic beads targeting their poly-A tail. Then, mRNAs were fragmented, reverse transcribed into double-stranded cDNA and 3’ adenylated adapters and anchors were ligated to both ends. Finally, the cDNA libraries were amplified by PCR for 12 cycles and the quality was checked with the Xmark chip on the Lab Chip GX touch HT (Perkin Elmer). After quantification of individual libraries by qPCR using the NEBNext library quant kit (New England BioLabs), all the samples were pooled in an equimolar manner. The quality of the library pool was checked by capillary gel electrophoresis (Lab Chip GX touch HT, Perkin Elmer), and it showed an average library size of ~350 bp and the absence of adapter contamination. The concentration of the library pool was determined using qPCR with the NEBNext Library Quant Kit for Illumina (contains primers which target the P5 and P7 Illumina adaptor sequences) before sequencing. Sequencing was performed on a Nextseq 2000 (Illumina) at a concentration of 750pM in the paired-end mode (2 × 100 bp) with a minimum of 30 million reads per sample in the PGTB facility (INRAE - Pierroton).

##### Bioinformatic analyses

Raw data were applied to our in-house bioinformatic pipeline. Data quality assessment was performed by FastQC and MultiQC (Ewels et al, 2016), read trimming with Cutadapt (Martin, 2011) and Fastp (Chen et al, 2018). Next, STAR (Dobin et al, 2013) was used to map the trimmed reads to the reference genome Saccharomyces_cerevisiae.R64-1-1 and to quantify the mapped reads, and the alignment performance was analyzed by Qualimap (Garcia-Alcalde et al, 2012). R package DESeq2 (Love et al, 2014) was used to normalize and perform differential expression analysis on the quantified reads, to identify differentially expressed genes with FDR-adjusted *p* value <0.05 and absolute value of fold change >1.5 and to prepare the data for functional enrichment analysis. Gene set enrichment analysis identified enriched gene sets from the normalized count data, and clusterProfiler (Yu et al, 2012) was used to perform functional enrichment against the GO databases.

#### In vitro and in vivo translation assays

The in vitro translation assay was conducted using the Rabbit Reticulocyte Lysate (RRL) kit (Promega), in accordance with the instructions provided by the manufacturer. A β Globin-eGFP mRNA construct was used to test the translation efficiency under EBS exposure, with an expected protein size of 43 kDa. EBS was tested at various concentrations (0.5 mM to 5 aM). The in vitro translation products were detected by Western blot analysis, using an anti-GFP antibody (Roche)*.*

In vivo translation was performed as described in (de Taffin de Tilques et al, 2018). The indicated strains were grown to early exponential phase (OD/mL of 2) in 20 mL of the appropriate media at the appropriate temperature (see Table 2). After the labeling reactions, total protein extracts were prepared and quantified using the Lowry method. The proteins were separated by SDS-PAGE on a 12% polyacrylamide gel, transferred onto a nitrocellulose membrane and analyzed with a PhosphorImager. Radioactivity was also counted using a scintillation counter (Packard Tri-Carb2100 TR).

### Immunofluorescence

HEK cells and human fibroblasts were seeded at a density of ~50,000 cells per well in a 12-well plate containing 12-mm 1.5H coverslips in high glucose medium and incubated at 37 °C under 5% CO_2_. After 24 h, the medium was removed, and cells were placed in low glucose medium and incubated at 37 °C under 5% CO_2_ for 24 h to 48 h (fibroblasts). Cells were then treated with 300 nM (HEK) or 1 mM (fibroblasts) of either EBS-Bodipy or Bodipy (790389, Sigma) alone for another 48 h and fixed. HEK cells were fixed with 4% paraformaldehyde (PFA)/0.1% glutaraldehyde (GA) in PBS for 20 min. Before fixation, cells were labeled for 30 min with either MitoTracker^TM^ Red CMXRos, LysoTracker^TM^ Red DND-99, or ER-Tracker^TM^ Red as described by the manufacturer (Fisher Scientific). Fibroblasts were fixed with 4% PFA in PBS for 20 min. Following fixation, cells were quickly washed three times in PBS and post-extracted for 10 min in PBS with 0.1% Triton X-100. PFA and GA free radicals in excess were reduced with 1 mg/ml of NaBH4 in PBS for 10 min, then cells were incubated in the blocking buffer (BF; PBS with 2% BSA) for 15 min. Next, coverslips were incubated with primary antibodies (Rabbit anti-LAMP2A; 1:200, Abcam; Mouse anti-mTOR, 1:100, Proteintech) diluted in the BF for 1 h at 37 °C. Coverslips were then washed in BF three times for 5 min and subsequently incubated for 45 min at 37 °C with secondary antibodies (Donkey anti-rabbit AlexaFluor™Plus 555; 1/200, #A32794, Thermo Fisher; Goat anti-mouse AlexaFluor®647; 1/200, #A21236, Thermo Fisher) diluted in BF in a dark chamber. Finally, coverslips were washed in PBS three times for 5 min, and mounted on a glass slide with Fluoroshield® (Sigma) mounting medium.

### Optical microscopy

HEK cells were imaged with a fully automated Olympus IX81 microscope (Olympus, Tokyo, Japan) equipped with a pE-4000 light source (CoolLED) 40X/1.3NA UPlanFL N, 60X/1.45NA PlanApo and 100×/1.4NA UplanSApo oil immersion objectives (Olympus, Tokyo, Japan), an ORCA Fusion camera (Hamamatsu, Japan) under the control of the CellSENS software (Olympus, Tokyo, Japan). Z-stacks were deconvolved using the ImageJ (Rasband, W.S., ImageJ, US National Institutes of Health, Bethesda, Maryland, USA, https://imagej.net/ij/, 1997-2018), DeconvolutionLab plugin (BIG, EPFL).

Fibroblasts were imaged with a fully automated Zeiss Axio Imager M2 microscope equipped with a Viluma 7 light source, Plan-Neofluar40X/1.3NA, Plan-Apochromat100X/1.4NA oil immersion objectives, a Axiocam 712 mono R2 camera under the control of the ZEN 3.2 sofware (Carl Zeiss, Iena, Germany). Z stacks were deconvolved using ZEN 3.2 software using the fast iterative Gold-Meinel algorithm. Images presented are colored selected planes of representative cells.

#### Statistical tests

Statistical analyses were performed using GraphPad Prism. Data were presented as mean ± SEM. The number of biological replicates (*n*) is indicated in the corresponding figure legends. For animal experiments, mice were randomly assigned to the experimental groups. Investigators were blinded to treatment and/or genotype during data acquisition and analysis whenever applicable. Statistical tests were selected according to the experimental design and are indicated in the corresponding figure legends. For multiple comparisons, one-way ANOVA followed by the appropriate multiple-comparison test (Dunnett’s, Tukey’s, or Sidak’s test) was used, as indicated. For comparisons between two independent groups, the Mann–Whitney test was used where appropriate. A two-sided *P* < 0.05 was considered statistically significant. Significance thresholds : *p < 0.05; **p < 0.01; ***p < 0.001, ****p < 0.0001. Exact *P* values are provided in Appendix Table S1.

## Supplementary information

Table EV1

Table EV2

Table EV3

Table EV4

Table EV5

Table EV6

Appendix

Peer Review File

Movie EV1

Movie EV2

Movie EV3

Movie EV4

Movie EV5

Source data Fig. 1

Source data Fig. 2

Source data Fig. 3

Source data Fig. 4

Source data Fig. 5

Source data Fig. 8

Expanded View Figures

## Data Availability

All values underlying the figures and reported means are provided in the supporting data values file. Transcriptomic data have been deposited in the Annotare from EMBL under accession number E-MTAB-16519 (https://www.ebi.ac.uk/biostudies/ArrayExpress/studies/E-MTAB-16519?key=6c22f01f-5958-4d72-bb96-326f67f201eb). Proteomic data have been deposited in the ProteomeXchange Consortium via the PRIDE repository under accession number PXD072219 (username: reviewer_pxd072219@ebi.ac.uk; password: ynJJwfXa6tA0). Additional data are available from the corresponding author upon reasonable request. The source data of this paper are collected in the following database record: biostudies:S-SCDT-10_1038-S44321-026-00518-1.
