# Supplementary material for: Ebselen restores mitochondrial function through metabolic rewiring in Barth syndrome and related mitochondrial diseases
Source: EMBO Mol Med. 2026 Oct 5;18(11):4227–59. doi: 10.1038/s44321-026-00518-1 (PMC13748542; doi:10.1038/s44321-026-00518-1)
Supplement: Supplementary file 1 [file 44321_2026_518_MOESM20_ESM.pdf]

Expanded View Figures

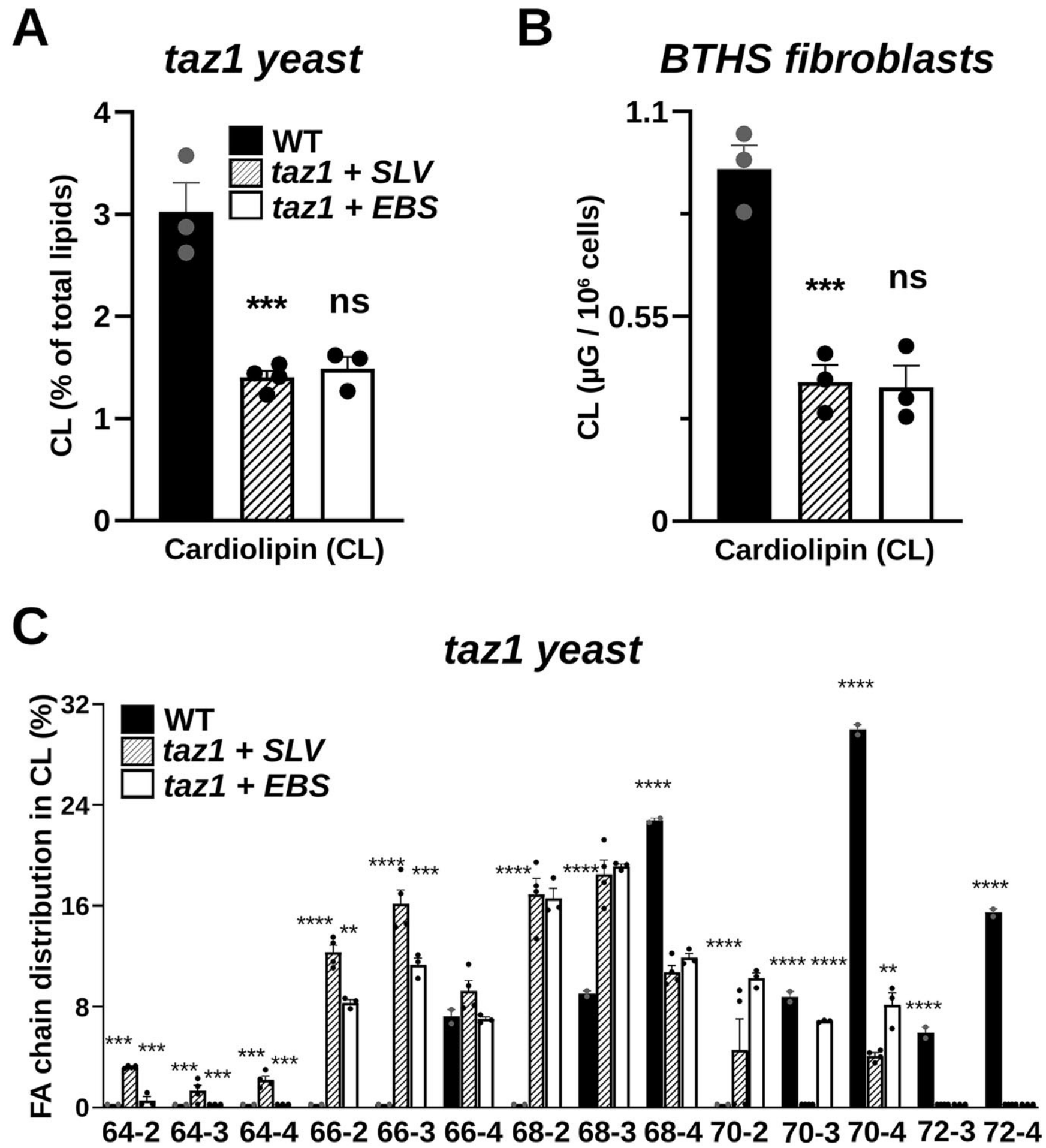

Figure EV1. EBS does not restore cardiolipin abundance but alters cardiolipin molecular composition.

(**A**) Representative glycerolipid compositions of mitochondrial membranes from BTHS yeast model, treated with solvent (DMSO) or EBS. The distribution of CL is given in percent of total lipid mass. (**B**) Representative distribution of CL in control and BTHS fibroblasts treated with solvent (DMSO) or EBS. Control fibroblasts treated with solvent are shown in the black bar, solvent-treated BTHS fibroblasts as hatched, and EBS-treated BTHS fibroblasts as white bars. (**C**) Fatty acid chain distribution of cardiolipin in the BTHS yeast model, solvent or EBS-treated. The first number indicates the number of carbons, and the second number refers to the number of double bonds (unsaturations). Values are expressed in percentages of the total fatty acid content. WT cells are shown in the black bar, mutant cells treated with solvent are shown in the hatched bar and mutant cells treated with EBS are shown in the white bar. For (**A–C**), the reported values are means calculated from 3 independent experiments ±SEM. Statistical significance of differences with DMSO-treated mutant cells was estimated using one-way ANOVA followed by Dunnett's multiple-comparisons test. $^{**}p < 0.01$, $^{***}p < 0.001$, $^{****}p < 0.0001$.

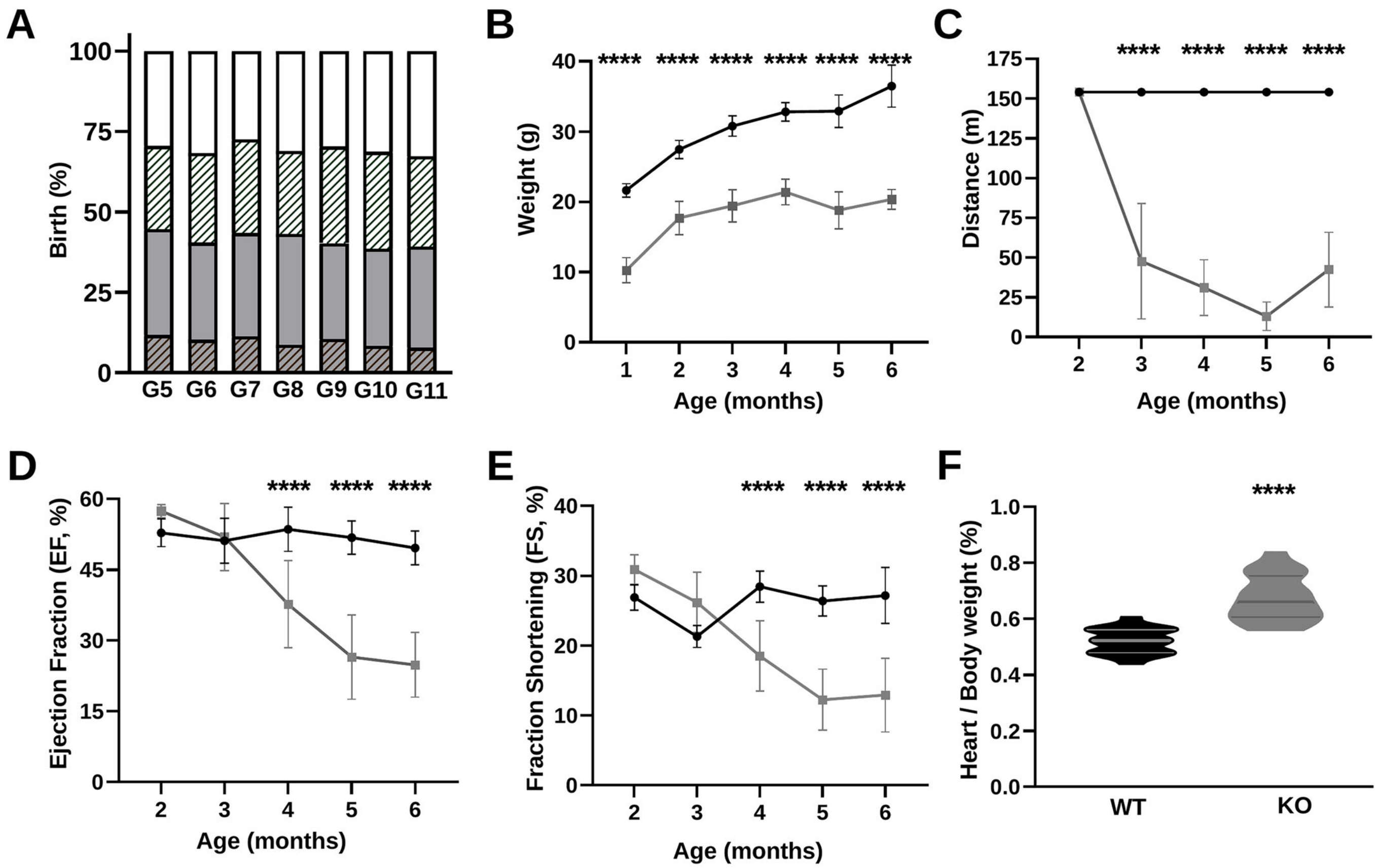

Figure EV2. Characterization of the BTHS mouse model at the whole animal level.

(**A**) Survival of TAZ-KO mice. TAZ-KO mice were present at below the expected Mendelian ratio from birth. Percentage of births from generation 8 to generation 11. WT females are in white bars; HET females are in white striped bars; WT males are in gray bars; KO males are in striped gray bars. (**B**) Growth curve from 1 month to 6 months. Statistical significance between the TAZ-KO and WT groups is shown. *n* is between 18 and 55 according to age. (**C**) Decreased endurance in male TAZ KO mice from 3 months of age. Measurement of treadmill running distance of male WT ($n = 10$ to 52) and KO ($n = 10$ to 32) mice from 2 to 6 months of age. (**D**, **E**) Cardiomyopathy in male TAZ KO mice at 4 months of age. Cardiac parameters measured by echocardiography in male WT ($n = 7$ to 19) and KO ($n = 7$ to 20) mice from 2 to 6 months of age, with measurements of ejection fraction (EF) (**D**) and fraction shortening (FS) (**E**). (**F**) Cardiac hypertrophy in male TAZ KO mice. Ratio of heart weight to animal weight of male TAZ KO mice ($n = 18$) compared to male WT mice ($n = 18$). For (**B–F**), data were presented as mean ± SEM and statistical analyses are performed for TAZ KO mice (gray lines) versus WT mice (black lines) with ***$p < 0.001$ and ****$p < 0.0001$.

© The Author(s)

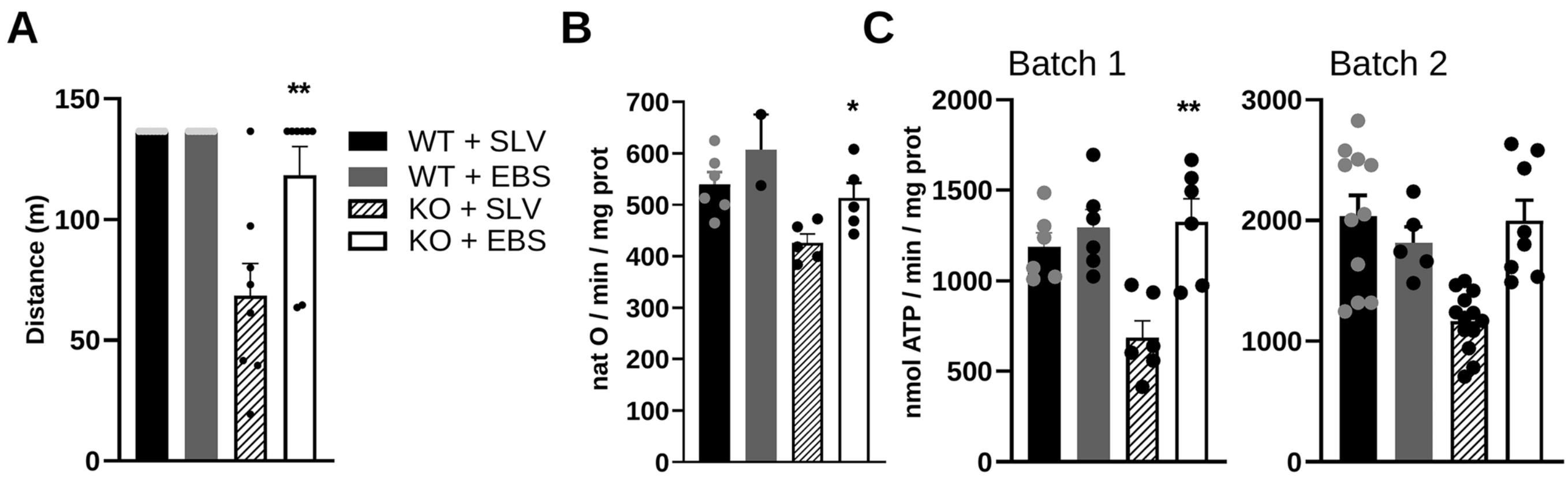

Figure EV3. EBS improves mitochondrial function and exercise capacity in a mouse model of BTHS at 50 mg/kg once daily from weaning until three months of age.

(**A**) Effect of EBS on endurance capacity. Endurance performance was evaluated using a treadmill test. Three-month-old WT treated with solvent (WT SLV), WT treated with EBS (WT EBS), Taz-KO treated with solvent (KO SLV), and Taz-KO treated with EBS (KO EBS) were subjected to forced uphill running until exhaustion. Muscle performance was measured as total running distance. (**B**) Effect of EBS on mitochondrial oxygen consumption. Respiration in intact heart mitochondria was performed in presence of mitochondrial complex II substrate (succinate 10 mM) and respiration was assessed under state 3 (phosphorylating condition, ADP + Pi). (**C**) Effect of EBS on ATP synthesis. ATP production in intact heart mitochondria was measured in presence of mitochondrial CI substrates (PGM: pyruvate 10 mM, glutamate 5 mM, malate 5 mM) and 2.5 mM ADP. Data were expressed as mean ± SEM with $n = 6$ (2 batches of at least $n = 6$ for ATP synthesis analyses). Statistical significance of differences with DMSO-treated mutant cells was estimated using one-way ANOVA followed by Dunnett's multiple-comparisons test. *$p < 0.005$, **$p < 0.01$. WT SLV is shown in black bar, WT EBS in gray, KO SLV in hatched, and KO EBS in white.

© The Author(s)

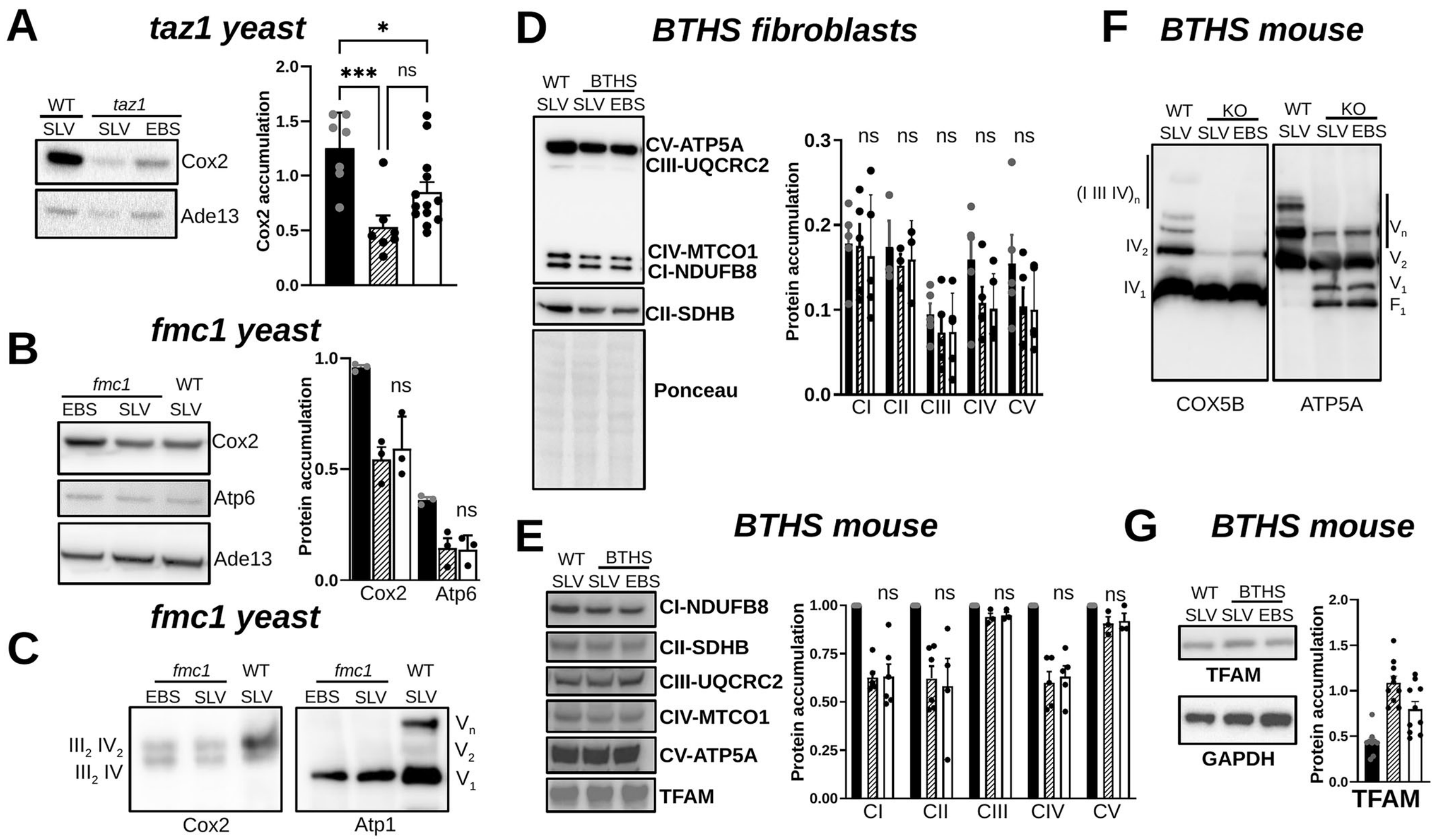

Figure EV4. EBS does not enhance OXPHOS protein accumulation or assembly.

(**A**, **B**) Steady-state levels of proteins in yeast. Total protein extracts from *taz1* (**A**) and *fmc1* (**B**) yeast were separated by SDS-PAGE, transferred onto nitrocellulose membranes, and probed with the indicated antibodies. Protein levels were normalized to Ade13. Mutant cells treated with solvent (SLV) or EBS were compared. Quantification is shown in the right panels. Values represent the mean ± SEM from at least four independent experiments. One dot represents one independent experiment. WT samples are represented by black bars, solvent-treated mutant samples by hatched bars, and EBS-treated mutant samples by white bars. (**C**) Assembly of OXPHOS complexes in yeast. Mitochondrial proteins from *fmc1* yeast cells treated with solvent (SLV) or 1 μM EBS, and from wild-type cells, were separated using Blue Native-PAGE on a 3–12% polyacrylamide gradient and probed with the indicated antibodies. The immunological signals corresponding to ATP synthase oligomers (Vn), dimers (V2), and monomers (V1) and immunological signals corresponding to supercomplexes (III_2IV_2, III_2IV_1) are indicated. (**D**, **E**) Steady-state levels of proteins in human cells and mouse heart. Total protein extracts from BTHS patient fibroblasts (**D**) and mitochondrial protein extracts from BTHS mouse hearts (**E**) were analyzed by SDS-PAGE and immunoblotting. Protein levels were normalized to Red Ponceau (**D**) and TFAM (**E**). Quantification is shown in the right panels. Values represent the mean ± SEM from at least four independent experiments. Statistical analyses were restricted to comparisons between BTHS SLV and BTHS EBS-treated groups. (**F**) Assembly of OXPHOS complexes in the mouse heart. Heart tissue from BTHS mice treated with solvent or EBS and wild-type mice was analyzed by Blue Native-PAGE as described above. The immunological signals corresponding to OXPHOS supercomplexes (I III IV)$_n$ and dimers and monomers of CIV (IV_2 and IV_1) and the immunological signals corresponding to ATP synthase oligomers (Vn), dimers (V2), monomers (V1) and the free particle F_1 are indicated. The same WT SLV and KO SLV images are shown in Appendix Fig. S3G. (**G**) Steady-state protein levels in mouse heart. Mitochondrial protein extracts from BTHS mouse hearts were analyzed by SDS-PAGE and immunoblotting. Protein levels were normalized to GAPDH. Quantification is shown in the right panel. Statistical significance of differences with DMSO-treated mutant cells was estimated using one-way ANOVA followed by Tukey's multiple-comparisons test. *$p < 0.05$, ***$p < 0.001$.

 © The Author(s)

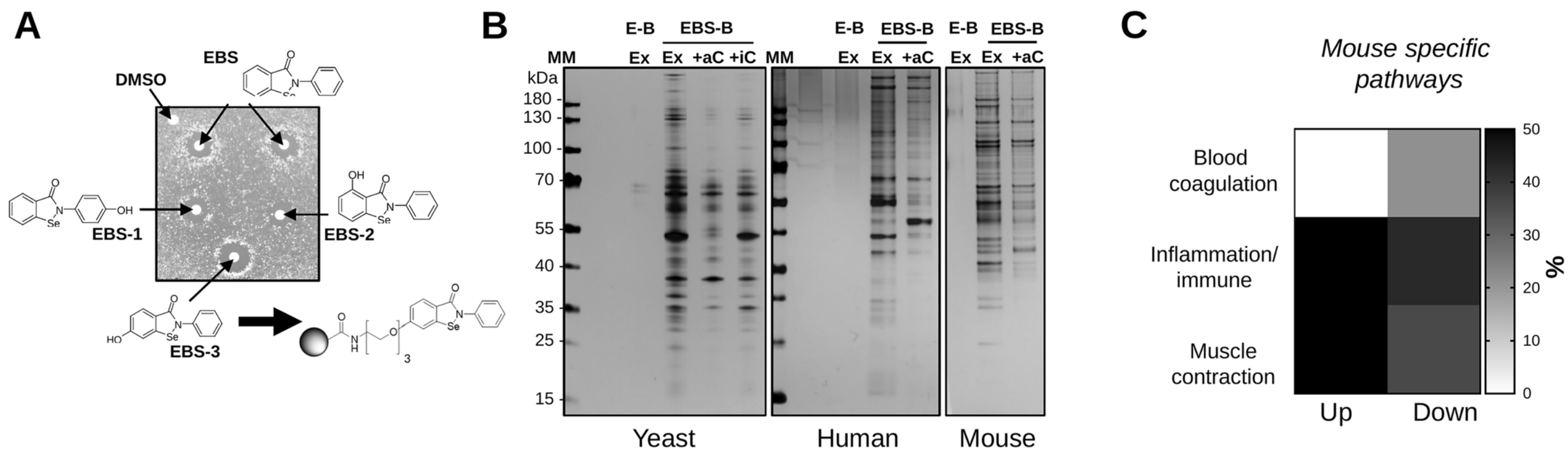

Figure EV5. EBS-interacting proteins across disease models and mouse-specific EBS-regulated proteins.

(**A**) A modified EBS derivatives retain biological activity. A hydroxy group was introduced at positions 1, 2, or 3 of the EBS molecule, as shown in the molecular structures. These derivatives were tested for their ability to retain their capacity to improve respiratory growth of the BTHS yeast model using the same strain and method as depicted in Fig. 1. The molecular structure of the active EBS derivative with a linker at position 3, covalently bound to magnetic beads, is shown in the bottom right. (**B**) Identification of EBS-binding proteins by affinity chromatography. Protein extracts (Ex) from BTHS yeast (left panel), fibroblasts (middle panel) and murine heart tissue (right panel) were incubated with EBS-functionalized magnetic beads (EBS-B). Empty beads (E-B) served as a negative control. Competitive elution was performed using free EBS (aC) or an inactive oxygenized form of EBS (iC, corresponding to EBS form with an oxygen atom shown in Appendix Fig. S1). A portion of the eluted proteins was resolved by SDS-PAGE and silver-stained, while the remainder was subjected to mass spectrometry analysis to identify specific interactors. (**C**) Mouse-specific EBS-regulated proteins. Proteins significantly deregulated following EBS treatment in the BTHS mouse heart were compared with EBS-regulated proteins identified in the yeast and human models. The panel shows proteins deregulated specifically in the mouse model and not shared with the yeast and human datasets.
