# Supplementary material for: Ebselen restores mitochondrial function through metabolic rewiring in Barth syndrome and related mitochondrial diseases
Source: EMBO Mol Med. 2026 Oct 5;18(11):4227–59. doi: 10.1038/s44321-026-00518-1 (PMC13748542; doi:10.1038/s44321-026-00518-1)
Supplement: Supplementary file 2 [file 44321_2026_518_MOESM7_ESM.pdf]

Ebselen restores mitochondrial function through metabolic rewiring in Barth syndrome and related mitochondrial diseases

Table of Contents

Appendix Figures …………………………………………………………………3

Appendix Table ……………………………………………………………….…..8

Appendix Supplementary Information ………………………………………………...12

Compound Synthesis………………………………………………………...12

Synthesis of ebselen oxide…………………………………………………...13

Bead synthesis………………………………………………………………..14

Synthesis of BODIPY-ebselen …………………………………………………19

Appendix Figures

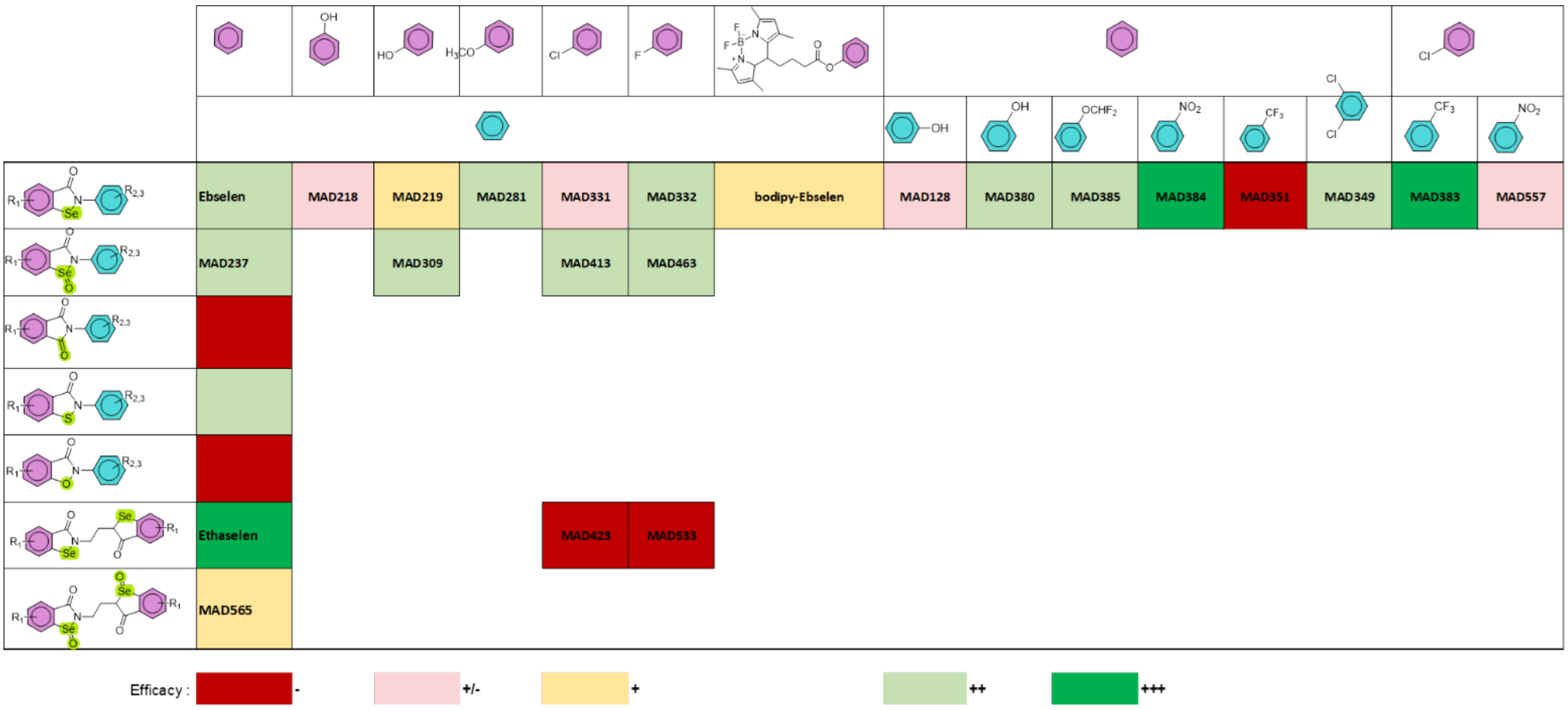

Appendix Figure S1A. Activity of EBS derivatives in BTHS yeast models. Drug assays as depicted in Fig. 1D have been performed on the BTHS yeast model. A classification with + and - symbols has been established according to the halos of efficacy in respect of EBS. See Supplementary Methods for synthesis.

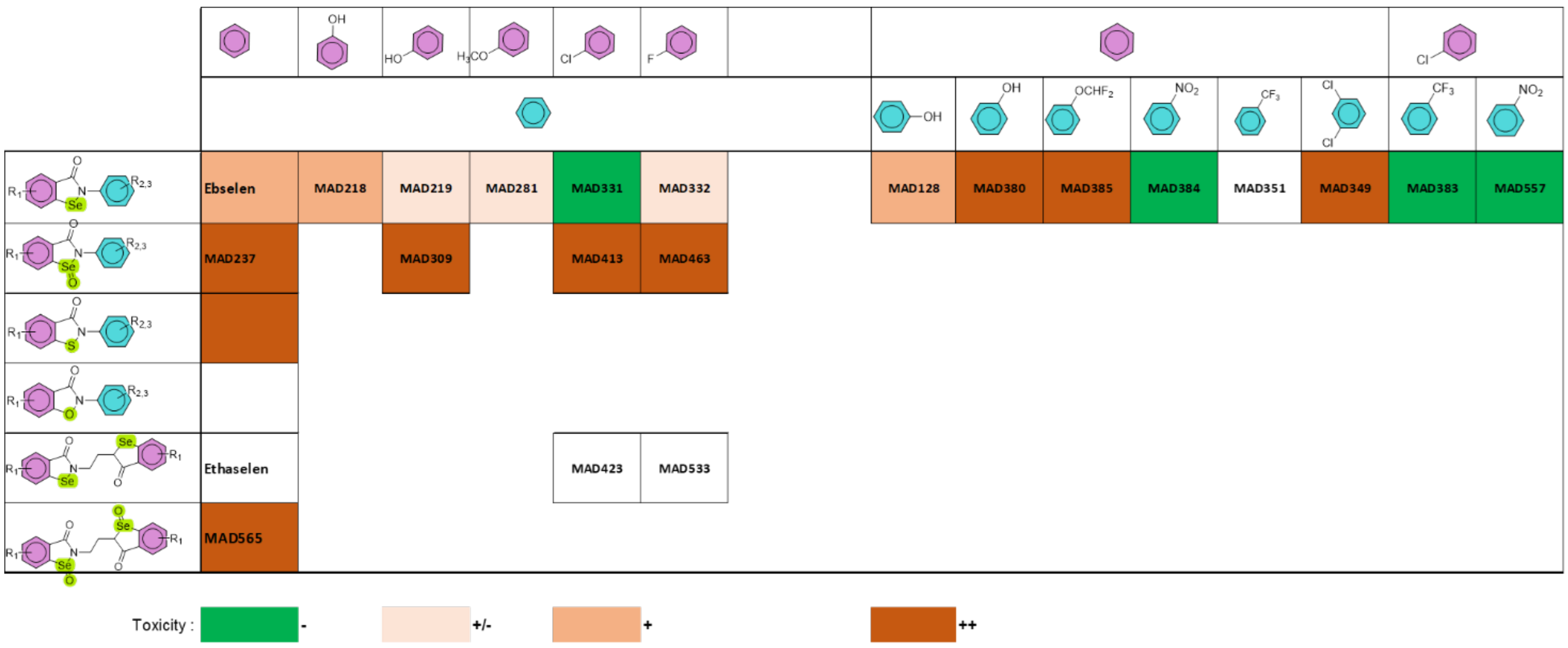

Appendix Figure S1B. Toxicity of EBS derivatives in BTHS yeast models. Drug assays as depicted in Fig. 1D have been performed on the BTHS yeast model. A classification with + and - symbols has been established according to the halos of toxicity in respect of EBS. See Supplementary Methods for synthesis.

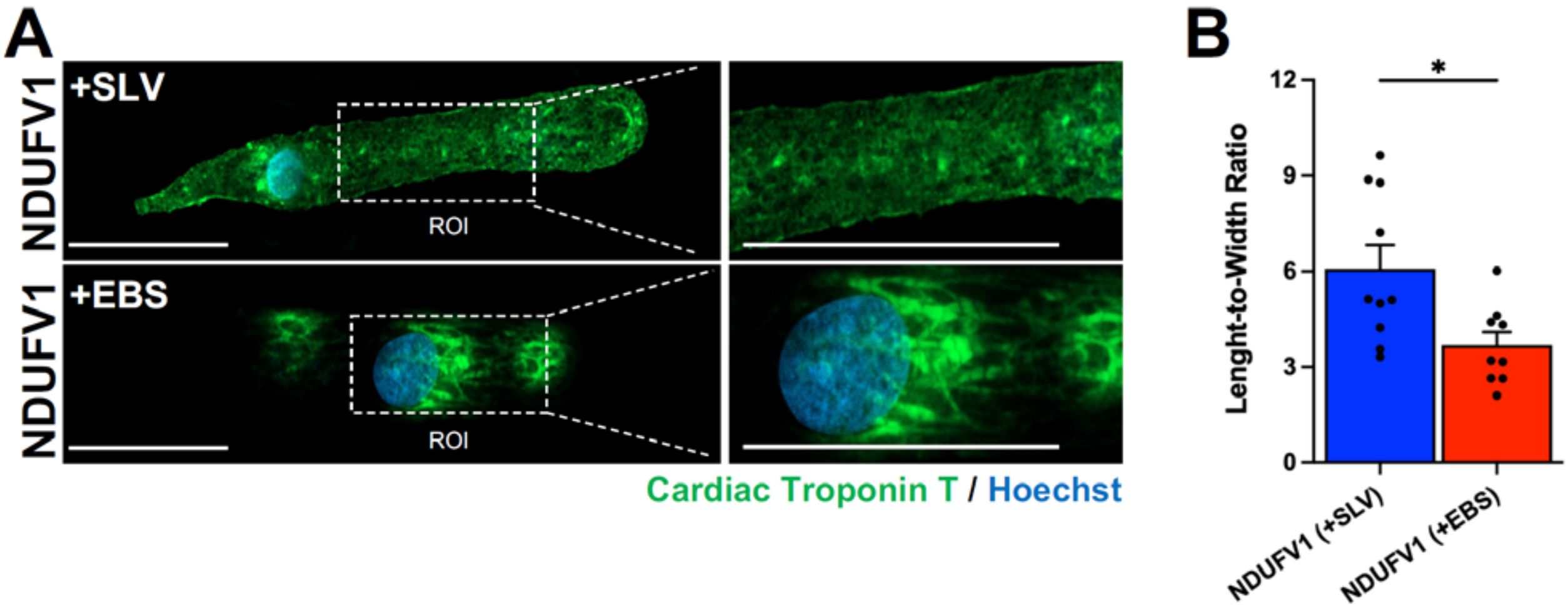

Appendix Figure S2. Morphological impact of EBS in cardiomyocytes were evaluated using an additional iPSC clone derived from Complex I-deficient patient. A. Immunostaining for cardiac Troponin-T (green) was performed on cardiomyocytes cultured on Matrigel-coated micropatterned substrates (20 µm-wide lines) for 14 days post-thawing. Nuclei were counterstained with Hoechst (blue). Scale bar: 50 µm. **B.** Cardiomyocyte length-to-width ratio, was measured using ImageJ software. Values are given as means ± SEM. *, $p < 0.05$; **, $p < 0.01$. NDUFV1 clone 18 + SLV was analyzed from a single differentiation batch (N=1, n=10 cells). NDUFV1 clone 18 + 20 nM EBS was analyzed from a single differentiation batch (N=1, n=9 cells). SLV corresponds to the DMSO vehicle control.

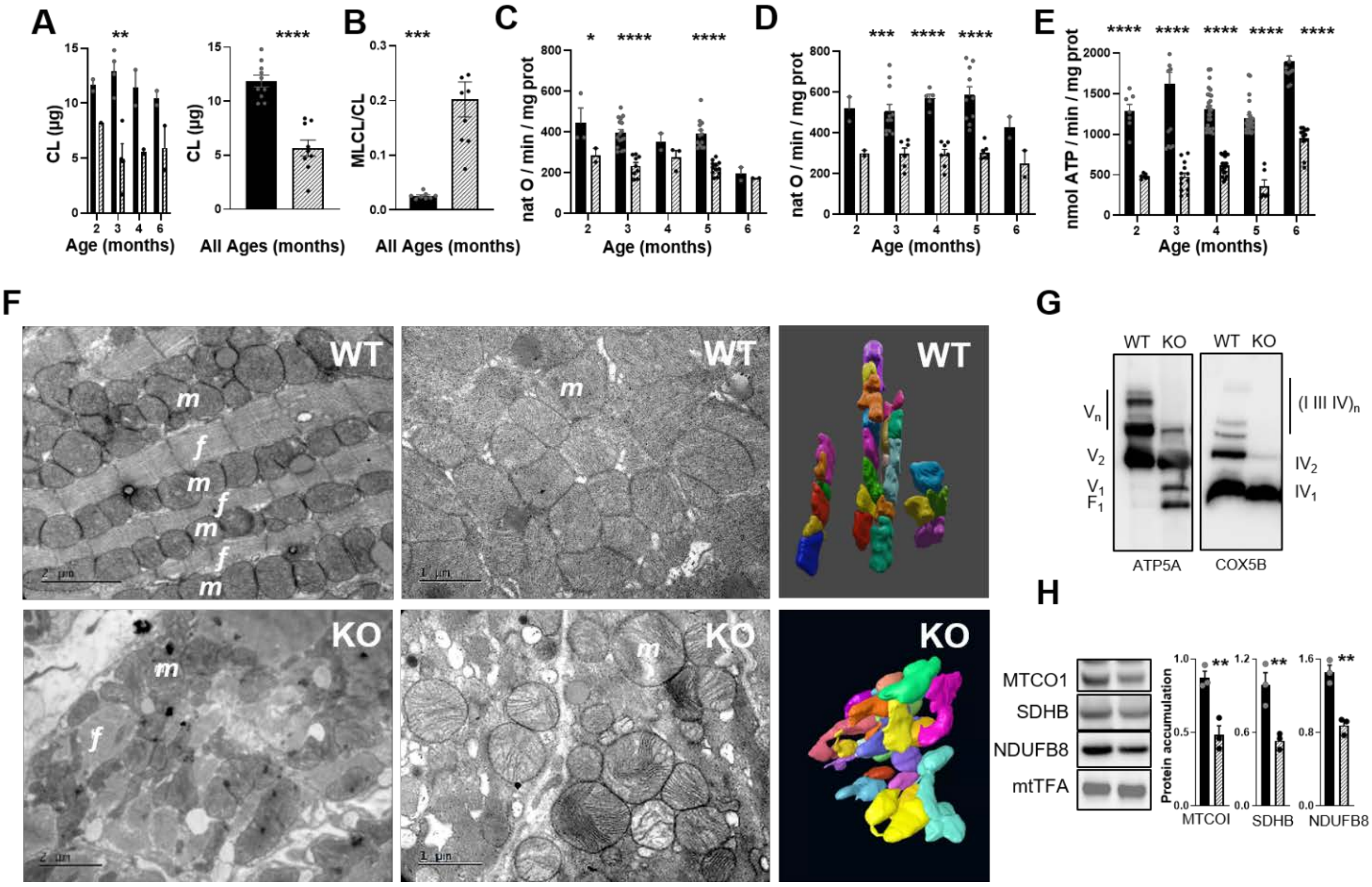

Appendix Figure S3. Characterization of the BTHS mouse model at the heart level. A-B. Decreased levels of mature CL and increased levels of intermediate MLCL in male TAZ KO mice. CL (**A**), and MLCL (**B**) in 100 µg of cardiac mitochondria from 2 to 6 month old WT and TAZ KO mice. **C-D**. Reduced oxygen consumption rate in male TAZ KO mice. Oxygen consumption rate at state 3 (phosphorylating conditions, ADP + Pi) of cardiac mitochondria from WT and TAZ KO mice in the presence of mitochondrial complex I (PGM : 5 mM pyruvate, 5 mM glutamate, 5 mM malate) (**C**) and complex II (10 mM succinate) (**D**) substrates from 2 to 6 months of age. **E.** Reduced ability to synthesize ATP in male TAZ KO mice. Rate of ATP synthesis in cardiac mitochondria of WT and TAZ KO mice from 2 to 6 months of age in the presence of mitochondrial complex I substrates (PGM : 5 mM pyruvate, 5 mM glutamate, 5 mM malate) and 2.5 mM ADP after subtraction of non-mitochondrial ATP by addition of oligomycin. For A, B, C, D, and E, data are presented as the mean of each dot (n=number of dots) ± SEM and statistical analyses are performed for KO mice versus WT mice with *$p<0.1$, **$p<0.01$, ***$p<0.001$ and ****$p<0.0001$. **F.** Disorganisation of muscle fibers and cardiac mitochondria in male TAZ KO mice. Electron microscopy images of heart tissue from WT and TAZ KO mice at 3 months of age in 2D (left panels in black and white) and 3D (right panel in which mitochondria are shown in colour). m is mitochondria, f is fibers. The WT panel is reproduced from Figure 4E. **G.** OXPHOS protein assembly defect in male TAZ KO mice. Mitochondrial proteins isolated from the heart of wild type or BTHS mice of three-month-old were separated in BN gels containing a 3-12% polyacrylamide gradient. The proteins were transferred to a PVDF membrane and probed with the indicated antibodies. The immunological signals corresponding to oligomers (V_n), dimers (V_2), monomers (V_1) and free F_1 particles of ATP synthase and immunological signals corresponding to supercomplexes (I III IV)$_n$, and dimers and monomers of CIV (IV_2 and IV_1) are indicated. The panel from WT SLV and KO SLV is reproduced from Figure EV4F. **H.** OXPHOS protein accumulation defect in male TAZ KO mice. Total mitochondrial protein extracts from wild type and BTHS mouse heart were

resolved by SDS-PAGE, transferred onto a nitrocellulose membrane and probed with the indicated antibodies. The levels of accumulation were normalized to mtTFA. Quantification are shown in the right panels. Values represent the mean ± SEM from 6 mice. WT are in black bar, and KO are in hatched bar.

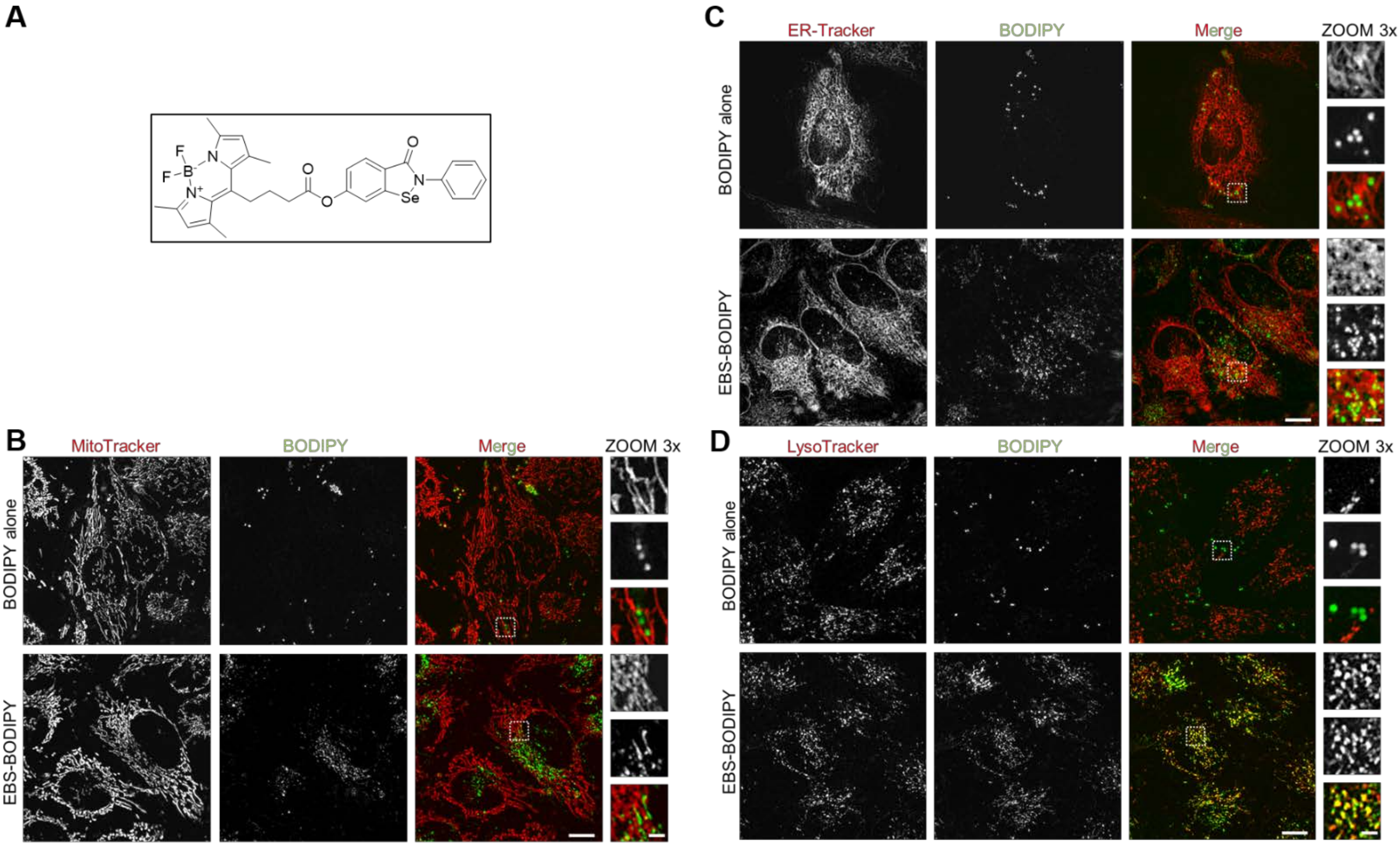

Appendix Figure S4. Localization of BODIPY and EBS coupled to BODIPY. A. *Preparation of EBS-coupled to BODIPY.* See Supplementary Methods for synthesis. **B, C, D.** *Localization of BODIPY and BODIPY coupled to EBS by fluorescence.* BTHS fibroblasts were cutlured in the same conditions as depicted in Fig. 3 with 300 nM of BODIPY or EBS coupled to BODIPY during 24 h. After fixation, cells were stained with a mitotracker (**B**) ER-tracker (**C**) and lysotracker (**D**). Bars are 10 µm.

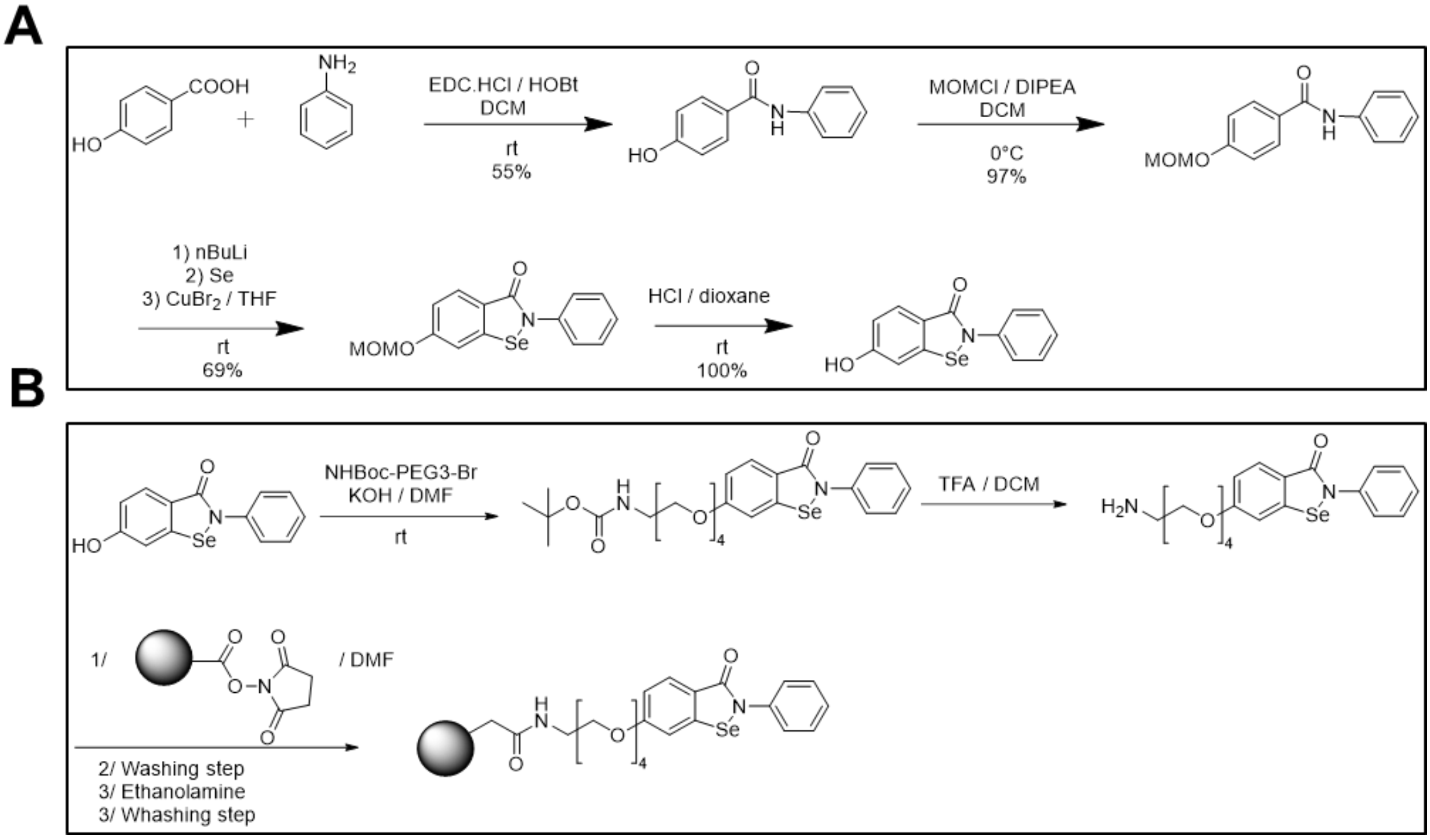

Appendix Figure S5. Preparation of EBS-coupled magnetic beads. A. Synthesis of the modified EBS. **B.** Coupling of modified EBS to beads. See Supplementary Methods for synthesis.

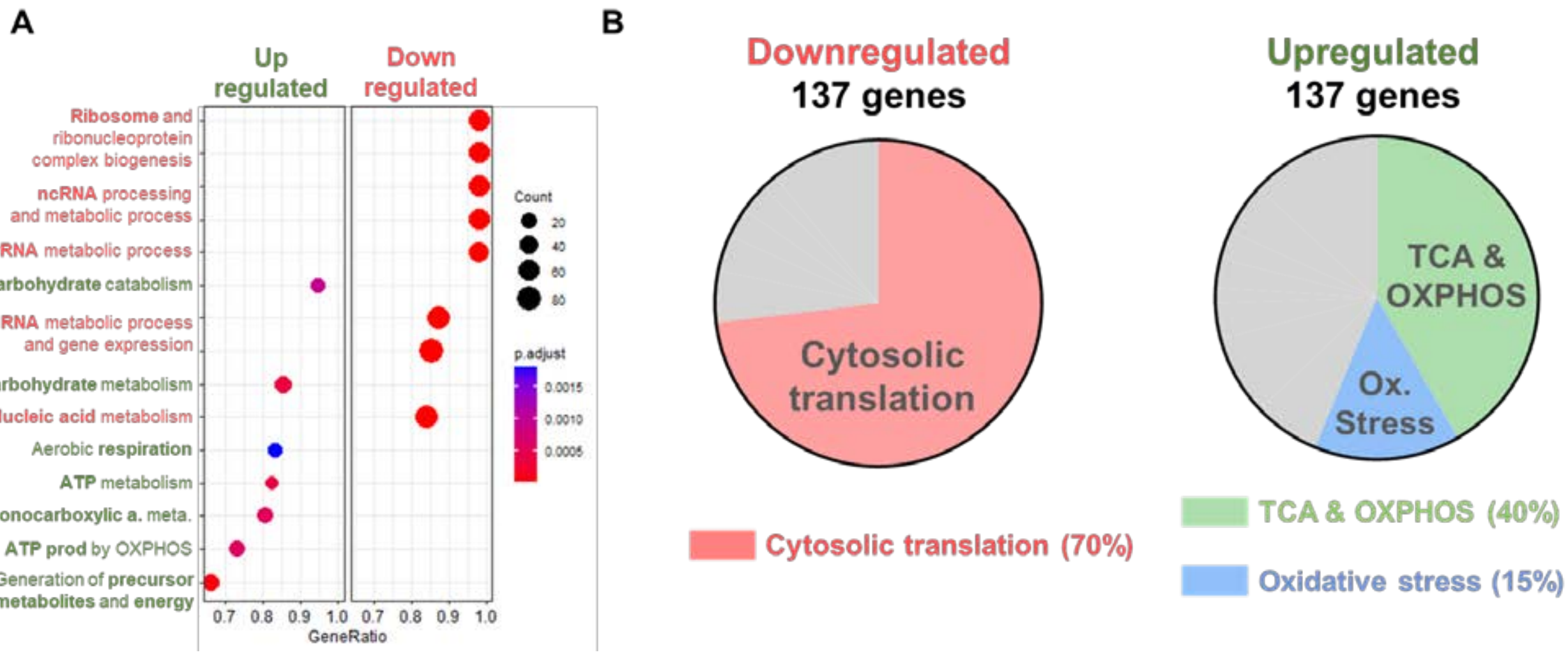

Appendix Figure S6. Transcriptomic analysis in BTHS yeast model. A. KEGG and GO analysis of differentially expressed genes between EBS vs solvent-treated BTHS yeast model. **B.** Graphical representation on the main downregulated (left panel) and upregulated (right panel) pathways after treatment with EBS in the BTHS yeast model.

Appendix Table

Figure	Panel	Readout / parameter	Statistical analysis	Post hoc / correction	Comparison	Exact P value
Fig. 1	B	Respiratory growth	One-way ANOVA	Dunnett's multiple-comparisons test	DMSO vs 1 nM EBS	1.029×10^{-12}
Fig. 1	B	Respiratory growth	One-way ANOVA	Dunnett's multiple-comparisons test	DMSO vs 10 nM EBS	3.600×10^{-14}
Fig. 1	B	Respiratory growth	One-way ANOVA	Dunnett's multiple-comparisons test	DMSO vs 300 nM EBS	9.110×10^{-13}
Fig. 1	B	Respiratory growth	One-way ANOVA	Dunnett's multiple-comparisons test	DMSO vs 1 µM EBS	6.000×10^{-15}
Fig. 1	B	Respiratory growth	One-way ANOVA	Dunnett's multiple-comparisons test	DMSO vs 3 µM EBS	6.000×10^{-15}
Fig. 1	B	Respiratory growth	One-way ANOVA	Dunnett's multiple-comparisons test	DMSO vs 9 µM EBS	6.300×10^{-14}
Fig. 1	B	Respiratory growth	One-way ANOVA	Dunnett's multiple-comparisons test	DMSO vs 27 µM EBS	4.000×10^{-15}
Fig. 1	C	Oxygen consumption	One-way ANOVA	Dunnett's multiple-comparisons test	*taz1* vs WT	3.682×10^{-5}
Fig. 1	C	Oxygen consumption	One-way ANOVA	Dunnett's multiple-comparisons test	*taz1* + SLV vs *taz1* + EBS	1.196×10^{-4}
Fig. 1	D	Cell proliferation	One-way ANOVA	Dunnett's multiple-comparisons test	DMSO vs 1.35 nM EBS	0.008452
Fig. 1	D	Cell proliferation	One-way ANOVA	Dunnett's multiple-comparisons test	DMSO vs 12.15 nM EBS	0.006266
Fig. 1	D	Cell proliferation	One-way ANOVA	Dunnett's multiple-comparisons test	DMSO vs 109.35 nM EBS	1.410×10^{-5}
Fig. 1	D	Cell proliferation	One-way ANOVA	Dunnett's multiple-comparisons test	DMSO vs 984.15 nM EBS	1.660×10^{-7}
Fig. 1	D	Cell proliferation	One-way ANOVA	Dunnett's multiple-comparisons test	DMSO vs 8.7 µM EBS	4.550×10^{-5}
Fig. 1	E	Cell proliferation	One-way ANOVA	Dunnett's multiple-comparisons test	DMSO vs 1.35 nM EBS	0.003613

Fig. 1	E	Cell proliferation	One-way ANOVA	Dunnett's multiple-comparisons test	DMSO vs 12.15 nM EBS	2.248×10^{-4}
Fig. 1	E	Cell proliferation	One-way ANOVA	Dunnett's multiple-comparisons test	DMSO vs 109.35 nM EBS	1.907×10^{-6}
Fig. 1	E	Cell proliferation	One-way ANOVA	Dunnett's multiple-comparisons test	DMSO vs 984.15 nM EBS	1.106×10^{-6}
Fig. 1	E	Cell proliferation	One-way ANOVA	Dunnett's multiple-comparisons test	DMSO vs 8.7 µM EBS	2.162×10^{-6}
Fig. 1	F	Mitochondrial ATP	Mann–Whitney U test	—	DMSO vs 12 nM EBS	1.800×10^{-5}
Fig. 2	C	Oxygen consumption – NADH	One-way ANOVA	Dunnett's multiple-comparisons test	NADH	0.2201
Fig. 2	C	Oxygen consumption – NADH + ADP	One-way ANOVA	Dunnett's multiple-comparisons test	NADH + ADP	0.008910
Fig. 2	C	Oxygen consumption – NADH + ADP + CCCP	One-way ANOVA	Dunnett's multiple-comparisons test	NADH + ADP + CCCP	1.114×10^{-5}
Fig. 3	A′	Length-to-width ratio	Mann–Whitney U test	—	Control vs NDUFV1 + SLV	0.002637
Fig. 3	A′	Length-to-width ratio	Mann–Whitney U test	—	NDUFV1 + SLV vs NDUFV1 + EBS	0.0007569
Fig. 3	A′	Cardiomyocyte volume (μm^3)	Mann–Whitney U test	—	Control vs NDUFV1 + SLV	0.008389
Fig. 3	A′	Cardiomyocyte volume (μm^3)	Mann–Whitney U test	—	NDUFV1 + SLV vs NDUFV1 + EBS	0.01027
Fig. 3	A′	Cardiomyocyte perimeter (µm)	Mann–Whitney U test	—	Control vs NDUFV1 + SLV	0.5059
Fig. 3	A′	Cardiomyocyte perimeter (µm)	Mann–Whitney U test	—	NDUFV1 + SLV vs NDUFV1 + EBS	0.04035
Fig. 3	B′	Mitochondrial area (μm^2)	Mann–Whitney U test	—	Control vs NDUFV1 + SLV	2.742×10^{-5}
Fig. 3	B′	Mitochondrial area (μm^2)	Mann–Whitney U test	—	NDUFV1 + SLV vs NDUFV1 + EBS	0.04571
Fig. 3	C	Contraction amplitude (µm)	Mann–Whitney U test	—	Control vs NDUFV1 + SLV	5.220×10^{-8}

Fig. 3	C	Contraction amplitude (µm)	Mann–Whitney U test	—	NDUFV1 + SLV vs NDUFV1 + EBS	0.002888
Fig. 3	C	Contraction speed (µm/s)	Mann–Whitney U test	—	Control vs NDUFV1 + SLV	3.030×10^{-8}
Fig. 3	C	Contraction speed (µm/s)	Mann–Whitney U test	—	NDUFV1 + SLV vs NDUFV1 + EBS	2.190×10^{-5}
Fig. 3	C	Relaxation speed (µm/s)	Mann–Whitney U test	—	Control vs NDUFV1 + SLV	1.584×10^{-5}
Fig. 3	C	Relaxation speed (µm/s)	Mann–Whitney U test	—	NDUFV1 + SLV vs NDUFV1 + EBS	4.473×10^{-4}
Fig. 3	D	Ascend time (s)	Mann–Whitney U test	—	Control vs NDUFV1 + SLV	1.540×10^{-4}
Fig. 3	D	Ascend time (s)	Mann–Whitney U test	—	NDUFV1 + SLV vs NDUFV1 + EBS	0.008954
Fig. 3	D	Decay time (s)	Mann–Whitney U test	—	Control vs NDUFV1 + SLV	8.560×10^{-8}
Fig. 3	D	Decay time (s)	Mann–Whitney U test	—	NDUFV1 + SLV vs NDUFV1 + EBS	1.160×10^{-5}
Fig. 4	A	Exercise capacity	One-way ANOVA	Tukey's multiple-comparisons test	WT + SLV vs KO + SLV	3.455×10^{-6}
Fig. 4	A	Exercise capacity	One-way ANOVA	Tukey's multiple-comparisons test	WT + SLV vs KO + EBS	0.001137
Fig. 4	A	Exercise capacity	One-way ANOVA	Tukey's multiple-comparisons test	KO + SLV vs KO + EBS	0.02284
Fig. 4	B	Mitochondrial respiration – State 3	ANOVA	Šídák's multiple-comparisons test	WT + SLV vs KO + SLV	8.550×10^{-4}
Fig. 4	B	Mitochondrial respiration – State 3	ANOVA	Šídák's multiple-comparisons test	WT + SLV vs KO + EBS	8.253×10^{-5}
Fig. 4	B	Mitochondrial respiration – State 3	ANOVA	Šídák's multiple-comparisons test	KO + SLV vs KO + EBS	1.440×10^{-10}
Fig. 4	B	Mitochondrial respiration – State 4	ANOVA	Šídák's multiple-comparisons test	WT + SLV vs KO + SLV	> 0.9999
Fig. 4	B	Mitochondrial respiration – State 4	ANOVA	Šídák's multiple-comparisons test	WT + SLV vs KO + EBS	0.9925
Fig. 4	B	Mitochondrial respiration – State 4	ANOVA	Šídák's multiple-comparisons test	KO + SLV vs KO + EBS	0.9653
Fig. 4	B	Mitochondrial respiration – Uncoupled	ANOVA	Šídák's multiple-comparisons test	WT + SLV vs KO + SLV	0.009652

Fig. 4	B	Mitochondrial respiration – Uncoupled	ANOVA	Šídák's multiple-comparisons test	WT + SLV vs KO + EBS	0.2577
Fig. 4	B	Mitochondrial respiration – Uncoupled	ANOVA	Šídák's multiple-comparisons test	KO + SLV vs KO + EBS	5.339×10^{-6}
Fig. 4	C	ATP synthesis	One-way ANOVA	Dunnett's multiple-comparisons test	KO + SLV vs WT + SLV	4.639×10^{-4}
Fig. 4	C	ATP synthesis	One-way ANOVA	Dunnett's multiple-comparisons test	KO + SLV vs KO + EBS	9.880×10^{-5}
Fig. 4	D	Mitochondrial volume	One-way ANOVA	Dunnett's multiple-comparisons test	KO + SLV vs WT + SLV	3.185×10^{-11}
Fig. 4	D	Mitochondrial volume	One-way ANOVA	Dunnett's multiple-comparisons test	KO + SLV vs KO + EBS	0.003347
Fig. 5	A	ROS production	One-way ANOVA	Tukey's multiple-comparisons test	DMSO vs 12.15 nM EBS	4.135×10^{-5}
Fig. 5	A	ROS production	One-way ANOVA	Tukey's multiple-comparisons test	DMSO vs 109.35 nM EBS	1.199×10^{-5}
Fig. 5	A	ROS production	One-way ANOVA	Tukey's multiple-comparisons test	DMSO vs 984.15 nM EBS	1.032×10^{-4}
Fig. 8	B	Cytosolic translation	One-way ANOVA	Tukey's multiple-comparisons test	*taz1* + DMSO vs *taz1* + EBS	0.002186
Fig. 8	B	Cytosolic translation	One-way ANOVA	Tukey's multiple-comparisons test	*fmc1* + DMSO vs *fmc1* + EBS	6.090×10^{-5}
Fig. 8	C	HSP70 abundance	One-way ANOVA	Tukey's multiple-comparisons test	WT vs KO + SLV	0.0005
Fig. 8	C	HSP70 abundance	One-way ANOVA	Tukey's multiple-comparisons test	WT vs KO + EBS	> 0.9999
Fig. 8	C	HSP70 abundance	One-way ANOVA	Tukey's multiple-comparisons test	KO + SLV vs KO + EBS	0.0001
Fig. 8	C	CHOP abundance	One-way ANOVA	Tukey's multiple-comparisons test	WT vs KO + SLV	0.0005
Fig. 8	C	CHOP abundance	One-way ANOVA	Tukey's multiple-comparisons test	WT vs KO + EBS	0.1126
Fig. 8	C	CHOP abundance	One-way ANOVA	Tukey's multiple-comparisons test	KO + SLV vs KO + EBS	0.0559
Fig. 8	D	p-RPS6 abundance	One-way ANOVA	Dunnett's multiple-comparisons test	KO + SLV vs KO + EBS	0.0444
Fig. 8	G	PDK4 abundance	One-way ANOVA	Dunnett's multiple-comparisons test	KO + SLV vs KO + EBS	0.0207

Appendix Table S1. Statistical analyses and exact P-values for the comparisons presented in the main figures. This table summarizes the statistical analyses performed for the data presented in the main figures, including the figure and panel, the experimental comparison, the statistical test used, and the corresponding exact P-value. Exact P-values are provided for all indicated pairwise comparisons. Statistical significance is reported in the main figure legends using the following notation: $P < 0.05$ (*), $P < 0.01$ (**), $P < 0.001$ (***), and $P < 0.0001$ (****). For comparisons for which the exact P-value is not displayed in the main figure legends to avoid overloading the figures, the exact values can be found in this table. SLV refers to solvent-treated samples and EBS to ebselen-treated samples. Statistical tests include one-way ANOVA followed by the indicated multiple-comparison test (Dunnett, Tukey, or Sidak) or the Mann–Whitney test, as specified for each comparison.

Appendix Supplementary Information

Compound Synthesis

Materials :

Reagents and solvents were purchased from Aldrich, Alfa Aesar, Fluorochem, ABCR, Carlo Erba, Acros and used without further purification. Flash column chromatography was carried out on silica gel columns (Interchim Puriflash silica HP 15 to 50 µm) on Puriflash® instrument (Interchim). Analytical thin-layer chromatography (TLC) was conducted on Sigma Aldrich precoated silica gel and compounds were visualized by irradiation (254 nm) and/or by staining with ninhydrin or vanilin stains.

^{1}H and ^{13}C NMR spectra were recorded on Bruker AC 200 and 400 MHz spectrometers. Chemical shifts are reported in parts per million (ppm, δ) referenced to the residual ^{1}H resonance of the solvent (D_2O δ 4.79; $CDCl_3$ δ 7.26; CD_3OD δ 3.31) and referenced to the residual ^{13}C resonance of the solvent ($CDCl_3$ δ 77.2; CD_3OD δ 49.0). Splitting patterns are designed as follows: s (singlet), d (doublet), t (triplet), m (multiplet), and br (broad). Coupling constant (J) are listed in hertz (Hz).

Low resolution mass spectra (MS) were obtained with a Bruker Daltonics Esquire 3000+ electrospray spectrometer equipped with API ionization source. High-resolution mass spectrometry (HRMS) was carried out on a LTQ Orbitrap hybrid mass spectrometer with an electrospray ionization probe (Thermoscientific, San Jose, CA) by direct infusion from a pump syringe, to confirm the correct molar mass and high purity of the compounds.

HPLC was performed using a Waters ARC UHPLC pump coupled to a Waters 2998 photodiode array detector and Waters Cortex® C_{18}+ column (50×4.6 mm, 2.7 µm). Analyses were run at room temperature by using a gradient of CH_3CN containing 0.1 % TFA (eluent B) in water containing 0.1 % TFA (eluent A) at a flow rate of 1.5 mL min^{-1}.

Synthesis of ebselen oxide

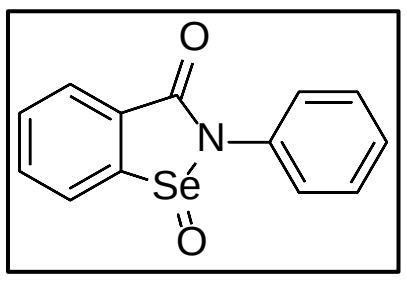

To a cold solution (0°C) of ebselen (50 mg, 0.184 mmol, 1 eq.) in DCM (3 mL) was added H_2O_2 (35% in water, 19 µL, 0.218 mmol, 1.2 eq.). The mixture was stirred overnight at room temperature. The precipitate was filtered, rinsed with DCM and dried to afford the compound as a white solid (23 mg, 43%).

^{1}H NMR (400 MHz, $(CD_3)_2SO$) δ = 8.27-8.23 (m, 1H), 7.96 (dd, J=7.4, 1.0 Hz, 1H), 7.90 (td, J=7.5, 1.4 Hz, 1H), 7.82 (td, J=7.4, 1.1, 1H), 7.52-7.47 (m, 4H), 7.40-7.34 (m, 1H); **^{13}C NMR** (101 MHz, $(CD_3)_2SO$) δ = 166.46, 147.76, 137.09, 134.11, 132.35, 131.15, 129.27, 127.47; 127.17, 126.41; **LCMS** : *m/z* 291.70 [M+H$^+$] (theoretical *m/z* 290.80); **HRMS (ESI)** : *m/z* 291.98715 [M+H$^+$] (theoretical *m/z* 291.98713 for $C_{13}H_{10}O_2NSe$); **HPLC** : t = 3.18 min

Beads synthesis

A/ Synthesis of modified ebselen

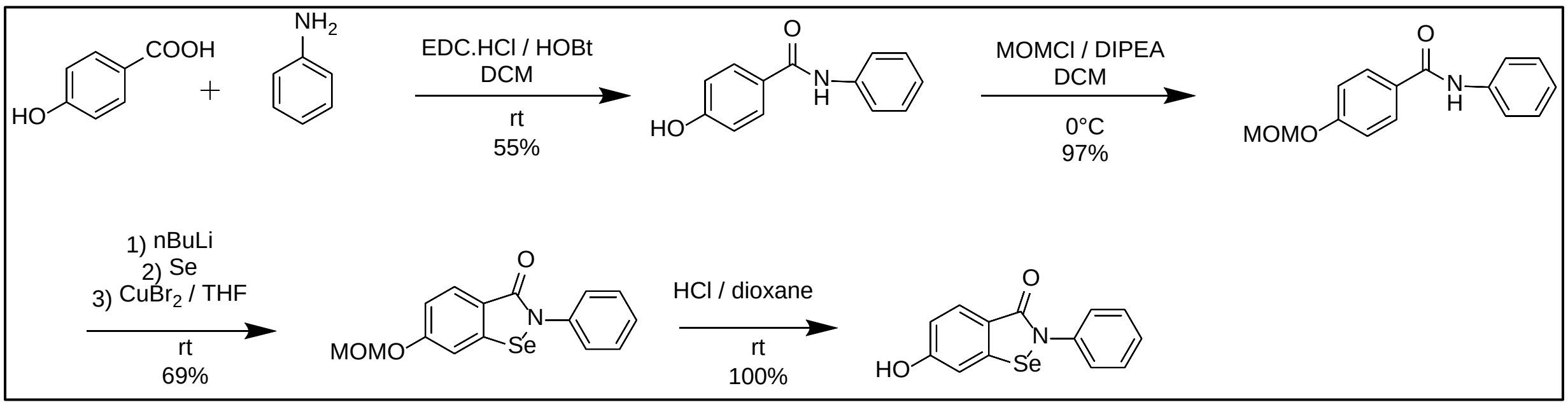

4-hydroxy-N-phenylbenzamide

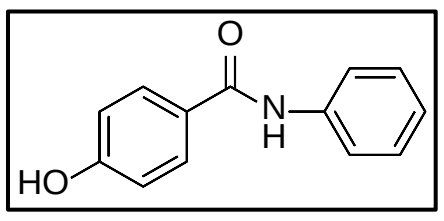

To a suspension of 4-hydroxybenzoic acid (1.38 g, 10 mmol, 1 eq.) in DCM (40 mL) was added EDC.HCl (1.92 g, 10 mmol, 1 eq.), HOBt (1.35 g, 10 mmol, 1 eq.) and aniline (920 µL, 10 mmol, 1 eq.) at 0°C. The mixture was stirred overnight at room temperature, then quenched with water and extracted with DCM. The organic phase was washed with HCl 1M, $NaHCO_3$ (aq) and brine, dried over $MgSO_4$ and the solvent removed under reduced pressure. The crude residue was purified by flash chromatography to afford the compound as a white solid (1.16 g, 55%).

R_f = 0.38 (cHex/EtOAc 6:4); **^{1}H NMR** (400 MHz, CD_3OD) δ = 7.86-7.81 (m, 2H), 7.67-7.63 (m, 2H), 7.37-7.31 (m, 2H), 7.15-7.10 (m, 1H), 6.90-6.85 (m, 2H), 4.57 (s, 1H); **^{13}C NMR** (101 MHz, CD_3OD) δ = 168.70, 162.34, 140.03, 130.68, 129.74, 126.93, 125.38, 122.38, 116.17; **LCMS** : *m/z* 213.80 [M+H$^+$] (theoretical *m/z* 214.08); **HPLC** : t = 4.33 min

MAD260 : 4-(methoxymethoxy)-N-phenylbenzamide

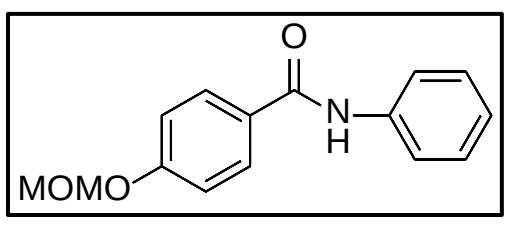

To a solution of 4-hydroxy-N-phenylbenzamide (825 mg, 3.87 mmol, 1 eq.) in dry THF (15 mL) was added DIPEA (2 mL, 11.6 mmol, 3 eq.). After 15 min of stirring at room temperature was added dropwise at 0°C chloromethylmethyl ether (441 µL, 5.81 mmol, 1.5 eq.). The mixture was stirred at 0°C for 1h and then water was added and extracted with EtOAc. The organic phase was dried over $MgSO_4$ and the solvent removed under reduced pressure. The crude residue was purified by flash chromatography to afford the compound as a white solid (955 mg, 97%).

R*f* = 0.50 (cHex/EtOAc 7:3); **^{1}H NMR** (400 MHz, $CDCl_3$) δ = 7.86-7.81 (m, 2H), 7.75 (s, 1H), 7.65-7.60 (m, 2H), 7.37 (dd, J=10.8, 5.2 Hz, 2H), 7.17-7.19 (m, 3H), 5.24 (s, 2H), 3.50 (s, 3H); **^{13}C NMR** (101 MHz, $CDCl_3$) δ = 165.28, 160.25, 138.21, 129.23, 128.96, 128.43, 124.55, 120.28, 116.26, 94.36, 56.37; **LCMS** : *m/z* 257.80 [$M+H^+$] (theoretical *m/z* 257.11); **HPLC** : t = 5.56 min

6-(methoxymethoxy)-2-phenylbenzo[d][1,2]selenazol-3(2H)-one

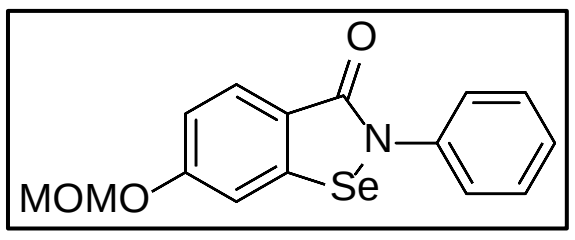

To a solution of 4-(methoxymethoxy)-N-phenylbenzamide (200 mg, 0.78 mmol, 1 eq.) in dry THF (5 mL) at -15°C under an argon atmosphere was added slowly n-BuLi (2.5M in hexane, 932 µL, 2.33 mmol, 3 eq.). The resultant orange solution was stirred at -15°C for 45 min, selenium powder (92 mg, 1.17 mmol, 1.5 eq.) was then added, and the mixture was then stirred for 45 min at -15°C to give a deep red solution. The solution was cooled to -78°C and $CuBr_2$ (521 mg, 2.33 mmol, 3 eq.) was added. The suspension was stirred for 1h at -78°C then removed from the cooling bath and stirred overnight. The resultant brown mixture was poured into 1% AcOH (10 mL) and extracted with EtOAc. The organic fractions were combined, filtered, and

dried over $MgSO_4$ and the solvent reduced under reduced pressure. The crude residue was purified by flash chromatography to afford the compound as a beige solid (126 mg, 48%).

R*f* = 0.25 (cHex/EtOAc 8:2); **^{1}H NMR** (400 MHz, $(CD_3)_2SO$)) δ = 7.81 (d, J=8.6 Hz, 1H), 7.74 (d, J=2.2 Hz, 1H), 7.65-7.60 (m, 2H), 7.47-7.41 (m, 2H), 7.27-7.22 (m, 1H), 7.12 (dd, J=8.6, 2.3 Hz, 1H), 5.31 (s, 2H), 3.43 (s, 3H); **^{13}C NMR** (101 MHz, $(CD_3)_2SO$)) δ = 164.65, 160.06, 140.45, 139.91, 129.15, 125.52, 124.38, 122.38, 115.69, 111.60, 93.98, 55.83; **LCMS** : *m/z* 335.60 [M+H^{+}] (theoretical *m/z* 336.01); **HPLC** : t = 5.67 min

6-hydroxy-2-phenylbenzo[d][1,2]selenazol-3(2H)-one

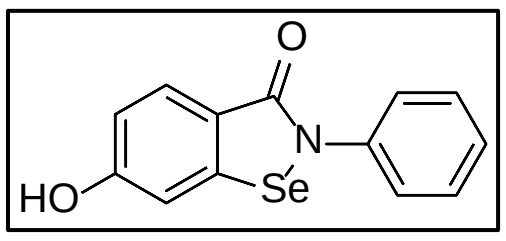

To a solution of 6-(methoxymethoxy)-2-phenylbenzo[d][1,2]selenazol-3(2H)-one (110 mg, 0.33 mmol, 1 eq.) in 1,4 dioxane (5 mL) was added dropwise at 0°C HCl 6N (1.2 mL) and stirred at room temperature for 5h. The reaction was quenched with water and extracted with EtOAc. The organic phase was washed with brine, dried over $MgSO_4$ and the solvent removed under reduced pressure. The crude residue was purified by flash chromatography to afford the compound as a yellow solid (93 mg, 98%).

R*f* = 0.28 (DCM/MeOH 98:2); **^{1}H NMR** (400 MHz, $(CD_3)_2SO$)) δ = 10.37 (s, 1H), 7.70 (d, J=8.5 Hz, 1H), 7.63-7.58 (m, 2H), 7.48-7.40 (m, 3H), 7.26-7.19 (m, 1H), 6.86 (dd, J=8.5, 2.2 Hz, 1H); **^{13}C NMR** (101 MHz, $(CD_3)_2SO$)) δ = 164.95, 161.30, 140.43, 140.12, 129.33, 129.11, 125.29, 124.29, 120.08, 115.09, 111.11; **LCMS** : *m/z* 291.60 [M+H^{+}] (theoretical *m/z* 291.08); **HRMS (ESI)** : *m/z* 291.98703 [M+H^{+}] (theoretical *m/z* 291.98713 for $C_{13}H_{10}O_2NSe$); **HPLC** : t = 4.46 min

B/ Preparation of ebselen coupled beads

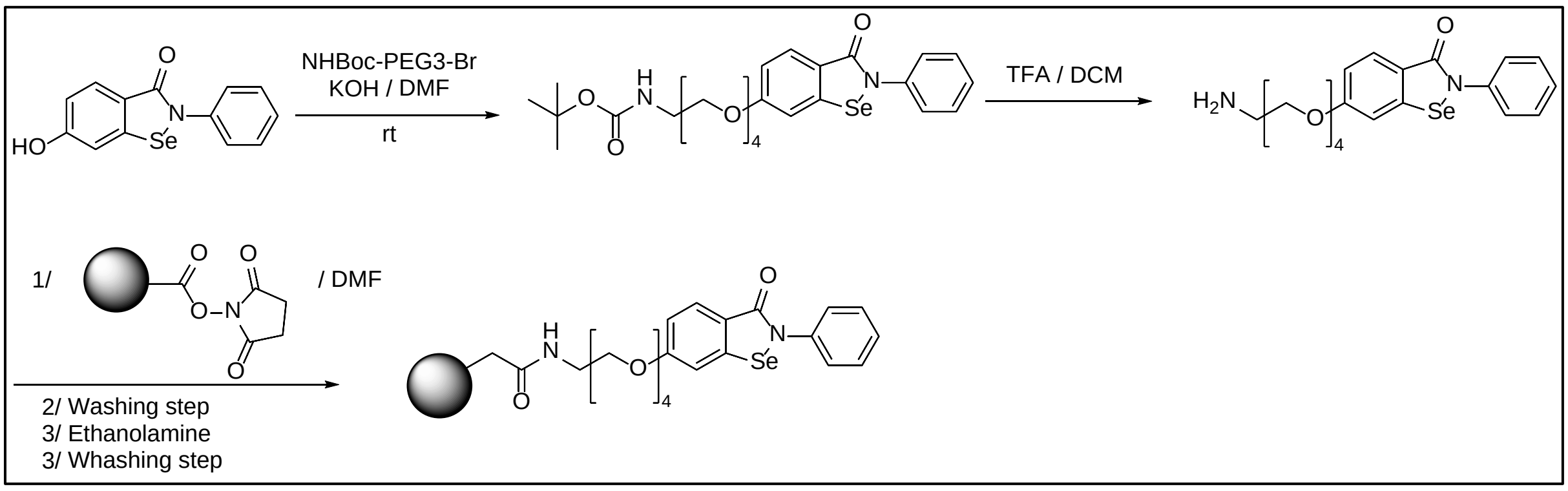

tert-butyl(2-(2(-2-(2-((3-oxo-2-phenyl-2,3-dihydroxybenzo[d][1,2]selenazol-6-yl)oxy)ethoxy)ethoxy)ethoxy)ethyl)carbamate

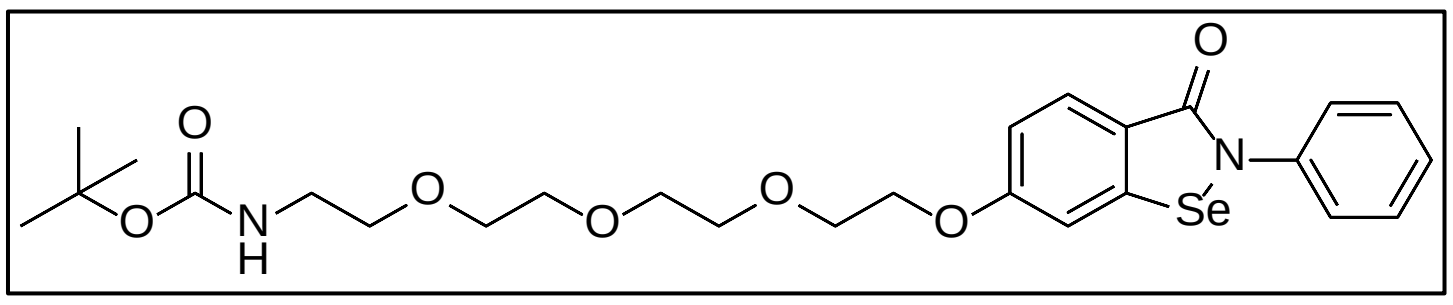

To a solution of 6-hydroxy-2-phenylbenzo[d][1,2]selenazol-3(2H)-one (250 mg, 0.86 mmol, 1 eq.) in dry DMF (5 mL) was added KOH (193 mg, 3.44 mmol, 4 eq.) and the mixture was stirred at room temperature. After 1h was added NHBoc-PEG$_3$-Br (306 mg, 0.86 mmol, 1 eq.) and the reaction was stirred at room temperature overnight. The solvent was removed, water was added and extracted with DCM. The organic phase was dried over $MgSO_4$ and the solvent removed under reduced pressure. The crude residue was purified by flash chromatography to afford the compound as a yellow oil (48 mg, 10%).

R*$_f$* = 0.40 (DCM/MeOH 98:2); **^{1}H NMR** (400 MHz, $CDCl_3$) δ = 7.99 (d, J=8.7 Hz, 1H), 7.64-7.60 (m, 2H), 7.45-7.39 (m, 2H), 7.29-7.23 (m, 2H), 7.15 (s, 1H), 7.02 (dd, J=8.7, 2.2 Hz, 1H), 4.27-4.22 (m, 2H), 3.93-3.89 (m, 2H), 3.77-3.73 (m, 2H), 3.71-3.67 (m, 2H), 3.65 (dd, J=3.4, 1.8 Hz, 2H), 3.63-3.61 (m, 2H), 3.55 (dd, J=9.5, 4.4 Hz, 2H), 3.31 (d, J=5.0 Hz, 2H), 1.44 (s, 9H); **^{13}C NMR** (101 MHz, $CDCl_3$) δ = 165.62, 162.76, 139.57, 139.48, 130.58, 129.42, 120.92,

115.23, 108.31, 71.07, 70.78, 70.75, 70.40, 69.68, 68.23, 28.58; **LCMS** : *m/z* 566.80 [M+H$^+$] (theoretical *m/z* 566.15); **HPLC** : t = 6.12

6-(2-(2-(2-(2-aminoethoxy)ethoxy)ethoxy)ethoxy)-2-phenylbenzol[d][1,2]selenazol-3(2H)-one

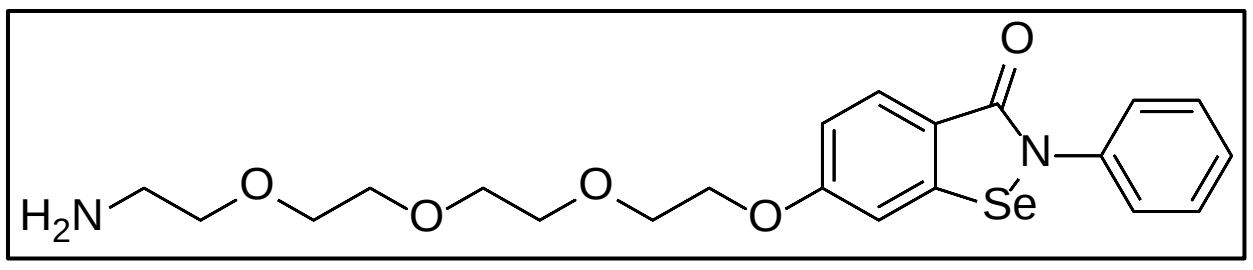

A solution of Ebs-PEG$_3$-NHBoc (30 mg, 0.053 mmol, 1 eq.) in DCM (500 µL) was treated with TFA (40 µL, 0.53 mmol, 10 eq.) for 5h at room temperature. The solvent was removed in vacuo and the crude was co-evaporated with toluene. The crude residue was purified by flash chromatography to afford the compound as a yellow oil (19 mg, 76%).

R*f* = 0.15 (DCM/MeOH 95:5); ^{1}**H NMR** (400 MHz, $CDCl_3$) δ = 7.98 (d, J=8.7 Hz, 1H), 7.61 (dd, J=8.5, 1.0 Hz, 2H), 7.43-7.37 (m, 2H), 7.24 (dd, J=11.7, 4.3 Hz, 1H), 7.20 (d, J=2.1 Hz, 1H), 7.00 (dd, J=8.7, 2.2 Hz, 1H), 4.26-4.21 (m, 2H), 3.91-3.86 (m, 2H), 3.73 (dd, J=5.8, 2.8 Hz, 2H), 3.69-3.65 (m, 4H), 3.64-3.60 (m, 2H), 3.52 (t, J=5.1 Hz, 2H), 2.88 (t, J=5.1 Hz, 2H), 1.74 (s, 3H); ^{13}**C NMR** (101 MHz, $CDCl_3$) δ = 165.66, 162.63, 139.91, 139.70, 130.42, 129.34, 126.33, 125.34, 121.55, 115.13, 108.73, 41.81; **LCMS** : *m/z* 468.80 [M+H$^+$] (theoretical *m/z* 468.10); **HRMS (ESI)** : *m/z* 469.12357 [M+H$^+$] (theoretical *m/z* 469.12362 for $C_{21}H_{29}O_5N_2Se$); **HPLC** : t = 3.83 min

ebselen coupled beads

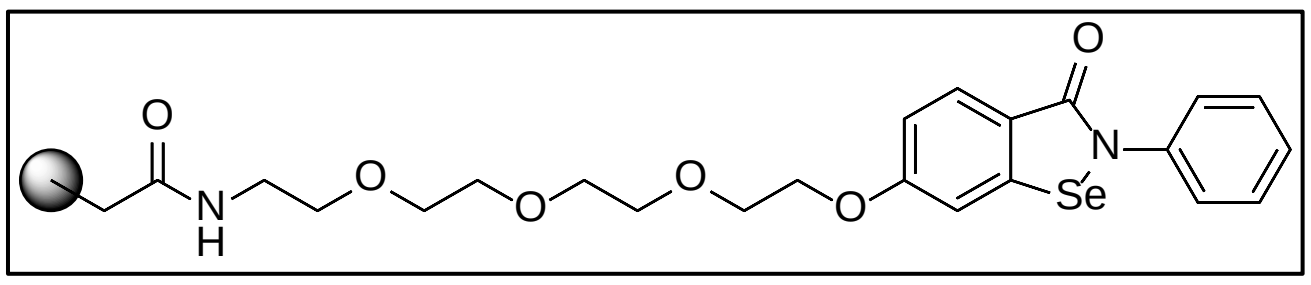

This protocol was established for coupling 0.06 mmol of Ebs-PEG$_3$-NH$_2$ to 1 mL NHS Pierce Activated Magnetic Beads in a 1.5 mL microtube. The reaction can be scaled up or down linearly if required.

Dispense 1 mL of non-coupled NHS Pierce Activated Magnetic Beads into a 1.5 mL microtube and place the tube in a magnetic stand. Remove the supernatant and wash the beads two times with 1 mL of dry DMSO each. Dissolve Ebs-PEG3-NH$_2$ (30 mg, 0.06 mmol, 1 eq.) into 1 mL dry DMSO, add it to the bead following by Et$_3$N (11 µL, 0.08 mmol, 1.2 eq.) and mix by vortexing. Incubate the reaction mixture overnight at r.t. on a tube revolver rotator. Place the microtube into a magnetic stand and discard the supernatant. Wash the magnetic beads two times with 1 mL of dry DMSO. Add ethanolamine (23 µL, 1 mmol, 5eq.) to the bead and mix by vortexing. Incubate the reaction mixture 4h at r.t. on a tube revolver rotator. Place the microtube into a magnetic stand and discard the supernatant. Wash the magnetic beads two times with 1 mL of dry DMSO. Resuspend the beads in 1 mL N’N-dimethylacetamide. Keep at 4°C until use.

<u>Synthesis of BODIPY-ebselen</u>

3-oxo-2-phenyl-2,3-dihydrobenzo[d][1,2]selenazol-6-yl-4-(5,5-difluoro-1,3,7,9-tetramethyl-5H-4l4,5l4-dipyrrolo[1,2-c:2',1'-f][1,3,2]diazaborinin-10-yl)butanoate

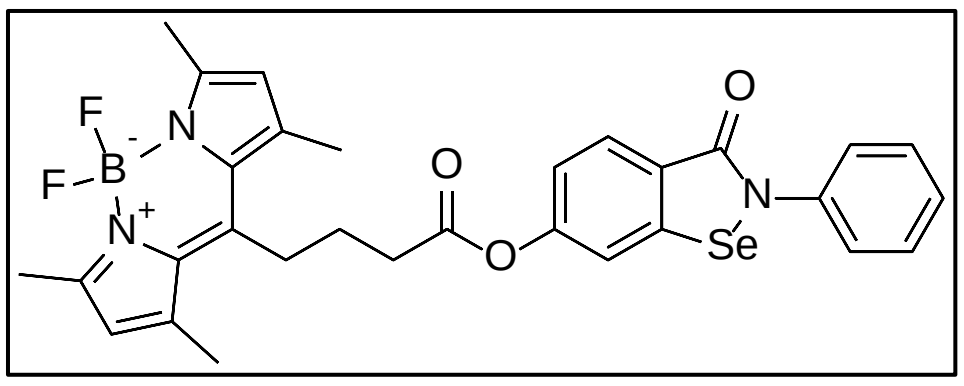

To a solution of carboxyalkyl Bodipy (25 mg, 0.08 mmol, 1 eq.) in THF was added HATU (34 mg, 0.09 mmol, 1.2 eq.) and DIPEA (40 µL, 0.23 mmol, 3 eq.) and was stirred for 15 min at r.t. Then, 6-hydroxy-2-phenylbenzo[d][1,2]selenazol-3(2H)-one (22 mg, 0.08 mmol, 1 eq.) was

added and the mixture was stirred overnight at r.t. The mixture was dissolved in EtOAc and washed with water. The organic phase was dried over $MgSO_4$ and the solvent removed under reduced pressure. The crude residue was purified by flash chromatography to afford the compound as a red solid (38 mg, 35%).

R*f* = 0.26 (DCM/EtOAc 97:3); **^{1}H NMR** (400 MHz, $CDCl_3$) δ = 8.11 (d, *J* = 8.5 Hz, 1H), 7.61 (d, *J* = 7.8 Hz, 2H), 7.49 – 7.40 (m, 3H), 7.30 (t, *J* = 7.4 Hz, 1H), 7.18 (d, *J* = 8.4 Hz, 1H), 6.08 (s, 2H), 3.17 – 3.08 (m, 2H), 2.81 (t, *J* = 7.1 Hz, 2H), 2.53 (s, 6H), 2.46 (s, 6H), 2.15 – 2.06 (m, 2H); **^{13}C NMR** (101 MHz, $CDCl_3$) δ = 170.60, 164.94, 154.58, 153.97, 144.54, 140.48, 139.04, 138.73, 131.60, 130.64, 129.54, 127.06, 125.52, 125.33, 122.10, 120.68, 116.91, 34.38, 27.42, 26.64, 16.67, 14.65; **^{19}F NMR** : δ = (-146.27)-(-146.7); **LCMS** : *m/z* 608.5 [M+H$^+$] (theoretical *m/z* 607.14); **HRMS (ESI)** : *m/z* [M+H$^+$] (theoretical *m/z*); **HPLC** : t = 7.31 min
