# Supplementary material for: Ebselen restores mitochondrial function through metabolic rewiring in Barth syndrome and related mitochondrial diseases
Source: EMBO Mol Med. 2026 Oct 5;18(11):4227–59. doi: 10.1038/s44321-026-00518-1 (PMC13748542; doi:10.1038/s44321-026-00518-1)
Supplement: Supplementary file 3 [file 44321_2026_518_MOESM8_ESM.pdf]

EMBO Molecular Medicine

# Ebselen restores mitochondrial function through metabolic rewiring in Barth syndrome and related mitochondrial diseases

Claire Almyre, Anne-Louise Cayer, Nolwenn Bounaix, Anaïs Hoarau, Marie-Alix Derieppe, Julie Martineau, Pierre Costet, Camille Charles, Mélody Dufossée, Cecile Duplaa, Jim Dompierre, Bénédicte Salin, Marc-Alexandre D'Elia, Corinne Blancard, Aurelie Massoni-Laporte, stephane claverol, Caroline Tokarski, Yaser Hashem, Alexandre FAVEREAUX, Yeranuhi Hovhannisyan, Juliette Jouhet, Cyrille Guimaraes, Jean François Dumas, Olivier Baris, Laetitia Dard, Jérémy Richard, Vanessa Bergeron, Naig Gueguen, Valerie Desquiret-Dumas, Jordan Rivron, Douglas Strathdee, Carole Sellem, Nathalie Bonnefoy, Laurent Monassier, Estelle Ayme-Dietrich, Onnik Agbulut, Stéphane Azoulay, Jean-Paul di Rago, Vincent Procaccio, François Godard, and Deborah Tribouillard-Tanvier

*Corresponding author: Deborah Tribouillard-Tanvier (deborah.tribouillard-tanvier@ibgc.cnrs.fr)*



## Review Timeline:

| | |
|---|---|
| Submission Date: | 1st Mar 26 |
| Editorial Decision: | 26th Mar 26 |
| Revision Received: | 27th Jul 26 |
| Editorial Decision: | 13th Aug 26 |
| Revision Received: | 21st Aug 26 |
| Accepted: | 21st Aug 26 |

*Editor: Zeljko Durdevic*

## Transaction Report:

(Note: With the exception of the correction of typographical or spelling errors that could be a source of ambiguity, letters and reports are not edited. Depending on transfer agreements, referee reports obtained elsewhere may or may not be included in this compilation. Referee reports are anonymous unless the Referee chooses to sign their reports.)

26th Mar 2026

Dear Dr. Tribouillard-Tanvier,

Thank you for the submission of your manuscript to EMBO Molecular Medicine. We have now received feedback from the three reviewers who agreed to evaluate your manuscript. All three referees recognize interest of the study but also raise importnat concerns that should be addressed in a major revision. If you would like to discuss further the points raised by the referees, I am available to do so via email or video. Let me know if you are interested in this option.

Further consideration of a revision that addresses reviewers' concerns in full will entail a second round of review. EMBO Molecular Medicine encourages a single round of revision only and therefore, acceptance or rejection of the manuscript will depend on the completeness of your responses included in the next, final version of the manuscript. For this reason, and to save you from any frustrations in the end, I would strongly advise against returning an incomplete revision.

We would welcome the submission of a revised version within three months for further consideration. Please let us know if you require longer to complete the revision.

I look forward to receiving your revised manuscript.

Yours sincerely,

Zeljko Durdevic

Zeljko Durdevic
Senior Editor
EMBO Molecular Medicine

*****
When preparing your revised manuscript, please refer to our guidelines: https://link.springer.com/journal/44321/submission-guidelines#cms-Revised-submissions. We perform an initial quality control of all revised manuscripts before re-review; failure to include requested items will delay the evaluation of your revision.

We require:

1) A .docx formatted version of the manuscript text (including legends for main figures, EV figures and tables). Please make sure that the changes are highlighted to be clearly visible.

2) Individual production quality figure files as .eps, .tif, .jpg (one file per figure). For guidance, download the 'Figure Guide PDF': https://media.springernature.com/original/springer-cms/rest/v1/content/27825798/data/v1.

3) A .docx formatted letter INCLUDING the reviewers' reports and your detailed point-by-point responses to their comments. As part of the EMBO Press transparent editorial process, the point-by-point response is part of the Review Process File (RPF), which will be published alongside your paper.

4) A complete author checklist, which you can download from our author guidelines. Please insert information in the checklist that is also reflected in the manuscript. The completed author checklist will also be part of the RPF.

5) Please note that all corresponding authors are required to supply an ORCID ID for their name upon submission of a revised manuscript.

6) It is mandatory to include a 'Data Availability' section after the Materials and Methods. Before submitting your revision, primary datasets produced in this study need to be deposited in an appropriate public database, and the accession numbers and database listed under 'Data Availability'. Please remember to provide a reviewer password if the datasets are not yet public.

In case you have no data that requires deposition in a public database, please state so in this section. Note that the Data Availability Section is restricted to new primary data that are part of this study.

7) For data quantification: please specify the name of the statistical test used to generate error bars and P values, the number (n) of independent experiments (specify technical or biological replicates) underlying each data point and the test used to calculate p-values in each figure legend. The figure legends should contain a basic description of n, P and the test applied. Graphs must include a description of the bars and the error bars (s.d., s.e.m.).

8) At EMBO Press we ask authors to provide source data for the main manuscript figures. You will receive a separate email with instructions for providing source data with your revised manuscript, including how to upload and organize the files.

9) Our journal encourages inclusion of *data citations in the reference list* to directly cite datasets that were re-used and obtained from public databases. Data citations in the article text are distinct from normal bibliographical citations and should directly link to the database records from which the data can be accessed. In the main text, data citations are formatted as follows: "Data ref: Smith et al, 2001" or "Data ref: NCBI Sequence Read Archive PRJNA342805, 2017". In the Reference list, data citations must be labeled with "[DATASET]". A data reference must provide the database name, accession number/identifiers and a resolvable link to the landing page from which the data can be accessed at the end of the reference.

10) We replaced Supplementary Information with Expanded View (EV) Figures and Tables that are collapsible/expandable online. A maximum of 5 EV Figures can be typeset. EV Figures should be cited as 'Figure EV1, Figure EV2" etc... in the text and their respective legends should be included in the main text after the legends of regular figures.

- For the figures that you do NOT wish to display as Expanded View figures, they should be bundled together with their legends in a single PDF file called *Appendix*, which should start with a short Table of Content. Appendix figures should be referred to in the main text as: "Appendix Figure S1, Appendix Figure S2" etc.

- Additional Tables/Datasets should be labeled and referred to as Table EV1, Dataset EV1, etc. Legends have to be provided in a separate tab in case of .xls files. Alternatively, the legend can be supplied as a separate text file (README) and zipped together with the Table/Dataset file.

11) The paper explained: EMBO Molecular Medicine articles are accompanied by a summary of the articles to emphasize the major findings in the paper and their medical implications for the non-specialist reader. Please provide a draft summary of your article highlighting

- the medical issue you are addressing,

- the results obtained and

- their clinical impact.

This may be edited to ensure that readers understand the significance and context of the research. Please refer to any of our published articles for an example.

12) Author contributions: You will be asked to provide CRediT (Contributor Role Taxonomy) terms in the submission system. These replace a narrative author contribution section in the manuscript.

13) A Conflict of Interest statement should be provided in the main text.

14) Every published paper includes a 'Synopsis' to further enhance discoverability. Synopses are displayed on the journal webpage and are freely accessible to all readers. They include a short stand first (maximum of 300 characters, including space) as well as 2-5 one-sentences bullet points that summarizes the paper. Please write the bullet points to summarize the key NEW findings. They should be designed to be complementary to the abstract - i.e. not repeat the same text. We encourage inclusion of key acronyms and quantitative information (maximum of 30 words / bullet point). Please use the passive voice. Please attach these in a separate file or send them by email, we will incorporate them accordingly.

Please also suggest a striking image or visual abstract to illustrate your article as a PNG file 550 px wide x 300-600 px high.

15) Include a Reagents and Tools Table as part of the Methods section, which can be downloaded from our author guidelines.

EMBO Molecular Medicine has a "scooping protection" policy, whereby similar findings that are published by others during review or revision are not a criterion for rejection. Should you decide to submit a revised version, I do ask that you get in touch after three months if you have not completed it, to update us on the status.

***** Reviewer's comments *****

Referee #1 (Remarks for Author):

Mitochondrial diseases are a group of fatal hereditary disorders with very limited therapeutic options. Through a powerful yeast-based screening approach, Almyre et al. identified the EBS compound, an FDA-approved drug, as a potential therapeutic agent for Barth syndrome and other mitochondrial disorders. Moreover, EBS is effective at improving mitochondrial respiration in evolutionarily distant models, from yeast to human cells and mice. These findings support the conclusion that EBS represents a promising therapeutic candidate for a broad spectrum of mitochondrial disorders. However, EBS's mechanism of action remains largely speculative. The hypothesis that a mild decrease in cytosolic translation could be beneficial by decreasing proteotoxic stress needs to be tested experimentally.
Additional points requiring clarification:
- EBS-BODIPY localizes mainly to lysosomes. However, non-lysosomal proteins are identified by EBS-based affinity chromatography plus MS. The authors should reconcile this apparent discrepancy.
- What could be the mechanism underlying the EBS-dependent increase in respiration measured in isolated yeast mitochondria in the presence of NADH as a substrate? By adding an excess of NADH, TCA activity is bypassed.
Minor points:
- Figure 1D: please indicate when the pictures were taken.
- Figures 3D-F and 7B-D: some lines and SD bars are not clearly visible.
- Figure 8: please include a loading control for the BN-PAGE blots.

Referee #2 (Comments on Novelty/Model System for Author):

The authors have tested the compound of interest in a wide range of human cellular models and yeast models of mitochondrial disease

Referee #2 (Remarks for Author):

Almyre and colleagues have identified Ebselen, an organoselenium compound in phase II and phase III clinical trials for other disorders, with known antioxidant and anti-inflammatory properties that might be a promising candidate for the treatment of mitochondrial dysfunction, given it has shown to be able to cross the blood-brain barrier.

In this manuscript, the authors identified this compound through it's ability to enhance the growth of a yeast model of Barth syndrome, in which cardiolipin remodelling contributes to the pathophysiological disorder associated with the disease phenotype. Ebselen not only enhanced respiratory growth but also improved respiration and $O_2$ consumption in taz1 yeast cells, prompting a deeper analysis in other yeast and fungal models of mitochondrial dysfunction.

The authors propose a novel mechanism of action, involving downregulation of cytosolic translation to restore proteostasis and activation of the TCA cycle via PDHc, to stimulate ATP production, suggesting a broad effect of the drug in modulating mitochondrial function.

The work is nicely presented and discussed and the paper well written, although as I read through this I am increasingly struggling to look for the key data and conclusions amongst 13 Figures and supplemental data. I have a number of questions and comments for the authors:

1. Why did the authors start the work using a BTHS yeast model, it would be helpful to understand further the path to which this work started and why this model - ahead of say a model with a primary mitochondrial defect - was used?

2. Ebselen was shown not to increase CL levels in the BTHS yeast model, but treatment was associated with longer-chain species of cardiolipin; what is the proposed mechanism of this? Were experiments also undertaken in other cell models of primary mitochondrial CL deficiency (ie TAMM41 deficiency)?

3. A general question, but the selection of cellular models to exlore the therapeutic effects of Ebselen, does appear (to this reviewer at least) to be rather arbitary......."next we looked at CI deficiency and the ATP synthase deficiency model" - is this simply explained by the availability of models to the laboratory, or is their a further scientific rationale to this selection? What is

the cause of the cytotoxicity at concentrations of Ebselen greater than 5uM?

4. I am less convinced, or satisfied, by the n=1 experiment with NDUFV1 iPSC cardiomyocytes - were other lines not available? Did you attempt to rescue any mitochondrial defects in other iPSC cardiomyocyte lines? Page 9 - I do not think that you can say that the drug "improves the structure and mitochondrial defects of cardiomyocytes derived from patients with CI deficiency"

5. Experiments with the Taz-KO mouse model - I think it would be nice to incorporate some of the data currently in the Supplement in the main Figure (Figure 5) - please consider this

6. The studies to investigate the effect on Ebselen on OxPhos protein accumulation, stability and assembly are shown in Figure 8 and rely on SDS-PAGE and immunoblotting data; the authors did undertake proteomic analysis, in the treated v untreated mouse tissues, but the manuscript does not really provide a full analysis of the effects at protein level on a whole range of mitochondrial proteins, offering further mechanistic insight. It does appear to be something of a missed opportunity given the access to mouse tissues from the KO......I'm not overly clear from the data in the main paper or the supplement how many proteins were identified or how the analysis was undertaken to identify interactors.

Overall, there is a significant body of evidence but the large number of Figures and supplemental data suggest that a simpler message and narrative would help to convince the reader of the mechanism and impact of this work.

Referee #3 (Comments on Novelty/Model System for Author):

With this research work, a new therapeutical molecule is proven to rescue different mitochondrial defects in different models (yeast, human cells and mice). The authors addressed not only the rescue of different mito-defects but also try to elucidate the mechanism of action. Although some molecular details are still missing, this has high medical potential, as treatment for mito-diseases.

Referee #3 (Remarks for Author):

Almyre and co-workers present a sound and well-written study demonstrating that EBS, as a novel therapeutic molecule, is capable of rescuing different mitochondrial defects across multiple models (yeast, human cells, and mice). The authors not only address the rescue of diverse mitochondrial dysfunctions but also attempt to elucidate the mechanism of action. Although some molecular details remain unresolved, this work has strong medical potential, is highly relevant to the field, and positions EBS as a promising therapeutic candidate for mitochondrial diseases. However, several aspects should be addressed prior to publication.

In Figure 1, when assessing EBS-mediated rescue across different models of mitochondrial dysfunction, the growth halos do not appear comparable across all systems. The authors could quantify these differences and discuss any potential differential effects of EBS on distinct mitochondrial defects.

In Figure 3D, the Seahorse measurements are not clearly presented. It would be helpful to include color-coded graphs or to highlight the most relevant EBS concentration in the main manuscript, with additional data provided in the supplementary information. It would also be interesting to assess cardiolipin content or lipid distribution following EBS treatment in BTHS cell lines. In addition, and in relation to the reported effects of EBS on translation, what are the TAZ protein levels in patient-derived cells after EBS treatment?

Regarding mutant cardiomyocytes treated with EBS, are there functional parameters (e.g., beating rate, $Ca^{2+}$ signaling, ATP production, or similar readouts) that are impaired in patient cells and can be restored upon treatment?

The results presented in Figure 8 appear somewhat surprising. First, in yeast models, there is an apparent increase in Cox2 upon EBS treatment based on both the Western blot (taz1 and fmc1) and quantifications (taz1), which is not discussed by the authors. In addition, the Western blot panel for BTHS fibroblasts would benefit from the inclusion of a control cell line, as in the other experiments. If EBS does not affect OXPHOS assembly, this could potentially not explain the observed increases in OCR and growth. What alternative hypotheses do the authors consider? One possible explanation is the use of different sample sources across experiments: OCR measurements are performed in whole cells, whereas Western blots are likely derived from isolated mitochondria. An increase in mitochondrial mass per cell could account for the observed rescue of respiration, while OXPHOS subunit levels per mitochondrion (as assessed by Western blot) may remain unchanged. The authors could

investigate whether mitochondrial mass is increased, potentially as a result of mitochondrial biogenesis.

EBS appears to interact with multiple cellular targets and regulate major pathways that may contribute to improved mitochondrial function. However, beyond its reported effects on the PDC complex and the TCA cycle, the connection between EBS and mitochondrial rescue remains unclear. A critical aspect of this study would be to better link EBS-mediated modulation of mTOR to cytosolic translation inhibition and the potential enhancement of mitochondrial respiration and cellular growth, possibly through mitochondrial biogenesis. Have the authors considered the involvement of the mt-UPR/ISR pathway in relation to mTOR signaling?

***** Reviewer's comments *****

**Referee #1 (Remarks for Author):**

Mitochondrial diseases are a group of fatal hereditary disorders with very limited therapeutic options. Through a powerful yeast-based screening approach, Almyre et al. identified the EBS compound, an FDA-approved drug, as a potential therapeutic agent for Barth syndrome and other mitochondrial disorders. Moreover, EBS is effective at improving mitochondrial respiration in evolutionarily distant models, from yeast to human cells and mice. These findings support the conclusion that EBS represents a promising therapeutic candidate for a broad spectrum of mitochondrial disorders. However, EBS's mechanism of action remains largely speculative.

1. The hypothesis that a mild decrease in cytosolic translation could be beneficial by decreasing proteotoxic stress needs to be tested experimentally.

We thank the reviewer for this constructive comment. We agree that the hypothesis that a mild reduction in cytosolic translation induced by EBS could help reduce proteotoxic stress is very relevant in the context of mitochondrial diseases and deserves further attention. This idea is supported by our previous study showing that partial inhibition of cytosolic translation, either with cycloheximide or through mutations in ribosomal proteins, can improve mitochondrial function in a yeast model of Barth syndrome (de Taffin et al., 2018). It is also well established that defects in mitochondrial protein import can lead to cytosolic proteotoxic stress, due to the accumulation of non-imported mitochondrial precursor proteins. Cardiolipin is essential for the proper function of the TIM23 and TIM22 import systems, and its deficiency impairs mitochondrial protein import (Kutik et al., 2008). This in turn activates protein quality control and stress response pathways (Wrobel et al., 2015). Based on this, we hypothesized that reducing cytosolic protein synthesis could help limit this proteostatic burden. To test this, we analysed markers of proteostasis in our BTHS mouse model. We observed increased HSP70 together with CHOP induction in untreated animals, consistent with activation of protein stress pathways. Both markers were reduced after EBS treatment (updated in Fig. 13C). We also show in the manuscript that EBS reduces cytosolic protein synthesis (Fig. 13B), which fits with this interpretation. Overall, these results are consistent with a model in which EBS helps alleviate proteotoxic stress, at least partly by reducing cytosolic protein synthesis. The manuscript has been updated accordingly in Fig. 13, figure legends, results and discussion.

Additional points requiring clarification :

2. EBS-BODIPY localizes mainly to lysosomes. However, non-lysosomal proteins are identified by EBS-based affinity chromatography plus MS. The authors should reconcile this apparent discrepancy.

We thank the reviewer for this important comment. Although EBS-BODIPY mainly accumulates in lysosomes, this does not contradict the identification of non-lysosomal proteins by EBS-based affinity chromatography. Lysosomes are now well known to act as signalling hubs and to form functional contacts with other cellular compartments, in particular in the context of mTOR signalling, where several components are recruited to the lysosomal surface rather than being permanent lysosomal residents. In our affinity chromatography experiments, we identified MEAK7, a protein linked to the mTOR pathway, as an EBS-interacting partner. To further address the reviewer's concern, we performed additional co-localization experiments. These show that EBS co-localizes with mTOR, that mTOR co-localizes with the lysosomal marker LAMP2, and that EBS also partially co-localizes with LAMP2. Taken together, these data suggest that EBS is associated with lysosome-associated mTOR signalling complexes, which is consistent with both its predominant lysosomal localization and

the identification of mTOR-related interactors in the affinity purification. These results have now been included in Fig. 10 and the corresponding parts of the results, figure legends and discussion have been updated.

3. What could be the mechanism underlying the EBS-dependent increase in respiration measured in isolated yeast mitochondria in the presence of NADH as a substrate? By adding an excess of NADH, TCA activity is bypassed.

We thank the reviewer for this important comment. We agree that in these experiments respiration was measured in isolated yeast mitochondria supplied with exogenous NADH, which bypasses the requirement for pyruvate oxidation and Krebs cycle activity. Therefore, the increase in oxygen consumption observed in the presence of EBS cannot be explained by stimulation of the pyruvate dehydrogenase complex or the TCA cycle. Importantly, our data show that EBS does not significantly affect the abundance or assembly of OXPHOS complexes. However, stimulation of mitochondrial respiration does not necessarily require changes in OXPHOS complex assembly. Respiratory flux can increase through improved electron transfer efficiency or changes in the mitochondrial functional state without detectable alterations in the organization of the respiratory chain complexes. In yeast, exogenous NADH feeds electrons into the respiratory chain through alternative NADH dehydrogenases rather than through complex I. EBS may therefore modestly improve the efficiency of electron transfer through these pathways or slightly enhance the functional state of the mitochondrial respiratory chain. Consistent with this interpretation, the increase in respiration observed in isolated yeast mitochondria remains relatively small and far from reaching wild-type levels. In contrast, in isolated mouse heart mitochondria supplied with pyruvate, glutamate and malate, which feed mitochondrial metabolism upstream of the respiratory chain, EBS treatment restores respiration to levels comparable to those observed in wild-type mitochondria. This observation suggests that the major metabolic effect of EBS likely occurs upstream of the respiratory chain under physiological substrate conditions. We have clarified this point in the discussion of the revised manuscript to better distinguish these experimental contexts and to avoid overinterpretation of the modest effect observed with NADH in yeast mitochondria.

**Minor points:**

4. Figure 1D: please indicate when the pictures were taken.

We thank the reviewer for this suggestion. We added the information in the revised manuscript (updated Fig. 3A).

5. Figures 3D-F and 7B-D: some lines and SD bars are not clearly visible.

We thank the reviewer for this suggestion. We agree that highlighting key concentrations and improving the visual presentation makes the data easier to follow. In the revised manuscript, we have added color-coded traces for each EBS concentration in updated Fig. 3 and Fig. 9 to facilitate comparison and highlighted the most relevant active concentrations (12 nM to 3µM for BTHS fibroblasts).

6. Figure 8: please include a loading control for the BN-PAGE blots.

We thank the reviewer for this suggestion. Unfortunately, we are not able to include an additional loading control for these BN-PAGE experiments because the mitochondrial extracts used for these analyses are no longer available. More importantly, BN-PAGE is primarily designed to assess the assembly state of respiratory chain complexes and to detect assembly intermediates rather than to provide quantitative measurements of protein abundance, and

classical loading controls are therefore rarely used in this context. In our experiments, equal amounts of mitochondrial protein were loaded in each lane, allowing comparison of assembly patterns between conditions. In addition, the overall protein signal across lanes appears comparable, supporting similar loading. Importantly, our analysis focuses on the presence of fully assembled complexes and potential assembly intermediates rather than on small differences in band intensity. Consistent with this, no differences in the organization of respiratory chain complexes were observed upon EBS treatment.

**Referee #2 (Comments on Novelty/Model System for Author) :**

The authors have tested the compound of interest in a wide range of human cellular models and yeast models of mitochondrial disease.

Almyre and colleagues have identified Ebselen, an organoselenium compound in phase II and phase III clinical trials for other disorders, with known antioxidant and anti-inflammatory properties that might be a promising candidate for the treatment of mitochondrial dysfunction, given it has shown to be able to cross the blood-brain barrier.

In this manuscript, the authors identified this compound through it's ability to enhance the growth of a yeast model of Barth syndrome, in which cardiolipin remodelling contributes to the pathophysiological disorder associated with the disease phenotype. Ebselen not only enhanced respiratory growth but also improved respiration and O2 consumption in taz1 yeast cells, prompting a deeper analysis in other yeast and fungal models of mitochondrial dysfunction.

The authors propose a novel mechanism of action, involving downregulation of cytosolic translation to restore proteostasis and activation of the TCA cycle via PDH, to stimulate ATP production, suggesting a broad effect of the drug in modulating mitochondrial function. The work is nicely presented and discussed and the paper well written, although as I read through this I am increasingly struggling to look for the key data and conclusions amongst 13 Figures and supplemental data. I have a number of questions and comments for the authors:

1. Why did the authors start the work using a BTHS yeast model, it would be helpful to understand further the path to which this work started and why this model - ahead of say a model with a primary mitochondrial defect - was used?

We thank the reviewer for this important comment. We agree that the rationale for starting with the Barth syndrome (BTHS) model was not sufficiently clear in the original version of the manuscript. To address this point, we have substantially reorganized the Results section. We now first describe the identification and validation of Ebselen (EBS) in yeast and patient-derived cellular models of BTHS, and then show that EBS does not correct the primary molecular defect of the disease, namely the reduction in cardiolipin (CL) abundance. Instead, EBS modifies cardiolipin molecular species while improving mitochondrial function, indicating that its beneficial effects are independent of restoration of the primary lipid defect. This finding provided the rationale for subsequently testing EBS in additional models of mitochondrial disease affecting unrelated mitochondrial pathways, including complex IV biogenesis, mitochondrial DNA maintenance, mitochondrial translation, ATP synthase, and complex I. The consistent efficacy observed across these genetically distinct models demonstrates that EBS is not specific to Barth syndrome or cardiolipin metabolism, but rather acts as a broader modulator of mitochondrial function. We initially selected the BTHS yeast model because it represents one of the best-characterized mitochondrial disease models

available in our laboratory and had previously been successfully used to identify compounds that improve mitochondrial respiration (de Taffin, 2017 ; de Taffin, 2018). Importantly, BTHS also offered a unique translational framework, with complementary yeast, patient-derived cell and mouse models, allowing us to validate the effects of EBS from the initial screen through to *in vivo* studies. We believe that the revised organization of the manuscript now makes this progression and the rationale for extending the study to other mitochondrial diseases much clearer.

2. Ebselen was shown not to increase CL levels in the BTHS yeast model, but treatment was associated with longer-chain species of cardiolipin; what is the proposed mechanism of this? Were experiments also undertaken in other cell models of primary mitochondrial CL deficiency (ie TAMM41 deficiency)?

We thank the reviewer for this comment. In the BTHS yeast model, EBS treatment did not significantly increase total cardiolipin (CL) levels, but was associated with a shift toward longer-chain CL species. Proteomic analysis revealed ACC1, a key enzyme in fatty acid elongation, as a specific EBS interactor, along with a modest increase in other enzymes involved in fatty acid elongation. These findings suggest that EBS may indirectly favor the incorporation of longer-chain fatty acids during CL remodeling, without directly enhancing CL biosynthesis, consistent with a mechanism involving modulation of mitochondrial homeostasis rather than canonical cardiolipin synthesis pathways. We have added the sentences in the discussion of the revised manuscript. Experiments were not performed in other models of primary mitochondrial CL deficiency such as TAMM41-deficient cells, which are beyond the scope of the present study. Our focus on BTHS was motivated by the availability of complementary models, yeast, patient-derived cells, and mice, that allowed a full translational pipeline and robust functional validation.

3. A general question, but the selection of cellular models to exlore the therapeutic effects of Ebselen, does appear (to this reviewer at least) to be rather arbitary......."next we looked at CI deficiency and the ATP synthase deficiency model" - is this simply explained by the availability of models to the laboratory, or is their a further scientific rationale to this selection? What is the cause of the cytotoxicity at concentrations of Ebselen greater than 5uM?

We thank the reviewer for this comment. The selection of cellular models was guided by both scientific rationale and translational relevance. We focused first on the BTHS model, as Tafazzin deficiency directly affects cardiolipin remodeling, which is known to influence the stability and activity of multiple respiratory complexes, including Complex I and ATP synthase. This provides a mechanistic link to the other models we subsequently tested. By examining CI deficiency and ATP synthase deficiency models, we could assess whether EBS has beneficial effects on mitochondrial function beyond cardiolipin defects, targeting conserved pathways across distinct mitochondrial dysfunctions. Importantly, all these models, yeast, patient-derived cells, and mice, have been previously well characterized in our lab, allowing a robust and translational evaluation of EBS efficacy. Cytotoxicity at EBS concentrations above 5 μM likely reflects its pleiotropic nature. At higher doses, EBS can react with multiple thiol-containing proteins and cellular redox systems, potentially disrupting mitochondrial homeostasis and cellular proteostasis. Importantly, the effective concentration range that improved mitochondrial function without inducing toxicity was broad, with robust effects observed well below the cytotoxic threshold. These results indicate that EBS has a favorable therapeutic window across the models tested, consistent with previous reports describing dose-dependent effects of EBS on cellular viability and oxidative stress.

4. I am less convinced, or satisfied, by the n=1 experiment with NDUFV1 iPSC cardiomyocytes - were other lines not available? Did you attempt to rescue any mitochondrial defects in other iPSC cardiomyocyte lines? Page 9 - I do not think that you can say that the drug "improves the structure and mitochondrial defects of cardiomyocytes derived from patients with CI deficiency"

We thank the reviewer for raising this important point. The NDUFV1 iPSC-derived cardiomyocyte experiment was initially performed in a single patient line because well-characterized complex I–deficient iPSC lines were limited in our laboratory at the time. To address this concern, we have now repeated the analysis using an independent clone from the same genetic background, which gave consistent results : after EBS treatment, the increased length-to-width ratio observed in mutant cardiomyocytes was similarly reduced, as in the original clone. These new data are now included in Supplementary Fig. 2. We also agree that the wording needed to be more careful. We have therefore revised the manuscript to make it clear that EBS partially rescues specific mitochondrial and morphological features in this CI-deficient line, rather than broadly stating that it “improves the structure and mitochondrial defects” of cardiomyocytes derived from patients with complex I deficiency.

5. Experiments with the Taz-KO mouse model - I think it would be nice to incorporate some of the data currently in the Supplement in the main Figure (Figure 5) - please consider this

We thank the reviewer for this suggestion. The data currently included in the Supplement primarily show the detailed characterization of the Taz-KO mouse model. To improve clarity, we have incorporated panels from characterization of the BTHS mouse model at the whole animal level into the new Figure 5, allowing readers to better understand the baseline phenotypes.

6. The studies to investigate the effect on Ebselen on OxPhos protein accumulation, stability and assembly are shown in Figure 8 and rely on SDS-PAGE and immunoblotting data; the authors did undertake proteomic analysis, in the treated v untreated mouse tissues, but the manuscript does not really provide a full analysis of the effects at protein level on a whole range of mitochondrial proteins, offering further mechanistic insight. It does appear to be something of a missed opportunity given the access to mouse tissues from the KO......I'm not overly clear from the data in the main paper or the supplement how many proteins were identified or how the analysis was undertaken to identify interactors.

We thank the reviewer for these thoughtful comments regarding the global effects of EBS on the proteome and the identification of its potential interactors. We agree that a more exhaustive proteomic exploration could be of interest. However, in this study, our aim was not to generate an unbiased atlas of all proteomic changes, but rather to identify conserved pathways consistently affected by EBS across yeast, human, and mouse models. Accordingly, we focused our analysis on protein changes that were reproducible across models and that point to shared biological responses, including, but not restricted to, mitochondrial pathways and broader metabolic processes. This cross-species approach was chosen to prioritize conserved functional signatures rather than species-specific proteomic changes. In yeast (Supplementary Fig. 10), 25 proteins were significantly downregulated and 28 upregulated upon EBS treatment (fold change ≥1.5, two unique peptides, statistical significance, and reproducibility criteria applied). These changes reflect a coordinated and moderate proteomic response affecting multiple cellular pathways. Proteins were ranked by T/C ratio and annotated with pathway

information, accession numbers, and peptide counts. Potential interactions with EBS based on cysteine content were indicated (RR = high likelihood, R = moderate likelihood). In the BTHS mouse model (Supplementary Fig. 13), 50 proteins were downregulated and 23 upregulated following EBS treatment using similar filtering criteria (fold change ≥2, detection in at least two of three replicates, and abundance thresholds). As in yeast, the affected proteins were not limited to OXPHOS components but included broader metabolic and cellular pathways, supporting a systems-level response to EBS. Regarding the identification of potential EBS interactors, we applied a stringent affinity-based enrichment strategy across yeast, mouse, and human cells. Proteins were ranked based on competitive binding conditions and filtered using strict criteria (at least two unique peptides, reproducible enrichment across replicates, and significant enrichment ratios). Only the top 30 specifically enriched proteins were retained for downstream analysis. Full methodological details, thresholds, and complete protein lists are provided in the Supplementary Information. Taken together, these analyses provide both a global view of the proteomic response to EBS and a high-confidence set of candidate interactors, highlighting conserved biological pathways affected by treatment across species.

7. Overall, there is a significant body of evidence but the large number of Figures and supplemental data suggest that a simpler message and narrative would help to convince the reader of the mechanism and impact of this work.

We thank the reviewer for this constructive suggestion and fully agree that the original version of the manuscript contained a large amount of data that obscured the main message. We have therefore extensively reorganized the manuscript to provide a clearer and more logical narrative. The Results section now follows a progressive sequence. We first describe the identification of EBS in BTHS yeast and patient-derived cells and establish its beneficial effects on mitochondrial function. We then demonstrate that EBS does not correct the primary cardiolipin defect, providing the rationale for extending the study to additional mitochondrial disease models. Having established that EBS acts broadly across multiple genetic defects, we next present the *in vivo* validation in the BTHS mouse model before moving to the mechanistic studies. The mechanistic section itself has also been substantially reorganized, progressing from the exclusion of previously proposed mechanisms (antioxidant activity, IMP inhibition, cardiolipin restoration, OXPHOS assembly and mitochondrial biogenesis) to the identification of EBS localization, affinity proteomics, multi-omics analyses, and finally the functional validation of the mTOR/translation and pyruvate dehydrogenase complex pathways. We also simplified the presentation by reducing redundancies throughout the manuscript, reorganizing several figures, moving supporting datasets to the Supplementary Information, and rewriting both the Abstract and the Discussion to emphasize the central mechanistic findings. We believe that these changes improve the readability of the manuscript and reinforce the main conclusion that EBS acts as a conserved modulator of mitochondrial function through coordinated regulation of mTOR signalling, protein homeostasis and mitochondrial metabolism.

**Referee #3 (Comments on Novelty/Model System for Author):**

With this research work, a new therapeutical molecule is proven to rescue different mitochondrial defects in different models (yeast, human cells and mice). The authors addressed not only the rescue of different mito-defects but also try to elucidate the mechanism of action. Although some molecular details are still missing, this has high medical potential, as treatment for mito-diseases.

Almyre and co-workers present a sound and well-written study demonstrating that EBS, as a novel therapeutic molecule, is capable of rescuing different mitochondrial defects across multiple models (yeast, human cells, and mice). The authors not only address the rescue of diverse mitochondrial dysfunctions but also attempt to elucidate the mechanism of action. Although some molecular details remain unresolved, this work has strong medical potential, is highly relevant to the field, and positions EBS as a promising therapeutic candidate for mitochondrial diseases. However, several aspects should be addressed prior to publication.

1. In Figure 1, when assessing EBS-mediated rescue across different models of mitochondrial dysfunction, the growth halos do not appear comparable across all systems. The authors could quantify these differences and discuss any potential differential effects of EBS on distinct mitochondrial defects.

We thank the reviewer for this comment. We agree that assessment of growth halos may not fully capture quantitative differences in EBS rescue across models. While precise comparison is challenging due to differences in strain growth dynamics and assay conditions, we have carefully evaluated both activity and toxicity profiles across yeast and fungal models: taz1, sym1, fmc1, atp6: activity and toxicity are broadly comparable. cox2 and shy1: slightly more toxic at higher concentrations, though still showing growth rescue at lower doses. tRNALeu and Pa nuo-51A357V: less toxic and relatively more effective in promoting growth. These observations suggest that while EBS exerts beneficial effects across all models, the magnitude of rescue and the therapeutic window vary depending on the specific mitochondrial defect, likely reflecting differences in metabolic vulnerability and translational regulation in each model. We have added these details to the revised Figure 3 legend and in the results and discussion sections.

2. In Figure 3D, the Seahorse measurements are not clearly presented. It would be helpful to include color-coded graphs or to highlight the most relevant EBS concentration in the main manuscript, with additional data provided in the supplementary information.

We thank the reviewer for this suggestion. We agree that highlighting key concentrations and improving visualization would enhance clarity. In the revised manuscript, we have added color-coded traces for each EBS concentration in updated Figure 3 to facilitate comparison and highlighted the most relevant active concentrations (12 nM to 3μM for BTHS fibroblasts) in the main figure. These changes improve readability while maintaining comprehensive reporting of Seahorse data.

3. It would also be interesting to assess cardiolipin content or lipid distribution following EBS treatment in BTHS cell lines. In addition, and in relation to the reported effects of EBS on translation, what are the TAZ protein levels in patient-derived cells after EBS treatment?

We thank the reviewer for these suggestions. We have performed the requested complementary experiments in patient-derived BTHS cells. Consistent with our observations in yeast, CL levels are decreased in BTHS fibroblasts compared to control cells, and EBS treatment did not restore total CL content under our experimental conditions. These results confirm that the beneficial effects of EBS are independent of total CL restoration. These data have been added to the manuscript in the Results section, panel C in updated Figure 2 and Figure legends. Regarding TAZ protein expression, we attempted to assess endogenous TAZ

levels in patient-derived cells. Unfortunately, we were unable to identify an antibody that produced a specific and reproducible signal, precluding a reliable analysis of TAZ abundance. Nevertheless, because EBS failed to restore cardiolipin levels in patient cells, our data strongly indicate that the compound does not rescue tafazzin function or correct the primary molecular defect underlying Barth syndrome.

4. Regarding mutant cardiomyocytes treated with EBS, are there functional parameters (e.g., beating rate, $Ca^{2+}$ signaling, ATP production, or similar readouts) that are impaired in patient cells and can be restored upon treatment?

We thank the reviewer for this valuable suggestion. In response, we have performed additional functional analyses in patient-derived iPSC cardiomyocytes. Beyond the structural improvements initially reported, EBS treatment significantly improved several key functional parameters, including contractility, and calcium handling. In particular, EBS restored the reduced contraction and relaxation amplitudes observed in mutant cardiomyocytes and improved calcium transient kinetics. These functional improvements complement the morphological rescue and further support the beneficial effects of EBS on mitochondrial and cardiac function. These new data have been incorporated into the revised manuscript (Results section, Fig. 4 and Supplementary Fig. 2).

5. The results presented in Figure 8 appear somewhat surprising. First, in yeast models, there is an apparent increase in Cox2 upon EBS treatment based on both the Western blot (taz1 and fmc1) and quantifications (taz1), which is not discussed by the authors.

We thank the reviewer for this comment. After normalization to a loading control, Cox2 levels in the yeast models do not show a significant increase following EBS treatment. The apparent increase observed in the Western blot images is likely due to variability in sample loading rather than a true effect

6. In addition, the Western blot panel for BTHS fibroblasts would benefit from the inclusion of a control cell line, as in the other experiments.

We thank the reviewer for this suggestion. We have now repeated the experiments including a control cell line analysed in parallel with BTHS fibroblasts in newly performed experiments. The corresponding control has now been added to the Western blot panel in the revised manuscript, in line with the other experiments. The figure legend has also been updated accordingly.

7. If EBS does not affect OXPHOS assembly, this could potentially not explain the observed increases in OCR and growth. What alternative hypotheses do the authors consider? One possible explanation is the use of different sample sources across experiments: OCR measurements are performed in whole cells, whereas Western blots are likely derived from isolated mitochondria. An increase in mitochondrial mass per cell could account for the observed rescue of respiration, while OXPHOS subunit levels per mitochondrion (as assessed by Western blot) may remain unchanged. The authors could investigate whether mitochondrial mass is increased, potentially as a result of mitochondrial biogenesis.

We thank the reviewer for this insightful comment. We agree that the increase in oxygen consumption rate (OCR) and growth, despite the absence of changes in OXPHOS protein abundance or assembly, requires mechanistic clarification. In whole-cell assays, EBS enhances respiratory function without increasing OXPHOS complex levels per mitochondrion. We propose that this apparent discrepancy reflects a functional rather than structural

improvement of mitochondrial metabolism. Specifically, EBS stimulates pyruvate metabolism and TCA cycle activity, thereby increasing the supply of reducing equivalents (NADH/FADH2) to the respiratory chain. This results in enhanced electron flux and OCR without requiring an increase in respiratory complex abundance or mitochondrial biogenesis. To address the possibility that this effect could be due to increased mitochondrial content, we examined markers of mitochondrial biogenesis and mitochondrial mass. TFAM expression was found to be elevated in the BTHS model compared to wild-type, consistent with a compensatory response to mitochondrial dysfunction, but was not further increased by EBS treatment. These data indicate that EBS does not induce mitochondrial biogenesis. Taken together, these results support a model in which EBS improves mitochondrial function primarily by enhancing metabolic flux through the TCA cycle and upstream pathways, rather than by increasing mitochondrial mass or OXPHOS assembly. This functional improvement is sufficient to increase electron transport activity and cellular respiration in whole cells. These data have been incorporated into the revised manuscript.

EBS appears to interact with multiple cellular targets and regulate major pathways that may contribute to improved mitochondrial function. However, beyond its reported effects on the PDC complex and the TCA cycle, the connection between EBS and mitochondrial rescue remains unclear.

8. A critical aspect of this study would be to better link EBS-mediated modulation of mTOR to cytosolic translation inhibition and the potential enhancement of mitochondrial respiration and cellular growth, possibly through mitochondrial biogenesis. Have the authors considered the involvement of the mt-UPR/ISR pathway in relation to mTOR signaling?

We thank the reviewer for this insightful comment. We agree that understanding how EBS links mTOR signalling to cytosolic translation and mitochondrial function is central to this study. In response, we have substantially strengthened both the Results and Discussion to better integrate these observations into a coherent mechanistic model. First, we now provide functional evidence supporting the involvement of mTOR signalling. We show that phosphorylation of ribosomal protein S6 (p-RPS6), a canonical downstream readout of mTOR activity, is reduced in the BTHS mouse heart and restored following EBS treatment. Conversely, pharmacological inhibition of TOR with rapamycin markedly attenuates the respiratory growth rescue induced by EBS in the BTHS yeast model. Together, these findings support a functional requirement for mTOR signalling in the response to EBS (new Section 12). Second, we directly assessed cytosolic protein synthesis and found that EBS does not inhibit translation in a cell-free system but induces a moderate (25%) reduction of cytosolic translation *in vivo* in two independent yeast models of mitochondrial disease (*taz1* and *fmc1*). This effect is accompanied by decreased levels of the proteostatic stress markers HSP70 and CHOP in the BTHS mouse model, consistent with improved protein homeostasis rather than direct inhibition of the translational machinery. Third, we further investigated mitochondrial metabolism. We show that EBS downregulates PDK4 while increasing PDP1, two reciprocal regulators of the pyruvate dehydrogenase complex (PDC), thereby favouring its active state and promoting carbon entry into the tricarboxylic acid (TCA) cycle. In addition, pharmacological activation of PDC using dichloroacetate or acetate partially phenocopies the beneficial effects of EBS in the yeast model, supporting the contribution of metabolic reprogramming to the rescue mechanism. Regarding mitochondrial biogenesis, we specifically addressed this possibility in the revised manuscript. TFAM protein abundance, used as a marker of mitochondrial biogenesis, was not increased by EBS treatment in the BTHS mouse model (new Fig. 8), arguing against enhanced mitochondrial biogenesis as the primary mechanism underlying the improvement in mitochondrial function.

Finally, in the revised manuscript we examined the expression of the stress markers CHOP and HSP70. Both were elevated in the BTHS mouse model and decreased following EBS treatment, indicating an attenuation of proteostatic stress. We have incorporated these observations into the revised Discussion, where we propose that EBS engages lysosome-associated mTOR signalling to coordinately modulate cytosolic translation and mitochondrial metabolism. While additional work will be required to define the precise molecular links between mTOR, PDC regulation and cellular stress pathways, our current data support a model in which EBS improves mitochondrial function through coordinated regulation of protein homeostasis and bioenergetic metabolism rather than through induction of mitochondrial biogenesis.

13th Aug 2026

Dear Dr. Tribouillard-Tanvier,

Thank you for the submission of your manuscript to EMBO Molecular Medicine. We have now received feedback from the two reviewers who agreed to evaluate your manuscript. As you will see from their reports pasted below both referees support publication of your manuscript. Therefore, I am pleased to inform you that we will be able to accept your manuscript pending the following final amendments:

1) Figures:
- Please rationalize the number of figures. Of the current 15 main figures half should remain as main figures and half should be uploaded as Expanded View (EV) Figures. We also note that some figures have only 2-3 panels and could be merged. Main and EV Figures should be uploaded as separate high-resolution files. Please check "Author Guidelines" for more information:
https://link.springer.com/journal/44321/submission-guidelines#cms-Figure-and-data-presentation
https://link.springer.com/journal/44321/submission-guidelines#cms-Expanded-View-data
- We note that some panels might be reused e.g. Figure 6E and Appendix Fig S3F, Figure 8F and Appendix Fig S3G, Figure 13D and Figure 14A. Please cite in the respective figure legend every reused panel.
2) Source Data: Please upload source data for the main figures as one zipped file per figure.
3) Title: Please consider revising the title to better emphasize the main findings, e.g. "Ebselen restores mitochondrial function through metabolic rewiring in Barth syndrome and related mitochondrial diseases"
4) Abstract: I have gone through your text and revised it (see below). Please review it for accuracy and amend as you see fit:

Despite major advances in the genetic diagnosis of mitochondrial disorders, effective disease-modifying therapies remain scarce. Here, we identify ebselen (EBS) as a promising therapeutic candidate through a phenotypic drug screen in a yeast model of Barth syndrome, a cardiomyopathy caused by defective cardiolipin maturation. EBS improved oxidative phosphorylation-dependent growth across diverse fungal models of mitochondrial diseases, including defects in complex I, complex IV, mitochondrial DNA maintenance, mitochondrial translation, and ATP synthase. Therapeutic efficacy was further validated in patient-derived fibroblasts, iPSC-derived cardiomyocytes, and a cardiolipin-deficient mouse model. Mechanistically, EBS acts independently of its established antioxidant activity by engaging a conserved metabolic program that suppresses cytosolic translation while stimulating pyruvate dehydrogenase-dependent tricarboxylic acid cycle activity, thereby improving proteostasis and mitochondrial bioenergetic function. These effects are likely coordinated through lysosome-associated mTOR signaling, consistent with the localization of EBS to lysosomes and its colocalization with mTOR. Together, our findings identify a conserved mechanism for enhancing mitochondrial function and establish ebselen as a strong candidate for therapeutic repurposing in Barth syndrome and a broad spectrum of inherited mitochondrial disorders.

5) In the main manuscript file, please do the following:
- Please address all comments suggested by our data editors listed below:
o Figure legends:
1. Please note that the legends for figure 8 are not provided in a sequential manner (legend for figure 8D is provided before legend of figure 8C). This needs to be rectified.
2. Please define the annotated p values */***/**** as well as provide the exact p-values for the same in the legend of figures 1F; 2A; 6D; 8A; 13B; as appropriate.
3. Please note that the exact p values are not provided in the legends of figures 1B-E; 2B,C; 3C; 4A',B',C,D; 5B-F; 6A-C; 7A-C; 9A; 13C,D; 14A.
4. Please indicate the statistical test used for data analysis in the legends of figure 1B-E; 2B,C; 3C; 4A',B',C,D; 5B-F; 6A-C; 7A-C; 8B,D,E; 9A; 13C,D; 14A.
5. Please note that information related to n is missing in the legends of figures 2A; 4B'.
6. Please note that the error bars are not defined in the legends of figures 1B-F; 2A.
- In the authors list both first and last name should be spelled out.
- Please ensure that all figures and panels are cited in sequential order.
- In Methods, statistical paragraph should reflect all information that you have filled in the Authors Checklist, especially regarding randomization, blinding, replication etc.
- Indicate in legends exact n and exact p values, not a range, along with the statistical test used. To keep the figures "clear" some authors found providing an Appendix table Sx with all exact p-values preferable. You are welcome to do this if you want to.
- Please include structured Methods section that includes a Reagents and Tools Table (should be uploaded as a separate file) followed by a Methods and Protocols section. More information on how to adhere to this format as well as downloadable templates (.docx) for the Reagents and Tools Table can be found in our author guidelines:
https://www.embopress.org/page/journal/17574684/authorguide#structuredmethods
An example of a paper with Structured Methods can be found here:
https://www.embopress.org/doi/full/10.1038/s44320-024-00037-6#sec-4
- Rename "Competing interest" to "Disclosure Statement & Competing Interests". We updated our journal's competing interests policy in January 2022 and request authors to consider both actual and perceived competing interests. Please review the policy

https://www.embopress.org/competing-interests and update your competing interests if necessary.
- Author contributions: Please remove it from the manuscript and specify author contributions in our submission system. CRediT has replaced the traditional author contributions section because it offers a systematic machine-readable author contributions format that allows for more effective research assessment. You are encouraged to use the free text boxes beneath each contributing author's name to add specific details on the author's contribution. More information is available in our guide to authors:
https://www.embopress.org/page/journal/17574684/authorguide#authorshipguidelines
- Correct the reference citation in the text and reference list. In the text a reference should be cited by author and year of publication. Include a space between a word and the opening parenthesis of the reference that follows. In the reference list, citations should be listed in alphabetical order. Where there are more than 10 authors on a paper, 10 will be listed, followed by "et al.". Also, please remove DOIs. Please check "Author Guidelines" for more information.
https://link.springer.com/journal/44321/submission-guidelines#cms-Reference-guidelines
6) Funding: Merge it with "Acknowledgments" and ensure that all funders are listed in our submission system.
7) Movies: Please rename movie files to Movie EV1 etc. and zip each file with the corresponding legend that should be provided as a readme.txt file. Please update their callouts in main manuscript file.
8) Appendix:
- Please rename supplementary file to "Appendix" and move all supplementary methods to the main Methods section. "Compound Synthesis" should remain in the Appendix under the heading "Appendix Supplementary Information" and should be cited in the main manuscript text.
- Appendix should be uploaded as a PDF with a table of contents and page numbers on the title page.
- Rename suppl. figures 1, 2,3,5,6 and 11 to "Appendix Figure S1" etc. and update their callouts in the main manuscript file.
- Suppl. Figs 4, 7, 8,9,10 should be uploaded as individual table files and renamed to Table EV1 etc., with the legends added to each corresponding table. Please update their callouts in the main manuscript file.
9) The Paper Explained: Please provide "The Paper Explained" and add it to the main manuscript text. Please check any of recently published EMBO Mol Med research articles for reference.
10) Synopsis: Every published paper now includes a 'Synopsis' to further enhance discoverability. Synopses are displayed on the journal webpage and are freely accessible to all readers. They include separate synopsis image and synopsis text.
- Synopsis image: Please resize the image to exactly 550 px-wide x 300 pixels high and use larger font for the text where appropriate. Upload it as a high resolution .jpeg/.png file.
- Synopsis text: Please provide a short standfirst (maximum of 300 characters, including space) as well as 2-5 one sentence bullet points that summarize the paper as a .doc file. Please write the bullet points to summarize the key NEW findings. They should be designed to be complementary to the abstract - i.e. not repeat the same text. We encourage inclusion of key acronyms and quantitative information (maximum of 30 words / bullet point). Please use the passive voice.
- Please check your synopsis text and image before submission with your revised manuscript. Please be aware that in the proof stage minor corrections only are allowed (e.g., typos).
11) As part of the EMBO Publications transparent editorial process (see our Editorial at http://embomolmed.embopress.org/content/2/9/329), EMBO Molecular Medicine will publish online a Review Process File (RPF) to accompany accepted manuscripts. This file will be published in conjunction with your paper and will include the anonymous referee reports, your point-by-point response and all pertinent correspondence relating to the manuscript. Let us know if you want to remove or not any figures from it prior to publication. Please note that the Authors checklist will be published at the end of the RPF.
12) Please provide a point-by-point letter INCLUDING my comments as well as the reviewer's reports and your detailed responses (as Word file).

I look forward to reading a new revised version of your manuscript as soon as possible.

Yours sincerely,

Zeljko Durdevic

Zeljko Durdevic
Senior Editor
EMBO Molecular Medicine

*** Instructions to submit your revised manuscript ***

*** PLEASE NOTE *** As part of the EMBO Publications transparent editorial process initiative (see our Editorial at https://www.embopress.org/doi/pdf/10.1002/emmm.201000094), EMBO Molecular Medicine will publish online a Review Process File to accompany accepted manuscripts.

In the event of acceptance, this file will be published in conjunction with your paper and will include the anonymous referee reports, your point-by-point response and all pertinent correspondence relating to the manuscript. If you do NOT want this file to be published, please inform the editorial office at contact@embomolmed.org.

*****

When preparing your revised manuscript, please refer to our guidelines: https://link.springer.com/journal/44321/submission-guidelines#cms-Revised-submissions. We perform an initial quality control of all revised manuscripts before re-review; failure to include requested items will delay the evaluation of your revision.

We require:

1) A .docx formatted version of the manuscript text (including legends for main figures, EV figures and tables). Please make sure that the changes are highlighted to be clearly visible.

2) Individual production quality figure files as .eps, .tif, .jpg (one file per figure). For guidance, download the 'Figure Guide PDF': https://media.springernature.com/original/springer-cms/rest/v1/content/27825798/data/v1.

3) A .docx formatted letter INCLUDING the reviewers' reports and your detailed point-by-point responses to their comments. As part of the EMBO Press transparent editorial process, the point-by-point response is part of the Review Process File (RPF), which will be published alongside your paper.

4) A complete author checklist, which you can download from our author guidelines. Please insert information in the checklist that is also reflected in the manuscript. The completed author checklist will also be part of the RPF.

5) Please note that all corresponding authors are required to supply an ORCID ID for their name upon submission of a revised manuscript.

6) It is mandatory to include a 'Data Availability' section after the Materials and Methods. Before submitting your revision, primary datasets produced in this study need to be deposited in an appropriate public database, and the accession numbers and database listed under 'Data Availability'. Please remember to provide a reviewer password if the datasets are not yet public.

In case you have no data that requires deposition in a public database, please state so in this section. Note that the Data Availability Section is restricted to new primary data that are part of this study.

7) For data quantification: please specify the name of the statistical test used to generate error bars and P values, the number (n) of independent experiments (specify technical or biological replicates) underlying each data point and the test used to calculate p-values in each figure legend. The figure legends should contain a basic description of n, P and the test applied. Graphs must include a description of the bars and the error bars (s.d., s.e.m.).

8) At EMBO Press we ask authors to provide source data for the main manuscript figures. You will receive a separate email with instructions for providing source data with your revised manuscript, including how to upload and organize the files.

9) Our journal encourages inclusion of *data citations in the reference list* to directly cite datasets that were re-used and obtained from public databases. Data citations in the article text are distinct from normal bibliographical citations and should directly link to the database records from which the data can be accessed. In the main text, data citations are formatted as follows: "Data ref: Smith et al, 2001" or "Data ref: NCBI Sequence Read Archive PRJNA342805, 2017". In the Reference list, data citations must be labeled with "[DATASET]". A data reference must provide the database name, accession number/identifiers and a resolvable link to the landing page from which the data can be accessed at the end of the reference.

10) We replaced Supplementary Information with Expanded View (EV) Figures and Tables that are collapsible/expandable online. A maximum of 5 EV Figures can be typeset. EV Figures should be cited as 'Figure EV1, Figure EV2" etc... in the text and their respective legends should be included in the main text after the legends of regular figures.

- For the figures that you do NOT wish to display as Expanded View figures, they should be bundled together with their legends in a single PDF file called *Appendix*, which should start with a short Table of Content. Appendix figures should be referred to in the main text as: "Appendix Figure S1, Appendix Figure S2" etc.

- Additional Tables/Datasets should be labeled and referred to as Table EV1, Dataset EV1, etc. Legends have to be provided in a separate tab in case of .xls files. Alternatively, the legend can be supplied as a separate text file (README) and zipped together with the Table/Dataset file.

11) The paper explained: EMBO Molecular Medicine articles are accompanied by a summary of the articles to emphasize the major findings in the paper and their medical implications for the non-specialist reader. Please provide a draft summary of your article highlighting

- the medical issue you are addressing,

- the results obtained and

- their clinical impact.

This may be edited to ensure that readers understand the significance and context of the research. Please refer to any of our published articles for an example.

12) Author contributions: You will be asked to provide CRediT (Contributor Role Taxonomy) terms in the submission system. These replace a narrative author contribution section in the manuscript.

13) A Conflict of Interest statement should be provided in the main text.

14) Every published paper includes a 'Synopsis' to further enhance discoverability. Synopses are displayed on the journal webpage and are freely accessible to all readers. They include a short stand first (maximum of 300 characters, including space) as well as 2-5 one-sentences bullet points that summarizes the paper. Please write the bullet points to summarize the key NEW findings. They should be designed to be complementary to the abstract - i.e. not repeat the same text. We encourage inclusion of key acronyms and quantitative information (maximum of 30 words / bullet point). Please use the passive voice. Please attach these in a separate file or send them by email, we will incorporate them accordingly.

Please also suggest a striking image or visual abstract to illustrate your article as a PNG file 550 px wide x 300 px high.

15) Include a Reagents and Tools Table as part of the Methods section, which can be downloaded from our author guidelines.

EMBO Molecular Medicine has a "scooping protection" policy, whereby similar findings that are published by others during review or revision are not a criterion for rejection. Should you decide to submit a revised version, I do ask that you get in touch after three months if you have not completed it, to update us on the status.

*Additional important information regarding Figures

Each figure should be given in a separate file and should have the following resolution:
Graphs 800-1,200 DPI
Photos 400-800 DPI
Colour (only CMYK) 300-400 DPI"

Figures are not edited by the production team. All lettering should be the same size and style; figure panels should be indicated by capital letters (A, B, C etc). Gridlines are not allowed except for log plots. Figures should be numbered in the order of their appearance in the text with Arabic numerals. Each Figure must have a separate legend and a caption is needed for each panel.

*Additional important information regarding figures and illustrations can be found at
https://media.springernature.com/original/springer-cms/rest/v1/content/27825798/data/v1

***** Reviewer's comments *****

Referee #1 (Remarks for Author):

The authors have addressed my previous concerns.

Referee #2 (Comments on Novelty/Model System for Author):

This is a revision, I would have commented previously but feel that the choice of the model systems used is both appropriate and wide-ranging.

Referee #2 (Remarks for Author):

The authors have appropriately responded to my comments and revised the manuscript accordingly - I have no further comments. Specifically, I asked for the narrative to be simplified to present a clearer, more logical story and I think the authors have responded to this request in full; thank you.

The authors addressed the remaining editorial issues.

21st Aug 2026

Dear Dr. Tribouillard-Tanvier,

We are pleased to inform you that your manuscript is accepted for publication and is now being sent to our publisher to be included in the next available issue of EMBO Molecular Medicine.

Your manuscript will be processed for publication by EMBO Press. It will be copy edited and you will receive page proofs prior to publication. Please note that you will be contacted by Springer Nature Author Services to complete licensing and payment information.

You may qualify for financial assistance for your publication charges - either via a Springer Nature fully open access agreement or an EMBO initiative. Check your eligibility: https://link.springer.com/journal/44321/how-to-publish-with-us

Should you be planning a Press Release on your article, please notify the production team via this form in order to coordinate publication and release dates: https://forms.cloud.microsoft/e/eH4A8JVP6g

If you have any questions, please do not hesitate to contact the Editorial Office. Thank you for your contribution to EMBO Molecular Medicine.

Yours sincerely,
Zeljko Durdevic

Zeljko Durdevic
Senior Editor
EMBO Molecular Medicine

------------------------------------------------

>>> Please note that it is EMBO Molecular Medicine policy for the transcript of the editorial process (containing referee reports and your response letter) to be published as an online supplement to each paper. If you do NOT want this, you will need to inform the Editorial Office via email immediately. More information is available here: https://link.springer.com/partners/embo-press/editorial-policies#Peer%20review

## EMBO Press Author Checklist


| |
|---|
| Corresponding Author Name: TRIBOUILLARD-TANVIER Deborah |
| Journal Submitted to: EMBO Molecular Medicine |
| Manuscript Number: EMM-2026-23567-V2 |


**USEFUL LINKS FOR COMPLETING THIS FORM**
The EMBO Journal - Author Guidelines
EMBO Reports - Author Guidelines
Molecular Systems Biology - Author Guidelines
EMBO Molecular Medicine - Author Guidelines

**Reporting Checklist for Life Science Articles (updated January**
This checklist is adapted from Materials Design Analysis Reporting (MDAR) Checklist for Authors. MDAR establishes a minimum set of requirements in transparent reporting in the life sciences (see Statement of Task: 10.31222/osf.io/9sm4x). Please follow the journal's guidelines in preparing your
**Please note that a copy of this checklist will be published alongside your article.**

**Abridged guidelines for figures**
**1. Data**
The data shown in figures should satisfy the following conditions:
- the data were obtained and processed according to the field's best practice and are presented to reflect the results of the experiments in an accurate and unbiased manner.
- ideally, figure panels should include only measurements that are directly comparable to each other and obtained with the same assay.
- plots include clearly labeled error bars for independent experiments and sample sizes. Unless justified, error bars should not be shown for technical
- if n<5, the individual data points from each experiment should be plotted. Any statistical test employed should be justified.
- Source Data should be included to report the data underlying figures according to the guidelines set out in the authorship guidelines on Data

**2. Captions**
Each figure caption should contain the following information, for each panel where they are relevant:
- a specification of the experimental system investigated (eg cell line, species name).
- the assay(s) and method(s) used to carry out the reported observations and measurements.
- an explicit mention of the biological and chemical entity(ies) that are being measured.
- an explicit mention of the biological and chemical entity(ies) that are altered/varied/perturbed in a controlled manner.
- the exact sample size (n) for each experimental group/condition, given as a number, not a range;
- a description of the sample collection allowing the reader to understand whether the samples represent technical or biological replicates (including how many animals, litters, cultures, etc.).
- a statement of how many times the experiment shown was independently replicated in the laboratory.
- definitions of statistical methods and measures:
  - common tests, such as t-test (please specify whether paired vs. unpaired), simple χ2 tests, Wilcoxon and Mann-Whitney tests, can be unambiguously identified by name only, but more complex techniques should be described in the methods section;
  - are tests one-sided or two-sided?
  - are there adjustments for multiple comparisons?
  - exact statistical test results, e.g., P values = x but not P values < x;
  - definition of 'center values' as median or average;
  - definition of error bars as s.d. or s.e.m.

**Please complete ALL of the questions below.**
**Select "Not Applicable" only when the requested information is not relevant for your study.**

**Materials**

| Newly Created Materials | Information included in the manuscript? | In which section is the information available? (Reagents and Tools Table, Materials and Methods, Figures, Data Availability Section) |
|---|---|---|
| New materials and reagents need to be available; do any restrictions apply? | Yes | Reagents and tools table, Material and methods |

| Antibodies | Information included in the manuscript? | In which section is the information available? (Reagents and Tools Table, Materials and Methods, Figures, Data Availability Section) |
|---|---|---|
| For **antibodies** provide the following information:<br>- Commercial antibodies: RRID (if possible) or supplier name, catalogue number and or/clone number<br>- Non-commercial: RRID or citation | Yes | Reagents and tools table, Material and methods |

| DNA and RNA sequences | Information included in the manuscript? | In which section is the information available? (Reagents and Tools Table, Materials and Methods, Figures, Data Availability Section) |
|---|---|---|
| **Short novel DNA or RNA including primers, probes**: provide the sequences. | Not Applicable | |

| Cell materials | Information included in the manuscript? | In which section is the information available? (Reagents and Tools Table, Materials and Methods, Figures, Data Availability Section) |
|---|---|---|
| **Cell lines:** Provide species information, strain. Provide accession number in repository **OR** supplier name, catalog number, clone number, and/**OR** RRID. | Yes | Reagents and tools table, Material and methods |
| **Primary cultures:** Provide species, strain, sex of origin, genetic modification status. | Yes | Reagents and tools table, Material and methods |
| Report if the cell lines were recently **authenticated** (e.g., by STR profiling) and tested for mycoplasma contamination. | Yes | Reagents and tools table, Material and methods |

| Experimental animals | Information included in the manuscript? | In which section is the information available? (Reagents and Tools Table, Materials and Methods, Figures, Data Availability Section) |
|---|---|---|
| **Laboratory animals or Model organisms:** Provide species, strain, sex, age, genetic modification status. Provide accession number in repository **OR** supplier name, catalog number, clone number, **OR** RRID. | Yes | Reagents and tools table, Material and methods |
| **Animal observed in or captured from the field:** Provide species, sex, and age where possible. | Not Applicable | |
| Please detail **housing and husbandry conditions**. | Yes | Material and methods |

| Plants and microbes | Information included in the manuscript? | In which section is the information available? (Reagents and Tools Table, Materials and Methods, Figures, Data Availability Section) |
|---|---|---|
| **Plants:** provide species and strain, ecotype and cultivar where relevant, unique accession number if available, and source (including location for collected wild specimens). | Not Applicable | |
| **Microbes:** provide species and strain, unique accession number if available, and source. | Not Applicable | |

| Human research participants | Information included in the manuscript? | In which section is the information available? (Reagents and Tools Table, Materials and Methods, Figures, Data Availability Section) |
|---|---|---|
| If collected and within the bounds of privacy constraints report on age, sex and gender or ethnicity for all study participants. | Not Applicable | |

| Core facilities | Information included in the manuscript? | In which section is the information available? (Reagents and Tools Table, Materials and Methods, Figures, Data Availability Section) |
|---|---|---|
| If your work benefited from core facilities, was their service mentioned in the acknowledgments section? | Yes | Acknowledgments section |

**Design**

| Study protocol | Information included in the manuscript? | In which section is the information available? (Reagents and Tools Table, Materials and Methods, Figures, Data Availability Section) |
|---|---|---|
| If study protocol has been **pre-registered, provide DOI in the manuscript**. For clinical trials, provide the trial registration number **OR** cite DOI. | Not Applicable | |
| Report the **clinical trial registration number** (at ClinicalTrials.gov or equivalent), where applicable. | Not Applicable | |

| Laboratory protocol | Information included in the manuscript? | In which section is the information available? (Reagents and Tools Table, Materials and Methods, Figures, Data Availability Section) |
|---|---|---|
| Provide DOI OR other citation details if **external detailed step-by-step protocols** are available. | Yes | Material and methods |

| Experimental study design and statistics | Information included in the manuscript? | In which section is the information available? (Reagents and Tools Table, Materials and Methods, Figures, Data Availability Section) |
|---|---|---|
| Include a statement about **sample size** estimate even if no statistical methods were used. | Yes | Figure legends |
| Were any steps taken to minimize the effects of subjective bias when allocating animals/samples to treatment (e.g. **randomization procedure**)? If yes, have they been described? | Yes | Material and methods |
| Include a statement about **blinding** even if no blinding was done. | Yes | Material and methods |
| Describe **inclusion/exclusion criteria** if samples or animals were excluded from the analysis. Were the criteria pre-established?<br><br>If sample or data points were omitted from analysis, report if this was due to attrition or intentional exclusion and provide justification. | Yes | Material and methods |
| For every figure, are **statistical tests** justified as appropriate? Do the data meet the assumptions of the tests (e.g., normal distribution)? Describe any methods used to assess it. Is there an estimate of variation within each group of data? Is the variance similar between the groups that are being statistically compared? | Yes | Material and methods, Figure legends and Appendix table 1 |

| Sample definition and in-laboratory replication | Information included in the manuscript? | In which section is the information available? (Reagents and Tools Table, Materials and Methods, Figures, Data Availability Section) |
|---|---|---|
| In the figure legends: state number of times the experiment was **replicated** in laboratory. | Yes | Figure legends |
| In the figure legends: define whether data describe **technical or biological replicates**. | Yes | Figure legends |

## Ethics

| Ethics | Information included in the manuscript? | In which section is the information available? (Reagents and Tools Table, Materials and Methods, Figures, Data Availability Section) |
|---|---|---|
| Studies involving **human participants**: State details of **authority granting ethics approval** (IRB or equivalent committee(s), provide reference number for approval. | Not Applicable | |
| Studies involving **human participants**: Include a statement confirming that **informed consent** was obtained from all subjects and that the experiments conformed to the principles set out in the WMA Declaration of Helsinki and the Department of Health and Human Services Belmont Report. | Not Applicable | |
| Studies involving **human participants: For publication of patient photos**, include a statement confirming that consent to publish was obtained. | Not Applicable | |
| Studies involving experimental **animals**: State details of **authority granting ethics approval** (IRB or equivalent committee(s), provide reference number for approval. Include a statement of compliance with ethical regulations. | Yes | Material and methods |
| Studies involving **specimen and field samples:** State if relevant **permits** obtained, provide details of authority approving study; if none were required, explain why. | Not Applicable | |

| Dual Use Research of Concern (DURC) | Information included in the manuscript? | In which section is the information available? (Reagents and Tools Table, Materials and Methods, Figures, Data Availability Section) |
|---|---|---|
| Could your study fall under dual use research restrictions? Please check biosecurity documents and list of **select agents and toxins** (CDC): https://www.selectagents.gov/sat/list.htm | Not Applicable | |
| If you used a select agent, is the security level of the lab appropriate and reported in the manuscript? | Not Applicable | |
| If a study is subject to dual use research of concern regulations, is the name of the **authority granting approval and reference number** for the regulatory approval provided in the manuscript? | Not Applicable | |

## Reporting

The MDAR framework recommends adoption of discipline-specific guidelines, established and endorsed through community initiatives. Journals have their own policy about requiring specific guidelines and recommendations to complement MDAR.

| Adherence to community standards | Information included in the manuscript? | In which section is the information available? (Reagents and Tools Table, Materials and Methods, Figures, Data Availability Section) |
|---|---|---|
| State if relevant guidelines or checklists (e.g., **ICMJE, MIBBI, ARRIVE, PRISMA**) have been followed or provided. | Yes | Material and methods |
| For **tumor marker prognostic studies,** we recommend that you follow the **REMARK** reporting guidelines (see link list at top right). See author guidelines, under 'Reporting Guidelines'. Please confirm you have followed these guidelines. | Not Applicable | |
| For **phase II and III randomized controlled trials**, please refer to the **CONSORT** flow diagram (see link list at top right) and submit the CONSORT checklist (see link list at top right) with your submission. See author guidelines, under 'Reporting Guidelines'. Please confirm you have submitted this list. | Not Applicable | |

## Data Availability

| Data availability | Information included in the manuscript? | In which section is the information available? (Reagents and Tools Table, Materials and Methods, Figures, Data Availability Section) |
|---|---|---|
| Have **primary datasets** been deposited according to the journal's guidelines (see 'Data Deposition' section) and the respective accession numbers provided in the Data Availability Section? | Yes | Data availability |
| Were **human clinical and genomic datasets** deposited in a public access-controlled repository in accordance to ethical obligations to the patients and to the applicable consent agreement? | Not Applicable | |
| Are **computational models** that are central and integral to a study available without restrictions in a machine-readable form? Were the relevant accession numbers or links provided? | Not Applicable | |
| If publicly available data were reused, provide the respective **data citations in the reference list**. | Not Applicable | |
