# Supplementary material for: Ebselen restores mitochondrial function through metabolic rewiring in Barth syndrome and related mitochondrial diseases
Source: EMBO Mol Med. 2026 Oct 5;18(11):4227–59. doi: 10.1038/s44321-026-00518-1 (PMC13748542; doi:10.1038/s44321-026-00518-1)
Supplement: Supplementary file 5 [file 44321_2026_518_MOESM11_ESM.zip › Movie EV3 readme.txt.docx]

**Movie EV3 - WT + SLV**

**Movie EV3. Three-dimensional visualization of cardiac mitochondria in a solvent-treated WT mouse heart.** Representative serial block-face scanning electron microscopy (SBF-SEM) dataset of a WT mouse heart treated with solvent (SLV). Individual mitochondria are shown in color, while non-colored regions correspond to cardiac muscle fibers. The movie provides a three-dimensional visualization of mitochondrial organization within the cardiac tissue.
