# Supplementary material for: Ebselen restores mitochondrial function through metabolic rewiring in Barth syndrome and related mitochondrial diseases
Source: EMBO Mol Med. 2026 Oct 5;18(11):4227–59. doi: 10.1038/s44321-026-00518-1 (PMC13748542; doi:10.1038/s44321-026-00518-1)
Supplement: Supplementary file 6 [file 44321_2026_518_MOESM12_ESM.zip › Movie EV4 readme.txt.docx]

**Movie EV4 - BTHS + SLV**

**Three-dimensional visualization of cardiac mitochondria in a solvent-treated BTHS mouse heart.** Representative SBF-SEM dataset of a Taz-KO (BTHS) mouse heart treated with solvent (SLV). Individual mitochondria are shown in color, whereas non-colored regions correspond to cardiac muscle fibers.
