# Supplementary material for: Ebselen restores mitochondrial function through metabolic rewiring in Barth syndrome and related mitochondrial diseases
Source: EMBO Mol Med. 2026 Oct 5;18(11):4227–59. doi: 10.1038/s44321-026-00518-1 (PMC13748542; doi:10.1038/s44321-026-00518-1)
Supplement: Supplementary file 14 [file 44321_2026_518_MOESM1_ESM.docx]

**Table EV1. Pharmacokinetic analysis of EBS in WT mice**

**A Plasma EBS concentrations after a single oral dose of 10 mg/kg**

| **Time (h)** | **Sample concentration (ng/mL)** | | | **Mean** | **SD** |
| --- | --- | --- | --- | --- | --- |
|  | **Mouse #1** | **Mouse #2** | **Mouse #3** |  |  |
| 0.083 | 678 | 915 | 158 | 584 | 387 |
| 0.25 | 384 | 716 | 131 | 410 | 293 |
| 0.5 | 210 | 342 | 64 | 205 | 139 |
| 1 | 90 | 97 | 72 | 86 | 13 |
| 2 | 24 | 55 | 46 | 42 | 16 |
| 4 | 12 | 10 | 7 | 10 | 3 |
| 8 | 7 | BLOQ | 5 | 6 | 1 |

**B Plasma pharmacokinetic parameters after a single oral dose of 10 mg/kg**

| **10 mg/kg, PO** | **t1/2 (h)** | **Tmax (h)** | **Cmax (ng/mL)** | **AUClast (h·ng/mL)** | **AUCinf (h·ng/mL)** | **AUC/D (h·kg·ng/mL/mg)** | **AUC Extr (%)** | **MRT (h)** | **Vz_F (L/kg)** | **CL_F (mL/min/kg)** | **F (%)** |
| --- | --- | --- | --- | --- | --- | --- | --- | --- | --- | --- | --- |
| Mouse #1 | 3.55 | 0.083 | 678 | 397 | 433 | 43.29 | 8.28 | 2.15 | 118.29 | 384.99 | 2.19 |
| Mouse #2 | 0.90 | 0.083 | 915 | 557 | 570 | 57.01 | 2.28 | 0.80 | 22.75 | 292.33 | 3.07 |
| Mouse #3 | 1.65 | 0.083 | 158 | 225 | 237 | 23.70 | 5.03 | 2.11 | 100.65 | 703.26 | 1.24 |
| Mean | 2.03 | 0.083 | 584 | 393 | 413 | 41.33 | 5.20 | 1.69 | 80.57 | 460.19 | 2.17 |
| SD | 1.37 | 0.000 | 387 | 166 | 167 | 16.74 | 3.01 | 0.77 | 50.84 | 215.54 | 0.92 |

**C EBS exposure in brain, heart, and plasma 8 h after a single oral dose of 10 mg/kg**

| **Compound** | **Route** | **Dose (mg/kg)** | **Time (h)** | **Subject** | **Brain concentration (ng/g)** | **Heart concentration (ng/g)** | **Plasma concentration (ng/mL)** | **Heart/Plasma ratio** | **Brain/Plasma ratio** |
| --- | --- | --- | --- | --- | --- | --- | --- | --- | --- |
| Ebselen | PO | 10 | 8 | Mouse #1 | BLOQ | BLOQ | 7 | NA | NA |
|  |  |  |  | Mouse #2 | BLOQ | BLOQ | BLOQ | NA | NA |
|  |  |  |  | Mouse #3 | BLOQ | BLOQ | 5 | NA | NA |
|  |  |  |  | Mean | BLOQ | BLOQ | 6 | NA | NA |
|  |  |  |  | SD | NA | NA | NA | NA | NA |

**Table EV1. Pharmacokinetic (PK) analysis of EBS in WT mice.** A. Plasma concentrations of EBS over time after a single oral dose of 10 mg/kg in 3 mice (mean and standard deviation). B. PK parameters of EBS in mouse plasma after a single oral administration (PO) of 10 mg/kg EBS. Cmax, maximum concentration; Tmax, time to reach maximum concentration; t1/2, half-life; AUC, area under the curve; MRT, mean residence time; CL, clearance rate. C. EBS exposure in mouse brain, heart, and plasma 8 h after a single oral administration (PO) of 10 mg/kg EBS. BLOQ, below the limit of quantification.
