# Supplementary material for: Ebselen restores mitochondrial function through metabolic rewiring in Barth syndrome and related mitochondrial diseases
Source: EMBO Mol Med. 2026 Oct 5;18(11):4227–59. doi: 10.1038/s44321-026-00518-1 (PMC13748542; doi:10.1038/s44321-026-00518-1)
Supplement: Supplementary file 15 [file 44321_2026_518_MOESM2_ESM.docx]

| **Protein** | **# peptides** | **Pathway** | **A** | **B** | **C** | **D** | **B/A** | **B/C** | **p-value B/C** | **Cys residues** |
| --- | --- | --- | --- | --- | --- | --- | --- | --- | --- | --- |
| HHO1 | 13 | Cytosolic translation / ribosome / rRNA processing | 2.82E+04 | 2.05E+07 | 7.84E+05 | 4.36E+07 | 728 | 26.2 | 3.26E-04 |  |
| NOP1 | 22 |  | 9.05E+05 | 9.45E+07 | 8.94E+06 | 1.42E+08 | 104 | 10.6 | 1.03E-03 |  |
| RPL37A | 9 |  | 5.40E+06 | 1.79E+07 | 1.79E+06 | 6.89E+07 | 3 | 10.0 | 1.16E-03 |  |
| RPL12A | 10 |  | 5.37E+04 | 3.52E+08 | 3.94E+07 | 5.73E+08 | 6556 | 8.9 | 3.26E-04 |  |
| HAS1 | 30 |  | 1.33E+05 | 4.38E+07 | 6.25E+06 | 8.74E+07 | 330 | 7.0 | 2.43E-03 |  |
| SUI1 | 9 |  | Not detected | 3.51E+07 | 5.30E+06 | 8.26E+07 | NA | 6.6 | 5.04E-03 |  |
| RPS11B | 13 |  | 3.79E+06 | 2.03E+08 | 3.16E+07 | 4.12E+08 | 54 | 6.4 | 2.73E-02 |  |
| RPL34B | 5 |  | 1.37E+07 | 1.36E+08 | 2.16E+07 | 3.33E+08 | 10 | 6.3 | 4.00E-02 |  |
| RPS31 | 14 |  | 1.36E+07 | 6.99E+07 | 1.24E+07 | 1.96E+08 | 5 | 5.7 | 5.62E-02 |  |
| RPL43B | 11 |  | 1.22E+07 | 1.24E+08 | 2.24E+07 | 2.75E+08 | 10 | 5.5 | 1.35E-02 |  |
| RPA190 | 47 |  | 9.78E+04 | 2.41E+07 | 4.87E+06 | 5.03E+07 | 247 | 5.0 | 1.80E-02 |  |
| TEF1; TEF2 | 48 |  | 3.70E+06 | 9.61E+09 | 2.07E+09 | 1.96E+10 | 2595 | 4.6 | 8.94E-04 |  |
| NOB1 | 17 |  | 4.50E+04 | 8.72E+07 | 1.89E+07 | 1.68E+08 | 1939 | 4.6 | 4.18E-07 |  |
| MDS3 | 8 | TOR regulation | Not detected | 1.45E+05 | 5.72E+03 | 2.29E+06 | NA | 25.4 | NA | R |
| SCS2 | 20 |  | 1.43E+05 | 8.05E+07 | 5.69E+06 | 2.79E+08 | 562 | 14.1 | 1.45E-05 | R |
| OSH6 | 29 |  | 2.26E+05 | 1.58E+07 | 1.66E+06 | 3.30E+07 | 70 | 9.5 | 2.96E-03 | R |
| KES1 | 23 |  | 8.64E+04 | 2.83E+07 | 4.71E+06 | 9.60E+07 | 327 | 6.0 | 3.72E-04 | R |
| GGC1 | 19 |  | 2.15E+05 | 2.56E+07 | 5.14E+06 | 4.70E+07 | 119 | 5.0 | 4.23E-02 | R |
| PTP1 | 4 | Signal transduction | 5.73E+03 | 2.08E+07 | 9.44E+05 | 1.24E+07 | 3626 | 22.0 | 1.21E-03 | RR |
| IPP1 | 24 |  | 1.86E+06 | 2.00E+08 | 2.29E+07 | 5.34E+08 | 107 | 8.7 | 2.55E-04 |  |
| GRX5 | 9 | Stress response / redox | 5.51E+03 | 1.14E+07 | 4.43E+05 | 1.58E+07 | 2075 | 25.8 | 4.54E-03 | RR |
| PRX1 | 16 |  | 1.08E+04 | 3.58E+07 | 3.91E+06 | 8.11E+07 | 3322 | 9.2 | 5.99E-04 | RR |
| MDH3 (TCA) | 24 | Mitochondrial functions | 7.36E+03 | 6.06E+07 | 1.87E+06 | 9.38E+07 | 8241 | 32.4 | 3.64E-04 | RR |
| QCR7 (CIII) | 13 |  | 1.22E+04 | 1.14E+08 | 1.04E+07 | 3.92E+08 | 9354 | 10.9 | 1.15E-04 | R |
| FOX2 (FA oxidation) | 35 |  | 4.87E+04 | 2.20E+07 | 2.63E+06 | 5.84E+07 | 451 | 8.4 | 1.38E-02 | RR |
| ACC1 (FA oxidation) | 147 |  | 1.57E+06 | 5.58E+08 | 6.97E+07 | 1.42E+09 | 356 | 8.0 | 1.08E-03 | RR |
| ATP5 (CV) | 17 |  | 2.22E+04 | 1.23E+08 | 1.83E+07 | 2.98E+08 | 5538 | 6.7 | 1.65E-02 | R |
| RVS161 (CoQ) | 20 |  | 1.66E+05 | 3.37E+07 | 6.33E+06 | 1.08E+08 | 203 | 5.3 | 6.16E-03 | R |
| DJP1 (folding) | 30 |  | 3.61E+05 | 1.49E+07 | 2.99E+06 | 3.84E+07 | 41 | 5.0 | 1.25E-02 | R |
| CPS1 (nucleotide) | 24 |  | 6.95E+04 | 4.32E+07 | 8.81E+06 | 8.29E+07 | 622 | 4.9 | 9.45E-04 |  |
| GVP36 | 18 | TGN | 1.24E+04 | 8.59E+07 | 9.77E+06 | 2.05E+08 | 6915 | 8.8 | 1.54E-03 | R |

**Table EV2. List of proteins most specifically targeted by EBS in the BTHS yeast model.** Proteins were filtered to retain only master proteins with a minimum of two unique peptides per protein. Additional selection criteria included enrichment ratios of EBS-B / E-B > 3 and EBS-B / (EBS-B + aC) > 3, as well as statistical significance and low variability between biological replicates. Based on these criteria, the 30 most abundant proteins that specifically bind to EBS in yeast are shown and classified into distinct functional pathways. Proteins are ranked in decreasing order of the B/C enrichment ratio. For each protein, the name, peptide number, enrichment ratios B/A and B/C, and the p-value for B/C are displayed. Experimental conditions are defined as follows: condition A, E-B (empty beads without EBS); condition B, EBS-B (beads coupled to EBS); condition C, active competition with free EBS added prior to incubation with EBS-B beads; condition D, inactive competition with an inactive EBS analog added prior to incubation with EBS-B beads. The potential ability of proteins to interact with EBS based on their cysteine content is indicated: RR denotes high-likelihood targets containing catalytic or highly reactive cysteines essential for protein function; R denotes moderate-likelihood targets containing cysteines or disulfide bonds that may be redox-sensitive but are not directly catalytic, where EBS could affect structure or regulation; all other proteins possess cysteine residues that are poorly accessible, not redox-active, or are absent, making direct interaction with EBS unlikely.
