# Supplementary material for: Ebselen restores mitochondrial function through metabolic rewiring in Barth syndrome and related mitochondrial diseases
Source: EMBO Mol Med. 2026 Oct 5;18(11):4227–59. doi: 10.1038/s44321-026-00518-1 (PMC13748542; doi:10.1038/s44321-026-00518-1)
Supplement: Supplementary file 16 [file 44321_2026_518_MOESM3_ESM.docx]

| **Protein** | **# peptides** | **Pathway** | **A** | **B** | **C** | **B/A** | **B/C** | **P value B/C** | **Cys residues** |
| --- | --- | --- | --- | --- | --- | --- | --- | --- | --- |
| RPS2 | 16 | Cytosolic translation | 1.50E+05 | 1.66E+08 | 2.28E+06 | 1108 | 72.7 | 5.90E-05 |  |
| RPS6 | 9 |  | 1.33E+05 | 8.10E+07 | 6.10E+06 | 610 | 13.3 | 9.74E-04 |  |
| SERBP1 | 27 |  | 5.95E+04 | 1.14E+08 | 1.13E+07 | 1911 | 10.1 | 2.65E-04 |  |
| RPS14 | 11 |  | 1.02E+05 | 1.53E+08 | 1.63E+07 | 1499 | 9.4 | 6.04E-04 |  |
| RPL38 | 7 |  | 2.92E+04 | 8.57E+07 | 9.18E+06 | 2938 | 9.3 | 4.35E-03 |  |
| RPS25 | 5 |  | 8.79E+04 | 2.18E+08 | 2.41E+07 | 2482 | 9.0 | 6.19E-03 |  |
| ELAVL1 | 16 |  | 9.14E+04 | 8.27E+07 | 1.01E+07 | 905 | 8.2 | 6.75E-05 |  |
| EEF1A1 | 27 |  | 1.28E+06 | 1.66E+09 | 2.42E+08 | 1298 | 6.9 | 2.09E-03 |  |
| RPL30 | 8 |  | 1.51E+05 | 1.23E+08 | 1.79E+07 | 814 | 6.9 | 1.75E-04 |  |
| RPS3A | 25 |  | 1.29E+06 | 6.92E+08 | 1.22E+08 | 535 | 5.7 | 8.58E-04 |  |
| RPL12 | 8 |  | 8.79E+04 | 8.83E+07 | 1.70E+07 | 1004 | 5.2 | 1.54E-02 |  |
| EIF2S3 | 24 |  | 8.20E+04 | 1.29E+08 | 2.53E+07 | 1573 | 5.1 | 4.60E-02 |  |
| RPS17 | 11 |  | 1.39E+05 | 2.27E+08 | 4.52E+07 | 1632 | 5.0 | 2.07E-02 |  |
| MEAK7 | 13 | TOR regulation | NA | 1.64E+07 | 9.57E+05 | NA | 17.1 | 1.05E-03 |  |
| TRIM4 | 9 | Signal transduction / scaffolding / trafficking | 1.32E+05 | 2.57E+08 | 2.39E+07 | 1947 | 10.8 | 4.68E-04 |  |
| DCLK1 | 33 |  | 1.38E+05 | 9.34E+07 | 1.39E+07 | 675 | 6.7 | 2.63E-03 |  |
| AP2M1 | 22 |  | 4.64E+05 | 3.63E+08 | 7.18E+07 | 783 | 5.1 | 3.45E-03 |  |
| AKAP2 | 37 |  | 1.14E+05 | 1.22E+08 | 2.64E+07 | 1078 | 4.6 | 2.86E-05 |  |
| CSTB | 4 | Oxidative stress / redox | 8.08E+04 | 1.54E+08 | 3.66E+06 | 1903 | 42.0 | 3.28E-04 | **R** |
| GSR | 22 |  | 3.35E+04 | 1.25E+08 | 3.97E+06 | 3727 | 31.5 | 5.50E-03 | **RR** |
| PRDX1 | 21 |  | 2.46E+06 | 1.30E+09 | 5.62E+07 | 527 | 23.1 | 6.11E-05 | **RR** |
| TXNDC5 | 23 | Folding / chaperone | 2.81E+05 | 1.64E+08 | 5.22E+06 | 584 | 31.5 | 4.27E-04 | **RR** |
| PDIA6 | 20 |  | 8.97E+04 | 1.87E+08 | 1.78E+07 | 2079 | 10.5 | 1.05E-03 | **RR** |
| ATP5PD (CV) | 15 | Mitochondrial functions | 5.61E+04 | 7.77E+07 | 2.16E+06 | 1385 | 35.9 | 3.20E-04 |  |
| PDP1 (TCA) | 6 |  | NA | 1.78E+06 | 1.41E+05 | NA | 12.6 | 6.94E-03 |  |
| HSPB6 | 10 | Cytoskeleton / structural / contraction | NA | 7.63E+07 | 5.74E+06 | NA | 13.3 | 5.85E-04 | **R** |
| SYNPO2 | 29 |  | 6.40E+04 | 1.40E+08 | 1.24E+07 | 2190 | 11.3 | 5.93E-05 | **R** |
| FLNC | 160 |  | 7.04E+05 | 9.78E+08 | 1.76E+08 | 1389 | 5.6 | 1.52E-03 | **R** |
| CSRP1 | 12 |  | 9.31E+04 | 7.69E+07 | 1.50E+07 | 826 | 5.1 | 4.59E-05 | **R** |
| PALLD | 41 |  | 6.78E+04 | 1.25E+08 | 2.51E+07 | 1840 | 5.0 | 1.06E-02 | **R** |
| ANXA2 | 40 |  | 1.14E+07 | 1.29E+10 | 2.81E+09 | 1133 | 4.6 | 1.14E-02 | **R** |

**Table EV3. List of proteins most specifically targeted by EBS in BTHS myoblasts.**

Same legend as Table EV2.
