# Supplementary material for: Ebselen restores mitochondrial function through metabolic rewiring in Barth syndrome and related mitochondrial diseases
Source: EMBO Mol Med. 2026 Oct 5;18(11):4227–59. doi: 10.1038/s44321-026-00518-1 (PMC13748542; doi:10.1038/s44321-026-00518-1)
Supplement: Supplementary file 17 [file 44321_2026_518_MOESM4_ESM.docx]

| **Protein** | **# peptides** | **Pathway** | **A** | **B** | **C** | **B/A** | **B/C** | **p-value B/C** | **Cys residues** |
| --- | --- | --- | --- | --- | --- | --- | --- | --- | --- |
| Rpl30 | 8 | **Cytosolic translation** | 1.28E+05 | 8.22E+07 | 6.27E+06 | 640 | 13.1 | 2.95E-02 |  |
| Rpl12 | 7 |  | 1.91E+04 | 2.96E+07 | 5.13E+06 | 1554 | 5.8 | 3.24E-03 |  |
| Rps25 | 4 |  | 2.10E+04 | 2.10E+07 | 5.23E+06 | 1002 | 4.0 | 3.34E-02 |  |
| Cdc42 | 10 | **TOR / TOR regulation** | 2.42E+04 | 2.91E+07 | 4.86E+06 | 1206 | 6.0 | 3.13E-03 | **R** |
| Prkar1a | 18 |  | NA | 2.63E+07 | 5.40E+06 | NA | 4.9 | 8.55E-03 | **R** |
| Gnb1 | 14 |  | NA | 2.20E+07 | 4.59E+06 | NA | 4.8 | 1.05E-02 | **R** |
| Gnb2 | 17 |  | NA | 2.17E+07 | 5.20E+06 | NA | 4.2 | 9.64E-03 | **R** |
| Rac1 | 11 |  | 2.81E+04 | 3.53E+07 | 9.41E+06 | 1258 | 3.8 | 1.39E-02 | **R** |
| Prdx6 | 16 | **Redox metabolism** | 1.15E+05 | 8.68E+07 | 1.57E+07 | 752 | 5.5 | 1.87E-02 | **RR** |
| Pgls | 14 |  | 4.72E+04 | 2.42E+07 | 5.48E+06 | 511 | 4.4 | 2.89E-02 | **RR** |
| Hspb6 | 10 | **Folding** | NA | 2.99E+07 | 6.64E+06 | NA | 4.5 | 1.16E-02 | **R** |
| Dnajb4 | 15 |  | 2.81E+04 | 2.55E+07 | 5.96E+06 | 909 | 4.3 | 1.79E-02 | **R** |
| St13 | 11 |  | 1.48E+04 | 3.63E+07 | 9.99E+06 | 2447 | 3.6 | 1.98E-02 | **R** |
| Hspa4 | 34 |  | 6.27E+04 | 3.80E+07 | 1.09E+07 | 605 | 3.5 | 4.94E-02 | **R** |
| Cct5 |  |  | 6.08E+03 | 2.08E+07 | 6.54E+06 | 3427 | 3.2 |  | **R** |
| Ndufa2 (CI) | 5 | **Mitochondrial functions** | 1.90E+04 | 7.49E+07 | 6.43E+06 | 3943 | 11.6 | 5.12E-02 |  |
| Fam162a (apoptosis) | 9 |  | NA | 3.41E+07 | 4.64E+06 | NA | 7.3 | 6.60E-03 | **R** |
| Ndufs6 (CI) | 9 |  | 2.06E+04 | 2.54E+08 | 3.52E+07 | 12304 | 7.2 | 1.80E-02 |  |
| Atp5pd (CV) | 14 |  | 4.15E+05 | 6.83E+08 | 1.30E+08 | 1645 | 5.3 | 1.37E-02 |  |
| Uqcc2 (CIII) | 8 |  | 1.42E+04 | 4.14E+07 | 8.08E+06 | 2912 | 5.1 | 1.12E-02 | **R** |
| Cpt1b (FA oxidation) | 29 |  | 1.53E+04 | 4.67E+07 | 1.11E+07 | 3047 | 4.2 | 2.95E-02 | **R** |
| Fdxr (iron-sulfur cluster) | 17 |  | 1.73E+04 | 1.82E+07 | 4.92E+06 | 1050 | 3.7 | 8.58E-03 | **RR** |
| Tomm20 (import) | 4 |  | 3.95E+04 | 2.18E+07 | 6.15E+06 | 550 | 3.5 | 2.22E-02 |  |
| Mpc2 (TCA) | 9 |  | 9.40E+03 | 2.85E+07 | 8.21E+06 | 3034 | 3.5 | 6.79E-02 |  |
| Acot2 (FA oxidation) | 24 |  | 3.25E+04 | 6.52E+07 | 1.92E+07 | 2004 | 3.4 | 2.20E-03 | **R** |
| Ppif (apoptosis) | 8 |  | 1.20E+05 | 1.02E+08 | 3.12E+07 | 848 | 3.3 | 2.03E-02 | **RR** |
| Dld (TCA/PDH) | 26 |  | 1.54E+05 | 6.22E+08 | 1.97E+08 | 4034 | 3.2 | 2.74E-02 | **RR** |
| Cox20 (CIV) | 6 |  | 9.49E+03 | 2.48E+07 | 8.11E+06 | 2615 | 3.1 | 8.29E-03 | **R** |
| Nfs1 (iron-sulfur cluster) | 19 |  | 1.78E+04 | 2.25E+07 | 7.23E+06 | 1260 | 3.1 | 1.67E-02 | **RR** |
| Tnnc1 | 6 | **Cardiac contraction** | NA | 2.72E+07 | 2.74E+06 | NA | 9.9 | 1.05E-02 |  |
| Mybpc3 | 70 |  | 5.50E+05 | 7.06E+08 | 1.61E+08 | 1284 | 4.4 | 1.04E-02 |  |
| Nexn | 21 |  | 2.58E+04 | 3.07E+07 | 8.88E+06 | 1187 | 3.5 | 4.34E-02 |  |
| Myl3 | 17 |  | 1.56E+05 | 1.28E+08 | 4.14E+07 | 821 | 3.1 | 3.24E-03 |  |
| Hba | 9 | Hemoglobin | 4.01E+05 | 1.48E+09 | 6.73E+07 | 3675 | 21.9 | 3.26E-02 |  |

**Table EV4. List of proteins most specifically targeted by EBS in the BTHS mouse model.**

Same legend as Table EV2.
