# Supplementary material for: Ebselen restores mitochondrial function through metabolic rewiring in Barth syndrome and related mitochondrial diseases
Source: EMBO Mol Med. 2026 Oct 5;18(11):4227–59. doi: 10.1038/s44321-026-00518-1 (PMC13748542; doi:10.1038/s44321-026-00518-1)
Supplement: Supplementary file 18 [file 44321_2026_518_MOESM5_ESM.docx]

| **Protein** | **Accession** | **# Peptides** | **Pathway** | **ratio (T/C)** | **p-value** | **Cys residues** |
| --- | --- | --- | --- | --- | --- | --- |
| **Downregulated** | | | | | | |
| RPL22B | P56628 | 2 | **Cytosolic translation / amino acid metabolism** | 0.52 | 1.87E-02 |  |
| NOP14 | Q99207 | 3 |  | 0.55 | 7.94E-03 |  |
| SAM4 | Q08985 | 7 |  | 0.58 | 1.09E-02 | **R** |
| RPP1 | P38786 | 3 |  | 0.59 | 1.23E-02 |  |
| RRP8 | P38961 | 3 |  | 0.61 | 1.68E-02 |  |
| GRS2 | Q06817 | 3 |  | 0.61 | 1.61E-02 |  |
| RPS29A | P41057 | 4 |  | 0.62 | 1.81E-02 |  |
| RRP15 | Q06511 | 2 |  | 0.63 | 2.01E-02 |  |
| IMP4 | P53941 | 5 |  | 0.64 | 2.31E-02 |  |
| RPL11B | Q3E757 | 7 |  | 0.68 | 1.72E-03 |  |
| ALD3 | P54114 | 23 | **TCA / TCA precursors** | 0.57 | 9.75E-03 | **R** |
| GPP1 | P41277 | 13 |  | 0.65 | 9.84E-05 |  |
| PIS1 | P06197 | 3 | **Fatty acids** | 0.70 | 3.12E-03 |  |
| TGL1 | P34163 | 9 |  | 0.71 | 4.92E-02 | **R** |
| CYC7 | P00045 | 2 | **Iron homeostasis / heme proteins** | 0.55 | 2.77E-05 |  |
| ISU1 | Q03020 | 2 |  | 0.59 | 1.59E-05 | **RR** |
| DRE2 | P36152 | 4 |  | 0.65 | 2.61E-02 | **RR** |
| GAL3 | P13045 | 5 | **Glycolysis** | 0.00 | NA |  |
| GAL2 | P13181 | 12 |  | 0.57 | 5.82E-08 |  |
| GAL7 | P08431 | 13 |  | 0.57 | 6.43E-07 |  |
| GAL10 | P04397 | 29 |  | 0.57 | 3.46E-07 |  |
| MAN2 | P0CX09 | 11 |  | 0.60 | 1.52E-06 |  |
| GAL1 | P04385 | 23 |  | 0.66 | 5.87E-04 |  |
| SRX1 | P36077 | 3 | **Redox metabolism** | 0.55 | 6.56E-04 | **RR** |
| AAD16 | Q02895 | 2 |  | 0.60 | 1.60E-03 | **R** |

| **Protein** | **Accession** | **# Peptides** | **Pathway** | **ratio (T/C)** | **p-value** | **Cys residues** |
| --- | --- | --- | --- | --- | --- | --- |
| **Upregulated** | | | | | | |
| MBA1 | P38300 | 2 | **Cytosolic translation** | 1.76 | 2.31E-05 |  |
| ADY2 | P25613 | 2 | **TCA / TCA precursors** | 4.99 | 5.76E-16 |  |
| ACS1 | Q01574 | 38 |  | 2.00 | 5.16E-10 | **R** |
| POX1 | P13711 | 17 |  | 1.89 | 1.96E-02 |  |
| FAA2 | P39518 | 10 |  | 1.86 | 2.23E-02 |  |
| CIT2 | P08679 | 21 |  | 1.75 | 1.88E-06 |  |
| ICL2 | Q12031 | 17 |  | 1.69 | 4.12E-07 | **R** |
| CAT2 | P32796 | 23 |  | 1.69 | 1.08E-05 |  |
| ADH2 | P00331 | 18 |  | 1.67 | 1.59E-05 |  |
| FOX2 | Q02207 | 37 |  | 1.65 | 2.60E-05 |  |
| CIT3 | P43635 | 11 |  | 1.64 | 2.32E-05 |  |
| YAT1 | P80235 | 22 |  | 1.61 | 9.84E-05 |  |
| YGR067C | P53243 | 4 |  | 1.61 | 5.17E-02 |  |
| ACO1 | P19414 | 42 |  | 1.59 | 1.39E-04 |  |
| PDH1 | Q12428 | 20 |  | 1.52 | 1.07E-03 | **R** |
| MLS1 | P30952 | 31 |  | 1.50 | 1.60E-03 | **R** |
| ADH5 | P38113 | 7 |  | 1.50 | 3.92E-03 |  |
| IDP2 | P41939 | 28 |  | 1.50 | 3.32E-03 |  |
| PCK1 | P10963 | 31 |  | 1.50 | 9.22E-03 |  |
| MDH2 | P22133 | 10 |  | 1.50 | 5.03E-03 | **R** |
| FAR11 | P53917 | 2 | **TOR** | 2.70 | 1.99E-04 |  |
| PTC1 | P35182 | 2 |  | 2.55 | 2.15E-03 |  |
| RRD1 | P40454 | 2 |  | 2.45 | 2.96E-03 |  |
| LST8 | P41318 | 2 |  | 1.57 | 2.20E-04 | **R** |
| PPH22 | P23595 | 4 | **Folding / degradation** | 1000 | NA |  |
| UBC7 | Q02159 | 2 |  | 1.72 | 3.63E-02 | **RR** |
| YPR063C | Q12160 | 2 |  | 1.66 | 4.31E-04 |  |
| FBP1 | P09201 | 17 | **Glycolysis** | 1.40 | 4.01E-02 |  |

**Table EV5. Proteomic analysis in the BTHS yeast model.** Proteins significantly downregulated (left panel) or upregulated (right panel) following EBS treatment in the BTHS yeast model are shown. Proteins were selected based on a minimum fold change of 1.5, either decrease or increase. Data were filtered to retain master proteins identified with at least two unique peptides, statistical significance, and low inter-replicate variability. This analysis reflects a global proteomic response rather than changes at the level of individual proteins. Moderate but reproducible fold changes consistently affecting the same pathways were considered biologically relevant. Proteins are ranked in decreasing order of the T/C ratio. Pathways, accession numbers and peptide counts are annotated for each protein. Ratio values of 1000 or 0.001 were arbitrarily assigned when a protein was detected exclusively in one condition: a value of 1000 indicates that the protein was present in the EBS-treated condition and absent in the control condition, whereas a value of 0.001 indicates that the protein was detected only in the control condition and not in the EBS-treated condition. These values do not represent quantitative measurements but reflect condition-specific presence or absence. The potential ability of proteins to interact with ebselen based on their cysteine content is indicated: RR denotes high-likelihood targets containing catalytic or highly reactive cysteines essential for protein function (ISU1, DRE2, SRX1, UBC7); R denotes moderate-likelihood targets containing cysteines or disulfide bonds that may be redox-sensitive but are not directly catalytic, where EBS could affect structure or regulation (SAM4, ALD3, TGL1, AAD16, MDH2, ACS1, ICL2, LST8); all other proteins possess cysteine residues that are poorly accessible, not redox-active, or are entirely absent, making direct interaction with EBS unlikely.
