# Supplementary material for: Ebselen restores mitochondrial function through metabolic rewiring in Barth syndrome and related mitochondrial diseases
Source: EMBO Mol Med. 2026 Oct 5;18(11):4227–59. doi: 10.1038/s44321-026-00518-1 (PMC13748542; doi:10.1038/s44321-026-00518-1)
Supplement: Supplementary file 19 [file 44321_2026_518_MOESM6_ESM.docx]

**Downregulated proteins**

| **Protein** | **Accession** | **# peptides** | **Pathway** | **ratio (T/C)** | **p value** | **Detection pattern** | **Replicate detection: T (present); C (absent) with abundance >50000** | **Cys residues** |
| --- | --- | --- | --- | --- | --- | --- | --- | --- |
| **Downregulated proteins** |  |  |  |  |  |  |  |  |
| Pdk4 | O70571 | 11 | **TCA** | 0.4 | 8.08E-03 | NA | NA |  |
| Fam98b | Q80VD1 | 3 | **Translation / RNA processing** | 0.001 | NA | only in control | 3;3 |  |
| Mrps2 | Q924T2 | 2 |  | 0.001 | NA | only in control | 3;2 |  |
| Chtop | Q9CY57 | 2 |  | 0.001 | NA | only in control | 3;2 |  |
| Srsf10 | Q9R0U0 | 3 |  | 0.001 | NA | only in control | 3;2 |  |
| Srsf4 | Q8VE97 | 5 |  | 0.001 | NA | only in control | 3;2 |  |
| Gpsm1 | Q6IR34 | 5 | **mTOR regulation / AA metabolism (lysosome)** | 0.001 | NA | only in control | 3;2 |  |
| Enoph1 | Q8BGB7 | 2 |  | 0.001 | NA | only in control | 3;2 |  |
| Rhob | P62746 | 4 |  | 0.001 | NA | only in control | 3;2 |  |
| Nae1 | Q8VBW6 | 3 |  | 0.001 | NA | only in control | 3;2 | RR |
| Arfgap1 | Q9EPJ9 | 2 |  | 0.001 | NA | only in control | 3;2 |  |
| Tspan7 | Q62283 | 2 |  | 0.001 | NA | only in control | 3;3 |  |
| Cpq | Q9WVJ3 | 4 |  | 0.001 | NA | only in control | 3;2 |  |
| Nagpa | Q8BJ48 | 2 |  | 0.001 | NA | only in control | 3;3 |  |
| Rbp4 | Q00724 | 4 |  | 0.45 | 6.48E-02 |  | NA |  |
| Gda | Q9R111 | 3 | **Purine pathway** | 0.001 | NA | only in control | 3;3 |  |
| Otud4 | B2RRE7 | 2 | **Folding / degradation** | 0.001 | NA | only in control | 3;2 | RR |
| Arih1 | Q9Z1K5 | 2 |  | 0.001 | NA | only in control | 3;3 | RR |
| Bpifa2 | P07743 | 6 |  | 0.001 | NA | only in control | 3;2 | R |
| Clec1b | Q9JL99 | 2 | **Inflammation / immune** | 0.001 | NA | only in control | 3;3 | R |
| Cuecd1 | Q8R3V6 | 2 |  | 0.001 | NA | only in control | 3;3 |  |
| Ubr4 | A2AN08 | 4 |  | 0.001 | NA | only in control | 3;3 |  |
| Serpina1e | Q00898 | 16 |  | 0.37 | 1.86E-02 | in C and T | NA | R |
| Ppbp | Q9EQI5 | 2 |  | 0.45 | 1.86E-02 | in C and T | NA |  |
| Stim1 | P70302 | 6 | **Muscle contraction / structure** | 0.001 | NA | only in control | 3;2 | R |
| Coro1b | Q9WUM3 | 2 |  | 0.001 | NA | only in control | 3;3 |  |
| Sntb1 | Q99L88 | 4 |  | 0.001 | NA | only in control | 3;3 |  |
| Ptgds | O09114 | 4 |  | 0.001 | NA | only in control | 3;2 |  |
| Vwa1 | Q8R2Z5 | 5 |  | 0.44 | 3.46E-02 | in C and T | NA |  |
| Abcd3 | P55096 | 3 | **Fatty acids / lipids** | 0.001 | NA | only in control | 3;3 |  |
| Hsd17b11 | Q9EQ06 | 2 |  | 0.001 | NA | only in control | NA |  |
| Fasn | P19096 | 15 |  | 0.34 | 2.03E-03 | in C and T | NA |  |
| Blvra | Q9CY64 | 3 |  | 0.34 | 8.26E-03 | in C and T | NA | RR |
| Tango2 | P54797 | 6 |  | 0.4 | 1.00E-02 | in C and T | NA |  |
| Cyp27a1 | Q9DBG1 | 2 |  | 0.44 | 7.24E-02 | in C and T | NA | R |
| Cenpv | Q9CXS4 | 3 | **Cytoskeleton** | 0.001 | NA | only in control | 3;2 |  |
| Jpt1 | P97825 | 2 |  | 0.001 | NA | only in control | 3;3 |  |
| Gbe1 | Q9D6Y9 | 6 | **Glycolysis** | 0.001 | NA | only in control | 3;3 |  |
| Slc16a1 | P53986 | 7 |  | 0.5 | 1.78E-02 | in C and T | NA |  |
| Mtatp6 | P00848 | 2 | **OXPHOS** | 0.001 | NA | only in control | 3;3 |  |
| Dmac1 | Q9CQ00 | 2 |  | 0.001 | NA | only in control | 3;2 |  |
| Gpd2 | Q64521 | 9 |  | 0.38 | 1.78E-02 | in C and T | NA |  |
| Rtn4ip1 | Q924D0 | 8 |  | 0.39 | 1.37E-02 | in C and T | NA |  |
| Mcur1 | Q9CXD6 | 4 | **mt import** | 0.001 | NA | only in control | 3;2 |  |
| Maob | Q64133 | 16 | **Redox metabolism** | 0.25 | 3.58E-04 | in C and T | NA | R |
| Xdh | Q00519 | 4 |  | 0.4 | 1.91E-02 | in C and T | NA | RR |
| Memo1 | Q91VH6 | 2 | **Copper homeostasis** | 0.001 | NA | only in control | 3;2 | R |
| F13a1 | Q8BH61 | 3 | **Blood coagulation** | 0.001 | NA | only in control | 3;2 | R |
| Gp1bb | P56400 | 2 |  | 0.001 | NA | only in control | 3;2 | R |
| Vwf | Q8CIZ8 | 3 |  | 0.001 | NA | only in control | 3;2 | R |

**Upregulated proteins**

| **Protein** | **Accession** | **# peptides** | **Pathway** | **ratio (T/C)** | **p value** | **Detection pattern** | **Replicate detection: T (present); C (absent) with abundance >50000** | **Cys residues** |
| --- | --- | --- | --- | --- | --- | --- | --- | --- |
| **Upregulated proteins** |  |  |  |  |  |  |  |  |
| Pdp1 | Q3UV70 | 3 | **TCA** | 1000 | NA | only in treated | 3;3 |  |
| Trir | Q9D735 | 2 | **Translation** | 1000 | NA | only in treated | 2;3 | RR |
| Commd8 | Q9CZG3 | 3 | **mTOR regulation / AA metabolism (lysosome)** | 1000 | NA | only in treated | 2;3 |  |
| Ccdc43 | Q9CR29 | 2 |  | 1000 | NA | only in treated | 2;3 |  |
| Ctsl | P06797 | 3 |  | 1000 | NA | only in treated | 3;3 | RR |
| Txndc9 | Q9CQ79 | 2 | **Folding / degradation** | 1000 | NA | only in treated | 3;3 | RR |
| Ttc4 | Q8R3H9 | 2 |  | 1000 | NA | only in treated | 3;3 |  |
| Selenos | Q9BCZ4 | 2 |  | 1000 | NA | only in treated | 2;3 | RR |
| Igkv12-46 [Ig kappa chain V-III] | A0A140T8P6 | 3 | **Inflammation / immune** | 1000 | NA | only in treated | 2;3 | R |
| Vcan | Q62059 | 2 |  | 1000 | NA | only in treated | 3;3 | R |
| Aven | Q9D9K3 | 2 | **Apoptosis** | 1000 | NA | only in treated | 2;3 | R |
| Dffa | O54786 | 3 |  | 1000 | NA | only in treated | 2;3 | R |
| Ppp1r9b | Q6R891 | 2 |  | 1000 | NA | only in treated | 2;3 | R |
| Col1a1 | P11087 | 10 | **Muscle contraction / structure** | 3.1 | 1.51E-03 | in C and T | NA |  |
| Bin1 | O08539 | 9 |  | 2.3 | 3.06E-02 | in C and T | NA |  |
| Lcat | P16301 | 2 | **Fatty acids** | 2.9 | 1.77E-03 | in C and T | NA | RR |
| Washc3 | Q9CR27 | 2 | **Cytoskeleton (actin/tubulin)** | 3.8 | 6.33E-04 | in C and T | NA |  |
| Tubb3 | Q9ERD7 | 18 |  | 3.4 | 2.01E-03 | in C and T | NA |  |
| Sec16a | E9QAT4 | 2 | **TGN / Vesicle trafficking** | 1000 | NA | only in treated | 2;3 |  |
| Golga1 | Q9CW79 | 3 |  | 1000 | NA | only in treated | 2;3 |  |
| Vamp2 | P63044 | 5 |  | 2.3 | 3.28E-02 | in C and T | NA |  |

**Table EV6. Proteomic analysis in the BTHS mouse model.** Proteins significantly downregulated or upregulated following EBS treatment in the BTHS mouse model are shown. Proteins were selected based on a minimum fold change of 2 (increase or decrease) and filtered to retain master proteins identified with at least two unique peptides, statistical significance, and low inter-replicate variability. Proteins detected in treated mice (T) versus control mice (C) are shown with accession numbers, pathways, T/C ratios, p-values, detection patterns, peptide counts, replicate detection, and cysteine content. RR denotes high-likelihood EBS targets containing catalytic or highly reactive cysteines; R denotes moderate-likelihood targets containing potentially redox-sensitive cysteines or disulfide bonds. Detection patterns indicate whether proteins were observed exclusively in treated (T) or control (C) samples. This analysis was restricted to proteins detected exclusively in either condition (C or T); only proteins detected in at least two of three replicates, with abundances above 50,000, were considered.
